# Long Femoropopliteal Lesions Challenge the Limits of Endovascular Technique: Contemporary Systematic Review and Meta-analysis

**DOI:** 10.1016/j.jscai.2025.104158

**Published:** 2026-02-03

**Authors:** Sameh Sayfo, Anne M. Ryschon, Ami Sood, Abigail M. Garner, Peter A. Soukas, Prakash Krishnan, Jan B. Pietzsch, Peter A. Schneider

**Affiliations:** a Department of Interventional Cardiology, Baylor Scott & White The Heart Hospital – Plano, Plano, Texas; b Wing Tech Inc., Menlo Park, California; c Endologix, Campbell, California; d Brown University Health Cardiovascular Institute, Providence, Rhode Island; e The Zena and Michael A Wiener Cardiovascular Institute, Icahn School of Medicine at Mount Sinai, New York, New York; f Department of Surgery, University of California, San Francisco, San Francisco, California

**Keywords:** endovascular, long lesions, meta-analysis, revascularization, systematic search, TASC D

## Abstract

**Background:**

Traditionally, patients with symptomatic femoropopliteal artery occlusive disease have been treated surgically. However, a growing body of clinical evidence has emerged supporting endovascular treatment in patients with long lesions. The objective of this systematic search and meta-analysis was to assess contemporary outcomes in patients undergoing endovascular treatment for long femoral-popliteal lesions.

**Methods:**

An updated systematic search of Embase, including MEDLINE, identified relevant records published between January 2000 and March 2023 reporting a minimum of 1-year follow-up of endovascular revascularization in patients with mean lesion length ≥20 cm. A pooled meta-analysis was performed using a random-effects model for all longitudinal cumulative event rates. Subgroup analyses explored stratification by lesion length, intervention, study design, and outcome definition. Quality of evidence and risk of bias assessments were conducted.

**Results:**

A total of 28 records, 2338 limbs, and 2311 patients, were analyzed. The pooled cumulative event rates (95% CI) for primary patency were as follows: 0.85 (95% CI, 0.81-0.89), 0.67 (95% CI, 0.61-0.73), 0.48 (95% CI, 0.38-0.57), and 0.42 (95% CI, 0.33-0.52); secondary patency: 0.90 (95% CI, 0.85-0.93), 0.84 (95% CI, 0.77-0.89), 0.72 (95% CI, 0.64-0.78), 0.63 (95% CI, 0.52-0.73); and freedom from target lesion revascularization: 0.93 (95% CI, 0.89-0.96), 0.79 (95% CI, 0.74-0.83), 0.68 (95% CI, 0.62-0.73), and 0.63 (95% CI, 0.57-0.69) at 6, 12, 24, and 36 months, respectively. Select subgroup analyses showed clinically relevant variation in event rates.

**Conclusions:**

This systematic search and meta-analysis provides important insight into the performance of endovascular revascularization in patients with long lesions. These findings will need to be considered as treatment decisions are made.

## Introduction

Open surgical bypass has long been regarded as the preferred method of revascularization for patients with long femoropopliteal lesions.^1^^,^^2^ Recent, multisocietal clinical practice guidelines have highlighted the viability and potential use of endovascular revascularization in specific patient populations, especially those deemed as high-risk surgical candidates.^3^, ^4^, ^5^, ^6^ Technological advancements in endovascular revascularization techniques, such as drug-coated balloons and drug-eluting stents, have encouraged clinicians to consider endovascular therapy in many patients with long lesions. There is a growing body of clinical evidence in support of endovascular revascularization in patients with long lesions, which may help to inform decision making about endovascular versus surgical treatment. The objective of this analysis was to conduct an updated systematic literature search and meta-analysis to systematically assess longitudinal outcome data after endovascular revascularization in patients with long lesions (TransAtlantic Inter-Society Consensus [TASC] C/D) in contemporary clinical practice.

## Methods

This systematic search and meta-analysis was conducted and are reported in accordance with Preferred Reporting Items for Systematic reviews and Meta-Analyses (PRISMA) guidelines (see Supplementary Material S1).^7^ Evaluated outcomes included primary patency, assisted primary patency, secondary patency, mortality, limb salvage, and freedom from target lesion revascularization (ffTLR).

### Search strategy

An update of a previously published search was executed in Embase—which includes Medline, the PubMed core—with subsequent searches of gray literature conducted using PubMed and Google Scholar.^8^ The total search period spanned from year 2000 to March 2023, and the search strategy was intentionally broad, using the following search terms: “superficial femoral artery,” “femoropopliteal,” “long,” and “TASC.”^8^ See Supplementary Material S2 for the detailed search strategy.

### Selection criteria

Included records were those with a patient population aged >18 years with long, TASC D lesions (≥20 cm in length) who underwent femoropopliteal artery revascularization via an endovascular approach. Cohorts including a mix of TASC C/D lesions were considered as long as the mean lesion length (MLL) was ≥20 cm. Both clinical presentations of claudication and chronic limb-threatening ischemia (CLTI) were included. Systematic reviews, conference materials, or registry-sourced analyses were excluded; however, no further exclusions were made on the basis of study design. Duplicate records or analyses were excluded. A minimum of 1 year follow-up for ≥1 primary outcome was required. No exclusions were made based on the endovascular technique employed as long as the device was US Food and Drug Administration-approved. Studies with an overall sample <50 or subgroup <20 patients were excluded, as well as studies with indistinct reporting of outcomes in terms of lesion classification or length. The review was limited to records in English.

### Selection of relevant records

Records of the updated search from January 2019 to March 2023 were screened independently by 2 reviewers (J.B.P. and A.M.R.) by applying the inclusion and exclusion criteria. Studies selected for full-text review from the updated search were then combined with studies deemed relevant in the prior 2000 to June 2019 search. The combined set of full texts were then reviewed independently by the 2 reviewers. Any conflicts were discussed and jointly resolved between the 2 reviewers. The review was conducted using Covidence systematic review software (Veritas Health Innovation).

### Data extraction

Data extraction was independently completed and cross-checked by the 2 reviewers. Relevant cohort characteristics and study details, including location and length of lesion and the type of intervention pursued, were all collected. Data was extracted for the entire study follow-up duration or up to 5 years. The primary outcomes analyzed included primary patency, assisted primary patency, secondary patency, mortality, limb salvage, and ffTLR.

### Quality appraisal assessments

Risk of bias was assessed in accordance with established methods provided by the Cochrane Collaboration, with the domains of bias assessed determined based on the underlying study design.^9^^,^^10^ Risk of bias was evaluated against key criteria: random sequence generation; allocation concealment; blinding of participants, personnel, and outcomes; incomplete outcome data; selective outcome reporting; and other sources of bias for randomized controlled trials (RCTs), while the criteria was amended slightly to accommodate non-RCTs included in the review to account for any bias due to confounding.^9^, ^10^, ^11^, ^12^ The following judgments were used for RCTs and non-RCTs: low, moderate (some concerns), or high risk and low, moderate, and serious, respectively.

Statistical heterogeneity was evaluated based on a qualitative assessment of the between-study heterogeneity (*I*^2^) and between-study variance (τ^2^) as well as through the derivation of prediction intervals as means to determine the expected range of the true effect.^13^ Funnel plots, including the transformed effect size (x-axis) and sample size (y-axis) were used to evaluate small study effects and publication bias.^14^ Furthermore, Egger’s test for publication bias was conducted, with a significant result (*P* < .05) indicating the presence of publication bias.^15^

Grading of Recommendations Assessment, Development and Evaluation (GRADE) quality of evidence assessments were performed for each outcome reported, informed by a qualitative assessment of study characteristics and quantitative evaluation of the pooled data, summary statistics, and all quality appraisal assessments performed. In line with GRADE criteria, high-quality outcomes are assumed to fall close to the true effect measure, moderate quality remains close to true effect with the potential for substantial difference, and low quality assumes there is likely to be substantial differences between the pooled and true measures.^16^

### Outcomes and statistical analyses

The primary analysis outcomes were the cumulative pooled event rates at 6, 12, 24, and 36 months derived from the meta-analyses performed for primary patency, secondary patency, assisted primary patency, mortality, limb salvage, and ffTLR using a random-effects model. Based on available data, 48- and 60-month meta-analyzed pooled estimates were also calculated. Meta-analyses were informed by study-reported cumulative event rates and standard error estimates. Where not reported, standard error estimates were derived from reported confidence intervals or manual derivation. A distinction was made in the unit of measure between number of patients and number of limbs, based on the outcome evaluated and information available; number of patients and limbs were assumed to be equivalent if no distinction was made in the text. Given the broad scope of the search strategy executed and anticipated degree of underlying heterogeneity, a logit or Freeman-Tukey double arcsine transformation, in the case of extreme values, was applied to all proportions before pooling, as means to stabilize the variance. Pooled estimates were subsequently back-transformed for interpretability.

In addition to pooled analyses of the overall data, subgroup analyses were explored on the basis of MLL, outcome definition, study design, intervention, and publication year. The correlation between lesion length and 12-month event rates was further explored for all studies reporting a MLL, with stratification by intervention groupings, consisting of drug-eluting stents, covered stents, bare metal stents, drug-coated balloons, and other combination therapy. Results are discussed for primary patency. The definition recognized for primary outcomes varied across reporting studies, namely as it pertained to primary patency. In line with Society for Vascular Surgery (SVS) reporting standards, 12-month pooled estimates were derived from groupings based on SVS-recommended definitions of primary (uninterrupted patency of treated vessel with no restenosis ≥50%), secondary (occluded vessel successfully reopened with intervention), and assisted primary (patency achieved through repeat procedure, with no occlusion at primary site) patency (group 1), compared with variations of and deviations from the aforementioned definitions (group 2), and no definition reported (group 3).^17^ ffTLR results were further stratified to make a distinction between studies reporting clinically driven ffTLR. Study-reported definitions can be reviewed in Supplementary Material S3. Studies were stratified by primary treatment modality, with groupings consisting of bare metal stents, covered stents, drug-coated balloons, drug-eluting stents, and other. The grouping assignments are reflected in Table 1.^18^, ^19^, ^20^, ^21^, ^22^, ^23^, ^24^, ^25^, ^26^, ^27^, ^28^, ^29^, ^30^, ^31^, ^32^, ^33^, ^34^, ^35^, ^36^, ^37^, ^38^, ^39^, ^40^, ^41^, ^42^, ^43^, ^44^, ^45^ Additional subgroup analyses explored the impact of disease severity on resulting outcomes (stratifying studies by mixed [claudicant and CLTI] vs CLTI-only lesions), study design (with stratification on the basis of RCT vs non-RCT), and publication year (with 2018 used as a threshold representative of the modern endovascular era). For each outcome, a mixed-effects meta-regression was conducted to examine the effect of time on the pooled outcomes. All analyses were conducted using R (R Core Team, 2024).^46^, ^47^, ^48^

**Table 1:** Characteristics of included studies.

Study design	Study	Setting	Intervention	MLL, cm	N (limbs)	N (patients)	Age (mean, y)	% Male	HTN	DM
Non-RCT	AbuRahma et al^18^, 2019 (TASC D cohort)	United States (2010-2017)	DCB	Not detailed	81	81	68	46%	72%	53%
	AbuRahma et al^19^, 2022 (TASC D cohort)	United States (2012-2018)	DES	28.5	47	47	69	57%	57%	43%
	Astarcıoglu et al^20^, 2017	Turkey (2013-2015)	BMS	33.0 (median)	52	48	69	79%	77%	48%
	Biagioni et al^21^, 2019	Brazil (2011-2016)	Other	27.2	91	91	67	44%	92%	67%
	Bosiers et al^22^, 2020 (Legflow)	Belgium and Germany (2015-2018)	DCB	21.6	120	120	71	66%	78%	30%
	Davaine et al^23^, 2012	France (2008-2009)	BMS	22	62	58	71	72%	83%	43%
			BMS	33.4	23	–	68	83%	–	–
	Dosluoglu et al^24^, 2008 (TASC D cohort)	United States, VA Western NY Healthcare System (2001-2007)	BMS	26.6	44	–	69	90%	80%	55%
	Giusca et al^25^, 2022	Germany (2017-2021)	Other	Not detailed for TASC D	108	108	77	62%	97%	51%
	Guo et al^26^, 2015	China (2011-2013)	BMS	31.5	58	53	74	68%	68%	60%
	Hacker and Marone^27^, 2018	United States (2003-2009)	Other	Not detailed	78	78	74	57%	78%	41%
	Han et al^28^, 2011	United States (1999-2008)	Other	Not detailed for TASC D	165	165	72	49%	86%	55%
	Iida et al^29^, 2022	Japan (2016-2017)	CS	Not detailed for TASC D	152	152	74	77%	84%	55%
	Kluckner et al^30^, 2022 (Pulsar-18 B)	Austria (2016-2018)	BMS	27.2	55	50	70	70%	88%	32%
	Labed et al^31^, 2021	France (2014-2017)	BMS	29.5	64	64	80	63%	72%	39%
	Lin et al^32^, 2019	Taiwan (2009-2014)	CS	22.1	55	55	73	69%	66%	78%
	Liu et al^33^, 2023	China (2016-2020)	BMS	28.6	57	54	68	76%	44%	28%
	Matsumi et al^34^, 2016	Japan (2004-2011)	BMS	24.5	72	68	73	71%	77%	52%
	Ohki et al^35^, 2017	Japan (prospective multicenter–15 hospitals; 2012-2013)	CS	21.8	60	60	74	83%	88%	60%
	Phillips et al^36^, 2018	United States (2012-2013)	DES	33	48	48	68	54%	85%	38%
	Uhl et al^37^, 2019	Germany (2010-2018)	CS	25.0 (median)	62	62	71	73%	57%	40%
	Zamani et al^38^, 2021	United States (2008-2018)	BMS	28	95	95	65	99%	88%	59%
			CS	26	74	74	64	100%	95%	45%
			DES	20	57	57	66	93%	93%	60%
	Zeller et al^39^, 2014	Belgium, Germany, and Austria (2011-2012)	CS	26.5	71	71	67	70%	79%	32%
RCT	Bosiers et al^40^, 2020 (Zilver PTX)	Belgium, Germany, Italy, and Brazil (2013-2017)	DES	24.2	113	113	70	69%	66%	27%
	Cheban et al^41^, 2023	Russia (2016-2019)	DES	26	30	30	63	83%	90%	49%
	Gabrielli et al^42^, 2012	Italy (2004-2008)	BMS	Not detailed	44	44	71% over 65	66%	75%	37%
	Kluckner et al^43^, 2022 (Pulsar-18 A)	Austria (2016-2020)	BMS	26.4	109	103	69	67%	86%	35%
	Lammer et al^44^, 2013	7 European centers (2009-2011, VIASTAR trial)	BMS	Not reported	16 TASC C, 22 TASC D	16 TASC C, 22 TASC D	69	75%	84%	36%
			CS	Not reported	18 TASC C, 34 TASC D	18 TASC C, 34 TASC D	69	67%	83%	35%
	Saaya et al^45^, 2022	Russia (2013-2017)	BMS	29	119	119	63	74%	66%	13%

BMS, bare metal stent; CS, covered stent; DCB, drug-coated balloon; DES, drug-eluting stent; DM, diabetes mellitus; HTN, hypertension; MLL, mean lesion length; RCT, randomized controlled trial; TASC, TransAtlantic Inter-Society Consensus; VIA, Viabahn endoprosthesis.

## Results

### Search results

**Figure 1:**
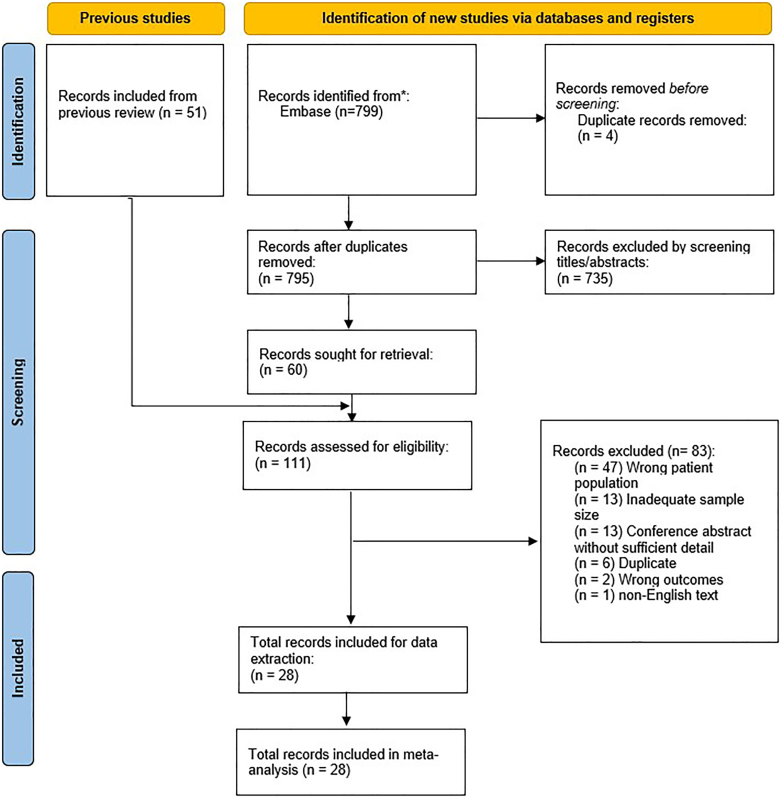
**PRISMA diagram, illustrating search strategy executed.****^7^** PRISMA, Preferred Reporting Items for Systematic Reviews and Meta-Analyses.

A total of 799 records were returned from database queries for the period 2019 to March 2023, with 795 records remaining after the removal of duplicates. Title/abstract screening led to the removal of 735 records, leaving 60 records for full-text review. Subsequently, the 51 records found relevant in the previously published search for period 2000 to June 2019 were included for full-text review, leading to a total of 111 records. During full-text review, studies were excluded due to inadequate sample size, wrong patient population (TASC mix or MLL), or conference proceedings without sufficient detail. After full-text review, 28 texts were included for data extraction. The full screening and review process is detailed in the PRISMA flow chart (Figure 1).^7^

### Included studies

The final analysis sample included 28 records, with a total of 2338 limbs and 2311 patients and mean age of 66.9 years, 66% male, and MLL of 29.3 cm.^18^, ^19^, ^20^, ^21^, ^22^, ^23^, ^24^, ^25^, ^26^, ^27^, ^28^, ^29^, ^30^, ^31^, ^32^, ^33^, ^34^, ^35^, ^36^, ^37^, ^38^, ^39^, ^40^, ^41^, ^42^, ^43^, ^44^, ^45^ More details on characteristics of included studies are shown in Table 1 and Supplementary Materials S3 and S4.

### Quality of evidence

Overall, according to GRADE criteria, the quality of evidence included in the analysis was moderate. Outcomes were primarily downgraded due to the presence of considerable heterogeneity and evidence of imprecision. The included studies were largely non-RCTs, with concerns of bias arising due to the potential for confounding, selection of participants, and measurement of outcomes. The risk of bias among the RCTs included was lower, with concern largely arising because of the measurement of the outcome, either due to selection of the comparator, single center, or small sample size. Qualitative and quantitative assessments confirmed the presence of publication bias, while the resulting summary statistics were indicative of moderate to substantial heterogeneity across the majority of the pooled analyses. Select subgroup analyses demonstrated improved heterogeneity. Supporting documentation of all quality appraisal assessments are shown in Supplementary Materials S5 to S7.

### Primary outcomes

The 12-month primary patency analysis included 30 observations and 2230 limbs, with cumulative pooled rates of 0.85 (95% CI, 0.81-0.89), 0.67 (95% CI, 0.61-0.73), 0.48 (95% CI, 0.38-0.57), and 0.42 (95% CI, 0.33-0.52) at 6, 12, 24, and 36 months, respectively. The pooled 12-month secondary patency analysis included 20 observations and 1446 limbs and resulted in a pooled cumulative secondary patency rate of 0.84 (95% CI, 0.77-0.89). The analysis of 12-month cumulative assisted primary patency included 14 observations and 1029 limbs, with a resulting pooled rate of 0.77 (95% CI, 0.69-0.83). The 12-month ffTLR pooled analysis included 17 observations and 1030 limbs, with a resulting cumulative pooled rate of 0.79 (95% CI, 0.74-0.83). The pooled 12-month mortality analysis included 12 observations and 876 patients, with a cumulative pooled rate of 0.08 (95% CI, 0.05-0.13). Lastly, the 12-month limb salvage analysis included 10 observations and 830 limbs, with a resulting pooled rate of 0.94 (95% CI, 0.89-0.98). For all outcomes, results from the meta-regression analysis suggest significant between-group differences (*P* < .001) relative to time. Full details of cumulative pooled event rates are shown in Table 2, with longitudinal pooled estimates presented in the Central Illustration.

**Table 2:** Cumulative pooled event rates.

	6 mo	12 mo	24 mo	36 mo
Primary patency	0.85	0.67	0.48	0.42
95% CI	0.81-0.89	0.61-0.73	0.38-0.57	0.33-0.52
*I*^2^	77.8%	83.7%	86.3%	81.2%
τ^2^	0.47	0.39	0.59	0.37
Prediction interval	0.58-0.96	0.36-0.88	0.15-0.83	0.16-0.74
Secondary patency	0.90	0.84	0.72	0.63
95% CI	0.85-0.93	0.77-0.89	0.64-0.78	0.52-0.73
*I*^2^	48.5%	75.8%	77.7%	85.3%
τ^2^	0.39	0.69	0.32	0.45
Prediction interval	0.68-0.97	0.46-0.97	0.42-0.90	0.27-0.89
ffTLR	0.93	0.79	0.68	0.63
95% CI	0.89-0.96	0.74-0.83	0.62-0.73	0.57-0.69
*I*^2^	27.0%	61.2%	52.2%	24.3%
τ^2^	0.43	0.19	0.06	0.0
Prediction interval	0.76-0.98	0.59-0.91	0.53-0.79	0.57-0.69
Assisted primary patency	0.85	0.77	0.61	0.57
95% CI	0.80-0.90	0.69-0.83	0.51, 0.71	0.42, 0.71
*I*^2^	62.0%	78.4%	84.9%	90.5%
τ^2^	0.24	0.40	0.43	0.75
Prediction interval	0.65-0.95	0.45-0.93	0.27-0.88	0.15-0.91
Mortality	0.04	0.08	0.18	0.23
95% CI	0.00-0.10	0.05-0.13	0.13-0.23	0.12-0.36
*I*^2^	80.0%	73.2%	68.9%	86.2%
τ^2^	0.01	0.0	0.0	0.02
Prediction interval	0.0-0.25	0.0-0.25	0.05-0.36	0.01-0.60
Limb salvage	0.96	0.94	0.90	0.84
95% CI	0.90-1.00	0.89-0.98	0.82-0.96	0.72-0.93
*I*^2^	84.4%	87.5%	89.7%	88.2%
τ^2^	0.01	0.02	0.02	0.03
Prediction interval	0.76-1.00	0.71-1.00	0.58-1.00	0.47-1.00

ffTLR, freedom from target lesion revascularization.

**Central Illustration:**
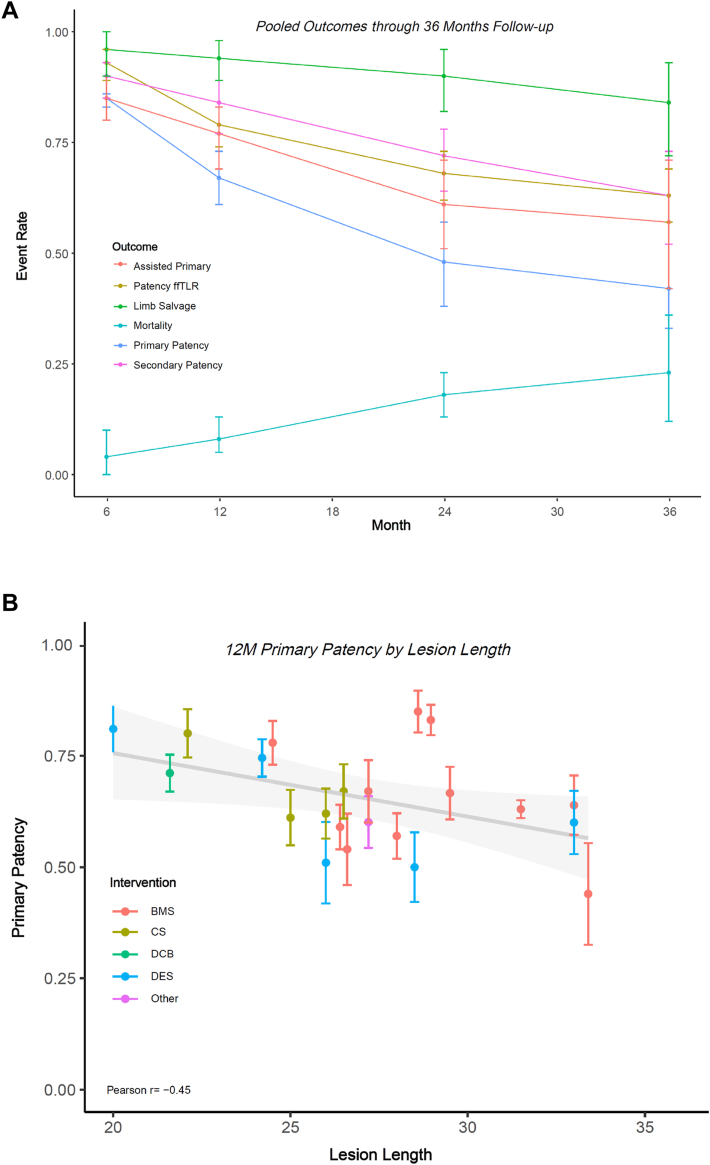
**(A) Longitudinal Pooled Estimates for all Outcomes. (B) Relationship between 12-month primary patency and MLL, stratified by intervention.** (A) Pooled cumulative event rates are denoted with point estimates with vertical lines indicating upper and lower bounds of the corresponding confidence intervals for assisted primary patency (red), ffTLR (gold), limb salvage (green), mortality (teal), primary patency (blue), and secondary patency (magenta). (B) study-observed 12-month cumulative primary patency rates are plotted by lesion length and stratified by intervention, including BMS (red), CS (gold), DCB (green), DES (blue), and Other (magenta). BMS, bare metal stents; CS, covered stents; DCB, drug-coated balloon; DES, drug-eluting stent; ffTLR, freedom from target lesion revascularization; MLL, mean lesion length.

### Sensitivity and subgroup analyses

The 12-month primary patency subgroup analysis by MLL found the pooled estimates for lesions 20.0 to 24.9 cm, 25.0 to 29.9 cm, and ≥30.0 cm to be as follows: 0.76 (95% CI, 0.69-0.81; *I*^2^ = 0%), 0.64 (95% CI, 0.58-0.71; *I*^2^ = 69.4%), and 0.60 (95% CI, 0.48-0.71; *I*^2^ = 4.1%), respectively. The inverse linear relationship observed between MLL and primary patency, stratified by endovascular intervention, demonstrated decreasing patency as MLL increased for studies reporting a cohort-specific MLL (Central Illustration). The 12-month ffTLR subgroup analysis by MLL found the pooled estimate for lesions 20.0 to 24.9 cm, 25.0 to 29.9 cm, and ≥30.0 cm to be as follows: 0.83 (95% CI, 0.75-0.89; *I*^2^ = 43.7%), 0.73 (95% CI, 0.66-0.78; *I*^2^ = 52.4%), and 0.82 (95% CI, 0.14-0.99; *I*^2^ = 0.0%), respectively. The 12-month secondary patency subgroup analysis by MLL found the pooled estimate for lesions 20.0 to 24.9 cm, 25.0 to 29.9 cm, and ≥30.0 cm to be as follows: 0.91 (95% CI, 0.83-0.96; *I*^2^ = 34.3%), 0.79 (95% CI, 0.69-0.88; *I*^2^ = 89.7%), and 0.85 (95% CI, 0.34-1.00; *I*^2^ = 85.6%). The 12-month mortality subgroup analysis by MLL found the pooled estimate for lesions 20.0 to 24.9 cm and 25.0 to 29.9 cm to be as follows: 0.08 (95% CI, 0.0-0.26; *I*^2^ = 66.4%) and 0.09 (95% CI, 0.06-0.12; *I*^2^ = 16.4%). Only 1 study reported data for MLL ≥30.0 cm.

Upon further stratification of 12-month outcomes by treatment modality, results were mixed in terms of performance; however, advanced modalities, such as covered and drug-eluting stents, were generally associated with improved outcomes. Directionally, all outcomes tended to decrease over time; however, the intervention-level assessment was hindered due to few studies consistently reporting at later time points; these results should be interpreted with caution. Similarly, stratification of texts by publication year, as a means to assess outcomes in the contemporary endovascular era, suggest minimal change for primary patency, ffTLR, mortality, and assisted primary patency. Secondary patency and limb salvage were significantly improved post-2018, relative to earlier time points.

The resulting pooled estimates for limb salvage, stratified by mixed (claudicant and CLTI) vs CLTI-only lesions were 0.97 (95% CI, 0.92-0.99; *I*^2^ = 83.9%) vs 0.80 (95% CI, 0.41-1.00; *I*^2^ = 0%), 0.92 (95% CI, 0.84-0.97; *I*^2^ = 89.9%) vs 0.76 (95% CI, 0.53-0.93; *I*^2^ = 0%), and 0.88 (95% CI, 0.74-0.97; *I*^2^ = 87.1%) vs 0.71 (95% CI, 0.62-0.79; *I*^2^ = 0%) at 12, 24, and 36 months, respectively. The between-group differences in pooled estimates were significant at all time points, but the CLTI estimates should be interpreted with caution, as only 3 studies (2 at these time points) were comprised solely of patients with CLTI. Additional subgroup analyses for all 12-month outcomes were explored, but only limb salvage was demonstrated to be significant.

**Figure 2:**
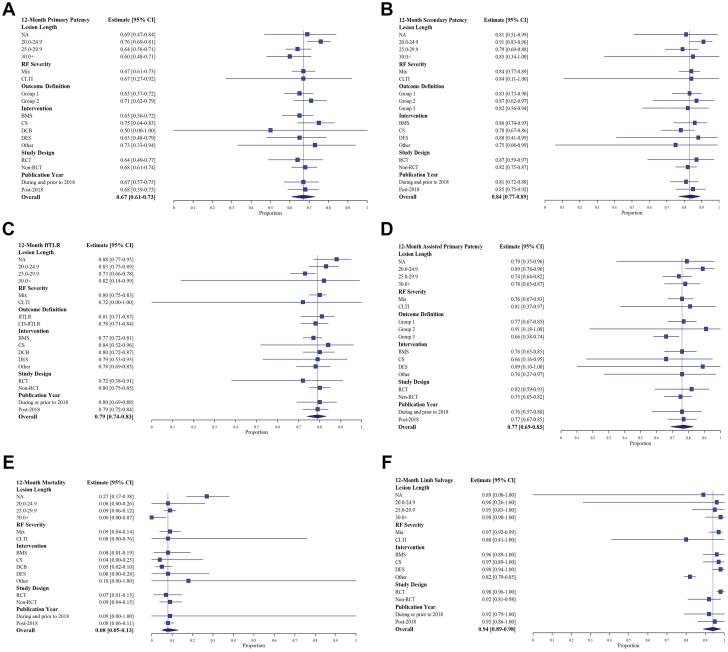
**12-month pooled outcomes for (A) primary patency, (B) secondary patency, (C) ffTLR, (D) assisted primary patency, (E) mortality, and (F) limb salvage.** In all panels, the resulting pooled estimates for subgroup analyses are denoted, with the overall pooled estimate denoted at the bottom and with the dashed vertical reference line. ffTLR, freedom from target lesion revascularization.

The majority of study-reported definitions were aligned with SVS standards (group 1); however, some demonstrated slight variation (group 2) or did not provide a definition (group 3) where appropriate. The 12-month primary patency subgroup analysis by definition found the pooled estimate for groups 1 and 2 to be as follows: 0.65 (95% CI, 0.57-0.72; *I*^2^ = 82.6%) and 0.71 (95% CI, 0.62-0.79; *I*^2^ = 84.8%). The 12-month secondary patency subgroup analysis by definition found the pooled estimate for groups 1, 2, and 3 to be as follows: 0.83 (95% CI, 0.73-0.90; *I*^2^ = 76.9%), 0.87 (95% CI, 0.62-0.97; *I*^2^ = 65.4%), and 0.82 (95% CI, 0.56-0.94; *I*^2^ = 82.5%). The difference between pooled clinically driven ffTLR and ffTLR estimates was again not significant, with pooled rates as follows: 0.78 (95% CI, 0.71-0.84; *I*^2^ = 65.3%) and 0.81 (95% CI, 0.71-0.87; *I*^2^ = 61.5%). The 12-month assisted primary patency subgroup analysis by definition found the pooled estimate for groups 1, 2, and 3 to be as follows: 0.77 (95% CI, 0.67-0.85; *I*^2^ = 78.6%), 0.91 (95% CI, 0.18-1.00; *I*^2^ = 0.0%), and 0.66 (95% CI, 0.58-0.74; *I*^2^ = 0.0%). See Figure 2 and Supplementary Material S8 for additional results.

## Discussion

This systematic search and meta-analysis provides a comprehensive body of evidence reporting on contemporary performance and outcomes associated with endovascular revascularization of long lesions in the femoropopliteal artery. Contemporary data, such as those reported in the present study, provide useful evidence for clinical decision making, given the evolution in endovascular technology and updated clinical guidelines that emphasize the role of endovascular revascularization in select patient groups.^3^, ^4^, ^5^, ^6^

Compared with the prior systematic search leveraged in the present study, more selective minimum sample size requirements and TASC mix criteria were applied, leading to only 12 records from the prior study being included in the current analysis.^19^^,^^23^^,^^26^, ^27^, ^28^^,^^34^, ^35^, ^36^^,^^39^^,^^41^^,^^42^^,^^44^ Despite these methodological differences, the resulting 6-, 12-, and 24-month pooled cumulative primary patency results were directionally comparable (76%, 62%, and 55% from Giannopoulos et al^8^ vs 85%, 67%, and 48% in the present analysis), as were 12- and 24-month target lesion revascularization rates (16% and 32% from Giannopoulos et al^8^ vs 21% and 32% in the present analysis).

Although the present study’s objective was not to compare endovascular treatment to surgical bypass or alternate revascularization strategies, the pooled estimates may still provide useful benchmark information. Prior analyses came to differing conclusions about the comparative outcomes of surgical versus endovascular treatment. Findings from a retrospective, observational study comparing endovascular (mean age 79 years, 81% CLTI) and above-the-knee surgical bypasses (mean age 74 years, 87.5% CLTI) performed with a prosthetic graft found 12-month treatment outcomes to be comparable in patients with complex TASC C/D lesions, while 12-, 24-, and 48-month primary patency, clinically driven target lesion revascularization, freedom from limb loss, and major adverse limb events were more favorable for the endovascular group.^49^ One of the strengths of the present analysis is the presentation of stratified results by treatment modality and lesion length, which can provide useful information for future studies further exploring the relative benefit and viability of endovascular versus surgical treatment in different types of cohort and lesion characteristics.

The analysis of contemporary data in the present study provides an opportunity for comparison to outcomes reported in historical meta-analyses of endovascular interventions. A systematic review including 23 cohort studies published from 1966 to 2007 reported a 12-month primary patency rate of 50% and limb salvage rates between 80% and 90% for subintimal angioplasty.^50^ Outcomes in the present analysis are more favorable and likely reflect continued progress and innovation in endovascular devices and procedural techniques, from stents to drug-coated balloons.^51^^,^^52^ Furthermore, the subgroup analysis including stratification of included studies by publication year provides a directional assessment of outcomes in the contemporary endovascular era, relative to historical evidence. However, these results should be interpreted with caution due to inconsistency in studies reporting across all time points and variation in the true period of data collection.

In the present study, several subgroup analyses were performed to report potential variation in pooled outcomes depending on the specific definition of outcomes. Variation in outcome reporting has previously been discussed as a shortcoming of studies in this clinical domain; however, results from this meta-analysis found variation between outcome definitions not to be significant, except for assisted primary patency.^17^ The definitions adopted for primary, secondary, and assisted primary patency were largely in agreement with SVS-based definitions, with variations largely stemming from patency determined based on hemodynamic rather than image-based measures.

This analysis is subject to several limitations. First, many of the included studies were nonrandomized trials, and observations included data representative of single-arm or single-center evaluations, which do contribute a degree of underlying bias and confounding. Second, the inclusion criteria specified long lesions, however additional studies with a mixed TASC C/D cohort were considered, as long as the reported MLL was ≥20 cm); therefore, while the final analysis sample had a MLL ≥20 cm., it was also inclusive of some TASC C lesions. Third, the pooled analyses may be impacted by selection bias, in terms of which patients may be preferred for endovascular treatment—and more specifically, the intervention chosen—as well as difference in outcomes expected for patients with CLTI compared to claudication. As a result, subgroup analyses excluding studies with full CLTI cohorts were explored. Fourth, some studies did not provide detailed cohort characteristics for subgroups of interest, and in some cases, inadequate stratification of results limited the studies included and overall breadth of the analysis explored. Where unavailable, overall cohort characteristics were assumed to reflect subgroups analyzed. In addition, standard error and population at risk were not consistently reported across studies. Ultimately, these structural search and study methodological limitations are evident in the pooled results, with the high degree of heterogeneity and overall moderate quality of evidence included in the review. Fifth, the analyses conducted were unadjusted for potential confounders, and further additional details such as the number of patent vessels, number of stents used, and length of stented lesion were not captured in the present analyses. Future studies will benefit from further exploring the effects of these potential confounders. Sixth, the assessment of between-group differences over time for all outcomes assumes independence of observations, which is inherently challenged by the notion that the same studies are reporting event rates over the time points evaluated, which may influence the standard error and *P* values observed. Finally, while most studies report data through 12 and 24 months, the evidence base gets smaller with longer follow-up, limiting statistical power for point estimates and subgroup analyses at the reported 36-, 48-, and 60-month time points; however, we believe understanding the strengths and limitations of available evidence to date is important to decision makers.

## Conclusion

This systematic search and meta-analysis provides important insights into the performance of endovascular revascularization in patients with long lesions. These findings need to be considered as treatment decisions for this patient population are explored.

## Declaration of competing interest

Sameh Sayfo serves as advisory/board member for Boston Scientific, Medtronic, Cagent, and Jupiter and reports speaker fees from Medtronic, Inari Medical, Penumbra, AngioDynamics, Surmodics, and Shockwave Medical. Jan B. Pietzsch, Anne M. Ryschon, and Abigail M. Garner, through their employer Wing Tech Inc, report consulting fees from Endologix. Ami Sood is an employee of Endologix. Peter A. Soukas reports consulting fees from Abbott, Boston Scientific, W.L. Gore and Associates, Endologix, Biotronik, Shockwave Medical, and Cordis; serves as an advisory/board member for Boston Scientific and Shockwave Medical; and has principal investigator roles for studies with W.L. Gore and Associates, Endologix, Contego Medical, Penumbra, REVA Medical, Reflow Medical, AVS, SoundBite, Bard, Shockwave Medical, InspireMD, and MicroMedical Solutions. Prakash Krishnan reports no disclosures. Peter A. Schneider reports consulting fees from Abbott, Philips, Boston Scientific, Medtronic, Endologix, Surmodics, Acotec, Cagent, Shockwave Medical, Inari, BD, and Healthcare Inroads.
