# Supplementary material for: Long Femoropopliteal Lesions Challenge the Limits of Endovascular Technique: Contemporary Systematic Review and Meta-analysis
Source: J Soc Cardiovasc Angiogr Interv. 2026 Feb 3;5(3):104158. doi: 10.1016/j.jscai.2025.104158 (PMC13005401; doi:10.1016/j.jscai.2025.104158)
Supplement: Supplementary Material [file mmc1.docx]

**SUPPLEMENTAL MATERIALS**

**Long Femoropopliteal Lesions Challenge the Limits of Endovascular Technique: Contemporary Systematic Review and Meta-analysis**

Sameh Sayfo, MD, MBA, FSCAI^a^*; Anne M. Ryschon, MA^b^*; Ami Sood, MD, FSCAI^c^; Abigail M. Garner, MS^b^; Peter A. Soukas, MD, FSCAI^d^; Prakash Krishnan, MD^e^; Jan B. Pietzsch, PhD^b^; Peter A. Schneider, MD^f^

^a^Department of Interventional Cardiology, Baylor Scott & White, The Heart Hospital – Plano, Plano, TX, USA; ^b^Wing Tech Inc., Menlo Park, CA, USA; ^c^Endologix, Campbell, CA, USA; ^d^Brown University Health Cardiovascular Institute, Providence, RI, USA; ^e^Zena and Michael A Wiener Cardiovascular Institute, Icahn School of Medicine at Mount Sinai, New York, NY, USA; ^f^Department of Surgery, University of California, San Francisco, San Francisco, CA, USA

**Joint first author*

Address for Correspondence:

Peter A. Schneider, MD

University of California, San Francisco, Department of Surgery

peteraschneidermd@gmail.com

**Table of Contents**

[**S1: PRISMA Checklist** 3](#_Toc217296777)

[**S2: Search Strategy** 9](#_Toc217296778)

[**S3: Outcome Definitions** 10](#_Toc217296779)

[**S4: Characteristics of Included Studies** 17](#_Toc217296780)

[**S5: Quality Appraisal: Risk of Bias Assessment** 30](#_Toc217296781)

[**S6: Quality Appraisal: Publication Bias** 32](#_Toc217296782)

[**S7: Summary of Findings and Quality of Evidence** 38](#_Toc217296783)

[**S8: Additional Results** 67](#_Toc217296784)

[**References** 113](#_Toc217296785)

# **S1: PRISMA Checklist**

The analysis was conducted in accordance with PRISMA standards for reporting, with detailed checklists completed below.^1^

Figure S1.1: Abstract Checklist

| **Section and Topic** | **Item #** | **Checklist item** | **Reported (Yes/No)** |
| --- | --- | --- | --- |
| **TITLE** | | |  |
| Title | 1 | Identify the report as a systematic review. | Yes |
| **BACKGROUND** | | |  |
| Objectives | 2 | Provide an explicit statement of the main objective(s) or question(s) the review addresses. | Yes |
| **METHODS** | | |  |
| Eligibility criteria | 3 | Specify the inclusion and exclusion criteria for the review. | Yes |
| Information sources | 4 | Specify the information sources (e.g. databases, registers) used to identify studies and the date when each was last searched. | Yes |
| Risk of bias | 5 | Specify the methods used to assess risk of bias in the included studies. | Yes |
| Synthesis of results | 6 | Specify the methods used to present and synthesise results. | Yes |
| **RESULTS** | | |  |
| Included studies | 7 | Give the total number of included studies and participants and summarise relevant characteristics of studies. | Yes |
| Synthesis of results | 8 | Present results for main outcomes, preferably indicating the number of included studies and participants for each. If meta-analysis was done, report the summary estimate and confidence/credible interval. If comparing groups, indicate the direction of the effect (i.e. which group is favoured). | Yes |
| **DISCUSSION** | | |  |
| Limitations of evidence | 9 | Provide a brief summary of the limitations of the evidence included in the review (e.g. study risk of bias, inconsistency and imprecision). | Yes |
| Interpretation | 10 | Provide a general interpretation of the results and important implications. | Yes |
| **OTHER** | | |  |
| Funding | 11 | Specify the primary source of funding for the review. | Yes |
| Registration | 12 | Provide the register name and registration number. | NA |

Figure S1.2: Main Manuscript Checklist

| **Section and Topic** | **Item #** | **Checklist item** | **Location where item is reported** |
| --- | --- | --- | --- |
| **TITLE** | | |  |
| Title | 1 | Identify the report as a systematic review. | Title |
| **ABSTRACT** | | |  |
| Abstract | 2 | See the PRISMA 2020 for Abstracts checklist. | See above. |
| **INTRODUCTION** | | |  |
| Rationale | 3 | Describe the rationale for the review in the context of existing knowledge. | Introduction |
| Objectives | 4 | Provide an explicit statement of the objective(s) or question(s) the review addresses. | Introduction |
| **METHODS** | | |  |
| Eligibility criteria | 5 | Specify the inclusion and exclusion criteria for the review and how studies were grouped for the syntheses. | Methods, Selection Criteria |
| Information sources | 6 | Specify all databases, registers, websites, organisations, reference lists and other sources searched or consulted to identify studies. Specify the date when each source was last searched or consulted. | Methods, Search Strategy |
| Search strategy | 7 | Present the full search strategies for all databases, registers and websites, including any filters and limits used. | Methods, Search Strategy |
| Selection process | 8 | Specify the methods used to decide whether a study met the inclusion criteria of the review, including how many reviewers screened each record and each report retrieved, whether they worked independently, and if applicable, details of automation tools used in the process. | Methods, Selection of Relevant Records |
| Data collection process | 9 | Specify the methods used to collect data from reports, including how many reviewers collected data from each report, whether they worked independently, any processes for obtaining or confirming data from study investigators, and if applicable, details of automation tools used in the process. | Methods, Selection of Relevant Records |
| Data items | 10a | List and define all outcomes for which data were sought. Specify whether all results that were compatible with each outcome domain in each study were sought (e.g. for all measures, time points, analyses), and if not, the methods used to decide which results to collect. | Methods, Selection of Relevant Records |
|  | 10b | List and define all other variables for which data were sought (e.g. participant and intervention characteristics, funding sources). Describe any assumptions made about any missing or unclear information. | Methods, Selection of Relevant Records |
| Study risk of bias assessment | 11 | Specify the methods used to assess risk of bias in the included studies, including details of the tool(s) used, how many reviewers assessed each study and whether they worked independently, and if applicable, details of automation tools used in the process. | Methods, Quality Appraisal Assessments |
| Effect measures | 12 | Specify for each outcome the effect measure(s) (e.g. risk ratio, mean difference) used in the synthesis or presentation of results. | Methods, Statistical Analyses |
| Synthesis methods | 13a | Describe the processes used to decide which studies were eligible for each synthesis (e.g. tabulating the study intervention characteristics and comparing against the planned groups for each synthesis (item #5)). | Methods, Statistical Analyses |
|  | 13b | Describe any methods required to prepare the data for presentation or synthesis, such as handling of missing summary statistics, or data conversions. | Methods, Statistical Analyses |
|  | 13c | Describe any methods used to tabulate or visually display results of individual studies and syntheses. | Methods, Statistical Analyses |
|  | 13d | Describe any methods used to synthesize results and provide a rationale for the choice(s). If meta-analysis was performed, describe the model(s), method(s) to identify the presence and extent of statistical heterogeneity, and software package(s) used. | Methods, Statistical Analyses |
|  | 13e | Describe any methods used to explore possible causes of heterogeneity among study results (e.g. subgroup analysis, meta-regression). | Methods, Statistical Analyses; Quality Appraisal Assessments |
|  | 13f | Describe any sensitivity analyses conducted to assess robustness of the synthesized results. | Methods, Statistical Analyses |
| Reporting bias assessment | 14 | Describe any methods used to assess risk of bias due to missing results in a synthesis (arising from reporting biases). | Methods, Quality Appraisal Assessments |
| Certainty assessment | 15 | Describe any methods used to assess certainty (or confidence) in the body of evidence for an outcome. | Methods, Quality Appraisal Assessments |
| **RESULTS** | | |  |
| Study selection | 16a | Describe the results of the search and selection process, from the number of records identified in the search to the number of studies included in the review, ideally using a flow diagram. | Results, Included Studies |
|  | 16b | Cite studies that might appear to meet the inclusion criteria, but which were excluded, and explain why they were excluded. | Results, Excluded Studies |
| Study characteristics | 17 | Cite each included study and present its characteristics. | Results, Included Studies, Table 1, Appendix |
| Risk of bias in studies | 18 | Present assessments of risk of bias for each included study. | Appendix |
| Results of individual studies | 19 | For all outcomes, present, for each study: (a) summary statistics for each group (where appropriate) and (b) an effect estimate and its precision (e.g. confidence/credible interval), ideally using structured tables or plots. | Results, Primary Outcomes, supporting figures and Appendix |
| Results of syntheses | 20a | For each synthesis, briefly summarise the characteristics and risk of bias among contributing studies. | Appendix, Summary of Findings tables |
|  | 20b | Present results of all statistical syntheses conducted. If meta-analysis was done, present for each the summary estimate and its precision (e.g. confidence/credible interval) and measures of statistical heterogeneity. If comparing groups, describe the direction of the effect. | Results, Primary Outcomes, supporting figures and Appendix |
|  | 20c | Present results of all investigations of possible causes of heterogeneity among study results. | Results, Sensitivity and Subgroup Analyses, Appendix |
|  | 20d | Present results of all sensitivity analyses conducted to assess the robustness of the synthesized results. | Results, Sensitivity and Subgroup Analyses, Appendix |
| Reporting biases | 21 | Present assessments of risk of bias due to missing results (arising from reporting biases) for each synthesis assessed. | Appendix, Summary of Findings tables |
| Certainty of evidence | 22 | Present assessments of certainty (or confidence) in the body of evidence for each outcome assessed. | Appendix, Summary of Findings tables |
| **DISCUSSION** | | |  |
| Discussion | 23a | Provide a general interpretation of the results in the context of other evidence. | Discussion |
|  | 23b | Discuss any limitations of the evidence included in the review. | Discussion |
|  | 23c | Discuss any limitations of the review processes used. | Discussion |
|  | 23d | Discuss implications of the results for practice, policy, and future research. | Discussion |
| **OTHER INFORMATION** | | |  |
| Registration and protocol | 24a | Provide registration information for the review, including register name and registration number, or state that the review was not registered. | NA |
|  | 24b | Indicate where the review protocol can be accessed, or state that a protocol was not prepared. | NA |
|  | 24c | Describe and explain any amendments to information provided at registration or in the protocol. | NA |
| Support | 25 | Describe sources of financial or non-financial support for the review, and the role of the funders or sponsors in the review. | Declared in main manuscript. |
| Competing interests | 26 | Declare any competing interests of review authors. | Declared in main manuscript. |
| Availability of data, code and other materials | 27 | Report which of the following are publicly available and where they can be found: template data collection forms; data extracted from included studies; data used for all analyses; analytic code; any other materials used in the review. | Reference to supporting material are made in main paper. |

# **S2: Search Strategy**

Table S2.1: Primary search strategy executed.^2^

|  | Term | Results |
| --- | --- | --- |
| 1 | ‘subsartorial artery’ | 4,670 |
| 2 | superficial AND femoral AND artery | 7,220 |
| 3 | femoropopliteal | 8,167 |
| 4 | #1 OR #2 OR #3 | 15,170 |
| 5 | Long | 2,696,233 |
| 6 | Tasc | 2,169 |
| 7 | #5 OR #6 | 2,697,803 |
| 8 | #4 OR #7 | 3,567 |
| 9 | #8 AND (2019:py OR 2020:py OR 2021:py OR 2022:py OR 2023:py) | 798 |

# **S3: Outcome Definitions**

Table S3.1-4: (1) Primary Patency Definitions, (2) Secondary Patency Definitions, (3) Assisted Primary Patency Definitions, and (4) ffTLR Definitions.

| Study | Primary Patency Definition |  |
| --- | --- | --- |
| AbuRahma et al., 2019 (3) | Primary patency was defined as the absence of >50% restenosis or occlusion in the treated arterial segment according to DUS or angiography. This was defined by a decrease of the ABI by >0.15 or evidence of stenosis by DUS of PSV>300cm or a ratio >3. |  |
| AbuRahma et al., 2022 (4) | Primary patency: defined as absence of ≥50% restenosis or occlusion of the treated arterial segment according to DUS/angiography |  |
| Astarcloglu et al., 2017 (5) | Primary patency was defined as no clinically driven reintervention and freedom > 50% restenosis within the treated segment. |  |
| Biagioni et al., 2019 (6) | Primary patency was defined as the interval elapsed without the need for additional or secondary surgical or endovascular procedures and the identification of a >70%stenosis or occlusion of the treated segment of the SFA and popliteal artery. |  |
| Bosiers et al., 2020a (7) | Primary patency was defined as no evidence of binary restenosis or occlusion within the target lesion or bypass graft based on a duplex-derived peak systolic velocity ratio. For the ZILVER PTX arm, this was defined as no evidence of binary restenosis or occlusion within the target lesion based on a duplex-derived peak systolic velocity ratio (PSVR) <2.4 and no clinically-driven target lesion revascularization (TLR) |  |
| Bosiers et al., 2020b (8) | Primary patency was defined as absence of a hemodynamically significant stenosis on duplex ultrasound (systolic velocity ratio no greater than 2.4) at the target lesion and without TLR within 12 months |  |
| Cheban et al., 2023 (9) | Primary patency was defined as the absence of an occlusion or flow-limiting stenosis of the treated segment including 1cm of the proximal and distal zones of the stent; restenosis >70% |  |
| Davaine et al., 2012 (10) | Primary patency was defined as patency without any percutaneous or surgical intervention in the treated segment or in the adjacent areas. |  |
| Dosluoglu et al., 2008 (11) | A patent runoff vessel was defined as an infrapopliteal vessel without a hemodynamically significant (50%) angiographic steno sis distal to the treated site, and the number of adequately patent runoff vessels (0 to 3) was calculated after all interventions were completed for that limb. |  |
| Gabrielli et al., 2012 (12) | Primary patency was defined as the absence of restenosis in the treated arterial segment. |  |
|  |  |  |
| Guo et al., 2015 (13) | Primary patency was defined as the percentage of patients without any re-stenosis or occlusion in the arterial segment undergoing intervention during the follow-up period. |  |
| Hacker et al., 2018 (14) | Primary patency was defined as the absence of restenosis or occlusion in the treated arterial segment |  |
| Han et al., 2011 (15) | Patency was first determined by arterial duplex of the treated vessel and then by ABIs and clinical evaluation. Loss of patency on arterial duplex was defined as an occlusion or a reduction of the arterial lumen diameter by >50%, with a velocity ratio greater than 2.5:1. A decrease of >0.15 in the ABI or a CT angiography demonstrating vessel reocclusion was also considered as a loss of patency. At follow-up, a patient was considered to have a loss of patency if restenosis or occlusion was detected in any of the lesions treated. |  |
| Iida et al., 2022 (16) | Patients with stenosis or occlusion of the target lesion with peak systolic velocity ratio (PSVR) ≥ 2.5 (or no PSVR measurement) were considered to have lost primary patency. |  |
| Kluckner et al., 2022 (17) | The absence of flow-limiting stenosis (peak systolic velocity (PSV) ratio > 2.5) or occlusion of the treated artery was defined as primary patency |  |
| Labed et al., 2021 (18) | Primary patency was defined as the absence of significant intrastent restenosis or occlusion confirmed by ultrasound imaging or angio-CT. The primary outcome was the 12-month primary patency, defined by the absence of restenosis (≥50%) and/or reintervention on the target lesion |  |
| Lammer et al., 2013 (19) | Primary patency was defined as no evidence of restenosis 50% or occlusion within the study lesion based on CDUS with a peak systolic velocity ratio 2.5 and no target lesion revascularization (TLR) within 12 months |  |
|  |  |  |
| Lin et al., 2019 (20) | Loss of primary patency was defined as CTA results or angiographic evidence of restenosis greater than 50% or occlusion and target lesion revascularization (TLR). |  |
|  |  |  |
| Liu et al., 2023 (21) | Persistent patency without any other revascularization in the treated arterial segment |  |
| Matsumi et al., 2016 (22) | Primary patency was defined as a treated vessel without restenosis or revascularization. Restenosis was defined as a peak systolic velocity rate of >2.5 in a duplex ultrasound examination or of >50% stenosis in an angiogram. |  |
| Ohki et al., 2017 (23) | Primary patency-interventional was defined as peak systolic velocity ratio <2.5 without TLR in treated lesions |  |
| Phillips et al., 2018 (24) | Not explicitly defined, but reports restonosis >50% |  |
| Saaya et al., 2022 (25) | Primary patency was defined as exempt from restenosis/reocclusion of the target lesion during follow-up. Restenosis defined as more than 70% stenosis on duplex imaging and CTA |  |
| Uhl et al., 2019 (26) | The definition of the patency rates followed Rutherford et al. (assume restonosis >50%) |  |
| Zamani et al., 2021 (27) | Patency was defined based on SVS reporting standards. |  |
| Zeller et al., 2014 (28) | Primary patency was defined as no restenosis or occlusion within the treated lesion. Restonosis was based on duplex ultrasound measurement >2.5 PSV |  |

| Study | Secondary Patency Definition |
| --- | --- |
| Astarcloglu et al., 2017 (5) | Secondary patency was defined as freedom from > 50% restenosis. |
| Biagioni et al., 2019 (6) | Secondary patency was achieved by performing reintervention on occluded, previously treated arterial segments. |
| Cheban et al., 2023 (9) | Secondary patency reports patency of the target lesion after treatment of a reocclusion. |
| Gabrielli et al., 2012 (12) | Secondary patency was achieved with additional procedures to treat the reocclusion. |
| Guo et al., 2015 (13) | Secondary patency rate was defined as the percentage of patients without re-stenosis or occlusion and patients achieved patency utilizing additional endo vascular interventions in the occluded arterial segments |
| Hacker et al., 2018 (14) | Secondary patency was achieved utilizing additional endovascular procedures, which involved recanalizing occluded arterial segments |
| Iida et al., 2022 (16) | Secondary patency was defined as absence of surgical bypass and hemodynamic evidence of blood flow through the device; all those with device removal,  bypass, or occlusion without successful revision were considered to have lost secondary patency. |
| Kluckner et al., 2022 (17) | Secondary patency was determined as a secondary intervention performed for stent occlusion in a subsequently patent vessel. |
| Lin et al., 2019 (20) | Loss of secondary patency was the first occurrence of angiography demonstrating total occlusion with failure of interventional revascularization. |
| Liu et al., 2023 (21) | Secondary patency was defined as the duration of patent revascularization after new intervention for occlusion |
| Matsumi et al., 2016 (22) | Secondary patency was defined as patency after TLR to the restenosis or occlusion in a treated vessel. |
| Saaya et al., 2022 EI (25) | Secondary patency reports patency of the target lesion after treatment of a reocclusion of the index lesion |
| Uhl et al., 2019 (26) | The definition of the patency rates followed Rutherford et al. |
| Zamani et al., 2021 (27) | Patency and TLR were defined in accordance with the Society for Vascular Surgery reporting standards |
| Zeller et al., 2014 (28) | Secondary patency referred to restoration of patency by TLR after restenosis/occlusion. |

| Study | Assisted Primary Patency Definition |  |
| --- | --- | --- |
| AbuRahma et al., 2019 (3) | Assisted primary patency was defined as restored patency after endovascular reintervention (PTA or stenting) for restenotic lesions. Limbs that underwent vascular bypass after occlusion of the treated segment were not considered patent. |  |
| Biagioni et al., 2019 (6) | Assisted primary patency indicated patency of the endovascular intervention achieved with the use of additional or secondary surgical or endovascular procedures, as long as occlusion of the primary treated site had not occurred. |  |
| Cheban et al., 2023 (9) | Primary-assisted patency was defined as patency of the target lesion following reintervention at the target vessel site in case of symptomatic restenosis. |  |
| Gabrielli et al., 2012 (12) | Assisted primary patency was achieved by secondary endovascular interventions to treat restenoses involving the originally treated arterial segment |  |
|  |  |  |
| Guo et al., 2015 (13) | Assisted-primary patency rate was defined as the percentage of patients without re-stenosis or occlusion and patients who achieved patency via additional endovascular interventions in the arterial segments suffering re-stenosis. |  |
| Hacker et al., 2018 (14) | Assisted-primary patency was achieved via secondary endovascular interventions to treat restenosis involving the originally treated arterial segment. Additional procedures to treat lesions proximal or distal to the initially treated segment were also considered secondary interventions to achieve assisted-primary patency |  |
| Iida et al., 2022 (16) | Primary-assisted patency was defined as hemodynamic evidence of blood flow through the device that did not require target lesion revascularization (TLR) to restore flow after total occlusion |  |
| Liu et al., 2023 (21) | Persistent patency through new EVT in stenotic segment |  |
| Ohki et al., 2017 (23) | Assisted Primary Patency was defined as a stent graft that had not occluded at any time (although target lesion revascularization [TLR] could have been performed for stenosis). |  |
| Saaya et al., 2022 (25) | Primary-assisted patency was defined as patency of the target lesion after endovascular reintervention at the target vessel site in case of symptomatic restenosis. |  |
|  |  |  |
| Uhl et al., 2019 (26) | The definition of the patency rates followed Rutherford et al. |  |
| Zamani et al., 2021 (27) | Patency and TLR were defined in accordance with the Society for Vascular Surgery reporting standards |  |
|  |  |  |
|  |  |  |

| Study | ffTLR Definition |  |
| --- | --- | --- |
| AbuRahma et al., 2019 (3) | Target lesion revascularization was done for recurrent stenosis of >50% that was associated with recurrence of symptoms. |  |
| Bosiers et al., 2020b (8) | TLR was defined as a repeat intervention to maintain or re-establish patency within the region of the treated arterial vessel plus 5 mm proximal and distal to the treated lesion edge at the respective time points; clinical success at follow-up, defined as an improvement of Rutherford classification at all follow-up time points of one class or more as compared to the pre-procedure Rutherford classification. |  |
| Cheban et al., 2023 (9) | TLR was defined as are intervention at the index lesion. Target lesion revascularization (TLR) was defined as a reintervention at the index lesion. |  |
| Davaine et al., 2012 (10) | TLR expresses the frequency of the need for repeated procedures (endovascular or surgical) due to a problem arising from the lesion in surviving patients with preserved limb. |  |
| Giusca et al., 2022 (29) | CD-TLR was assessed |  |
| Labed et al., 2021 (18) | TLR was expressed as a percentage and defined by the absence of repeat percutaneous intervention of the target lesion or bypass surgery of the target vessel performed for restenosis or occlusion of the target lesion previously treated (with 10 mm proximal and distal a margin) in patients with a preserved limb. |  |
| Liu et al., 2023 (21) | Revascularization of a treated arterial segment, performed on a patient who returned due to symptomatic recurrence and restenosis or occlusion as determined by duplex ultrasound, following EVT of femoropopliteal PAD |  |
| Matsumi et al., 2016 (22) | Target-lesion revascularization (TLR) was defined as repeated vascularization of the stented segment within 5 mm proximal or distal to the stent. Clinically driven TLR was defined as TLR for patients with ischemic symptoms in a lower limb |  |
| Phillips et al., 2018 (24) | CD-TLR |  |
| Saaya et al., 2022 (25) | A reintervention at the index lesion site was defined as target lesion revascularization |  |
|  |  |  |
| Zamani et al., 2021 (27) | TLR was defined by the first endovascular or open reintervention on the target lesion, including thrombectomy or bypass revision |  |
|  |  |  |
|  |  |  |

# **S4: Characteristics of Included Studies**

Table S4.1: Full detailing of characteristics of Included Studies.

| **Study** | **Intervention** | **Mean LL (cm)** | **N (limbs)** | **N (patients)** | **Age** | **% Male** | **HTN** | **DM** | **Smoking** | **Claudicants, CLTI** | **Funding** | **Technical Success %** |
| --- | --- | --- | --- | --- | --- | --- | --- | --- | --- | --- | --- | --- |
| **AbuRahma et al., 2019, TASC D (3)** | *DCB (Lutonix 0.35; Bard, Tempe, Arizona)* | *Not reported.* | *81* | *81* | *68* | *46%* | 72% | 53% | 27% | 40%, 60% | No funding to disclose | 98% |
| **AbuRahma et al., 2022, TASC D (4)** | *DES (Zilver PTX)* | *28.5 cm* | *47* | 47 | *69* | *57%* | 57% | 43% | 30% | 32%, 68% | No funding to disclose | 99% |
| **Astarcloglu et al., 2017, TASC D (5)** | *Self-expanding Nitinol Stent* | *33.0 (median)* | *52* | 48 | *69* | *79%* | 77% | 48% | 63% | 58%, 42% | No funding to disclose | 91% |
| **Biagioni et al., 2019, TASC D (6)** | *Mix, mostly Nitinol Stent* | *27.2* | *91* | 91 | *67* | *44%* | 92% | 67% | 22% | 0%, 100% | No funding to disclose | 97% |
| **Bosiers et al., 2020a (Zilver PTX), TASC C/D (7)** | *DES (Zilver PTX)* | *24.2* | *113* | 113 | *70* | *69%* | 66% | 27% | 69% | 71%, 29% | No funding to disclose | 100% |
| **Bosiers et al., 2020b (Legflow), TASC C/D (8)** | *DCB* | *21.6* | *120* | 120 | *71* | *66%* | 78% | 30% | 57% | 78%, 22% | Not detailed. | 100% |
| **Cheban et al., 2023, TASC D (9)** | *DES (Zilver, only control considered)* | *26.0* | *30* | 30 | *63* | *83%* | 90% | 49% | 87% | 0%, 100% | Sponsored by Cook Medical | Not reported. |
| **Davaine et al., 2012, (10)** | *Nitinol Stent (LifeStent), TASC C/D* | *22.0 (median)* | *62* | *58* | *71* | *72%* | 83% | 43% | 59% | 40%, 60% | None to report | Not reported |
|  | *Nitinol Stent (LifeStent), TASC D* | *33.4* | *23* |  | *68* | *83%* |  |  |  | 30%, 70% |  |  |
| **Dosluoglu et al., 2008 TASC D (11)** | *Percutaneous balloon angioplasty/stenting* | *26.6* | *44* | 44 | *69* |  | 80% | 55% | 39% | 27%, 61% | None to report | 84% |
| **Gabrielli et al., 2012, TASC D (12)** | *Mix (angioplasty w stenting)* |  | *44* | 44 | *71% over 65* | *66%* | 75% | 37% | 76% | 48%, 52% | None disclosed | 93% |
| **Giusca et al., 2022, TASC D (29)** | *Phoenix Atherectomy* | *Not reported.* | *108* | 108 | *77* | *62%* | 97% | 51% | 41% | 34%, 66% | GRN Hospital Weinheim (G. Korosoglou & S. Giusca) received an institutional research grant from Volcano and Philips. | 60% (procedural success 99%, defined separately) |
| **Guo et al., 2015, TASC D (13)** | *Nitinol Stent (Protégé  Everflex (ev3 Inc., USA), Lifestent (Bard Inc, USA), and  SMART Control (Cordis Corporation))* | *31.5* | *58* | 53 | *74* | *68%* | 68% | 60% | NR | 52%, 48% | This study was supported by Shanghai committee of science and technology key scientific research project (NO. 11441900602). | 95% |
| **Hacker et al., 2018, TASC D (14)** | *Mix (balloon angioplasty and/or stenting)* |  | *78* | 78 | *74* | *57%* | 78% | 41% | 64% | 55%, 42% | Not detailed. | Defined but not reported |
| **Han et al., 2011, TASC D (15)** | *Mix* | *Not reported.* | *165* | 165 | *72* | *49%* | 86% | 55% | 57% | 49%, 52% | Not reported | Not reported |
| **Iida et al., 2022, TASC D (16)** | *VIA* | *Not reported.* | *152* | 152 | *74* | *77%* | 84% | 55% | 50% former; 26% current | 70%, 27% | Not reported | 97% |
| **Kluckner et al., 2022 (Pulsar-18 A, Long-term...), TASC C/D (30)** | *BMS* | *26.4* | *109* | 103 | *69* | *67%* | 86% | 35% | 35% | 47%, 53% | None to report | 88% |
| **Kluckner et al., 2022 (Pulsar-18 B, Comparison...), TASC C/D (17)** | *BMS* | *27.2* | *55* | 50 | *70* | 70% | 88% | 32% | 30% | 51%, 49% | None to report | 87% |
| **Labed et al., 2021, TASC C/D (18)** | *BMS (Absolute pro, Abbott Vascular Inc., Chicago, IL, USA), Everflex (Ev3 endovascular Inc.), Chicago, IL, USA, Lifestent (Bard Peripheral Vascular), Innova* | *29.5* | *64* | 64 | *80* | *63%* | 72% | 39% | 45% | 23%, 77% | Not detailed. | Not reported |
| **Lammer et al., 2013, TASC D (19)** | *BMS, VIA (the Viabahn endoprosthesis (WL Gore, Flag staff, Arizona) with the contoured proximal edge and  heparin-bonded surface (PROPATEN Bioactive Surface)  was used. The BMS used were the Life-Stent (BARD  Peripheral Vascular, Inc., Tempe, Arizona), the Protégé  EverFlex Stent (ev3 Inc., Plymouth, Minnesota), and the  SMART-Control Stent (Cordis Corporation, Johnson &  Johnson, Warren, Massachusetts))* | *Not reported* | *16 TASC C, 22 TASC D (BMS)* | *16 TASC C, 22 TASC D (BMS)* | *69 (BMS)* | *75% BMS* | 84% BMS | 36% BMS | 70% BMS | 82%, 18% BMS | None to report | 99% |
|  |  |  | *18 TASC C, 34 TASC D (VIA)* | *18 TASC C, 34 TASC D (VIA)* | *69 VIA* | *67% VIA,* | 83% VIA | 35% VIA | 69% VIA | 86%, 14% VIA |  |  |
| **Lin et al., 2019, TASC C/D (20)** | *VIA* | *22.1* | *55* | 55 | *73* | *69%* | 66% | 78% | 66% | 15%, 85% | None to report | 100% |
| **Liu et al., 2023, TASC D (21)** | *Nitinol Stent* | *28.6* | *57* | 54 | *68* | *76%* | 44% | 28% | 26% current; 46% former | 0%, 100% | The study was a government-sponsored program and funded by the Medical Science Research Project of the Key Research and Development Program of Hebei Province, China (No: 20377732D). | 98% |
| **Matsumi et al., 2016, TASC D (22)** | *Nitinol Stent* | *24.5* | *72* | 68 | *73* | *71%* | 77% | 52% | 66% (former) | 79%, 21% | Not detailed. | Not reported |
| **Ohki et al., 2017, TASC D (23)** | *VIA* | *Not reported.* | *60* | 60 | *74* | *83%* | 88% | 60% | 79 (current or former) | 97%, 3% | Potential funding, not explicitly detailed | 99% |
| **Phillips et al., 2018, TASC D (24)** | *DES (Zilver PTX)* | *33.0* | *48* | 48 | *68* | *54%* | 85% | 38% | 54% | 65%, 35% | Not detailed | 100% |
| **Saaya et al., 2022, TASC D (25)** | *Mix* | *29.0* | *119* | 119 | *63* | *74%* | 66% | 13% | 49% | 21%, 79% | Not detailed | 96% |
| **Uhl et al., 2019, TASC C/D (26)** | *VIA* | *25.0 (median)* | *62* | 62 | *71* | *73%* | 57% | 40% | 34% | 61%, 39% | Not detailed | Not detailed |
| **Zamani et al., 2021, TASC C/D (27)** | BMS used throughout the study period included the LifeStent Vascular Stent System (Bard Peripheral Vascular, Inc., Tempe, AZ), EverFlex and Protege EverFlex (Medtronic, Minneapolis, MN), and the Complete SE Vascular Stent System (Medtronic). | *28.0* | *95* | *95* | *65* | *99%* | 88% | 59% | 47% current; 44% former | 61%, 39% | Not applicable | Not reported |
|  | CS was self-expanding nitinol stents covered on their luminal surface with heparin-bonded, expanded polytetrafluoroethylene (GORE VIABAHN Endoprosthesis; W. L. Gore and Associates, Inc., Flagstaff, AZ) | *26.0* | *74* | *74* | *64* | *100%* | 95% | 45% | 57% current; 34% former | 69%, 31% |  |  |
|  | DES was self-expanding nitinol stents with a polymer-free paclitaxel coating (Zilver PTX; Cook Medical, Blooming ton, IN | *20.0* | *57* | *57* | *66* | *93%* | 93% | 60% | 44% current; 28% former | 33%, 67% |  |  |
| **Zeller et al., 2014, TASC C/D (28)** | *VIA* | *26.5* | *71* | 71 | *67* | *70%* | 79% | 32% | 52% current; 30% former | 91%, 9% | W.L. Gore & Associates, Flagstaff AZ | 76% |

BMS: Bare metal stents; CS: Covered stents; DCB: drug coated balloon; DES: drug eluting stent; RCT: Randomized-controlled trial; VIA: Viabahn endoprosthesis

# **S5: Quality Appraisal: Risk of Bias Assessment**

The following figures provide a summary of risk of bias assigned, by domain of bias evaluated, as well as the corresponding judgements assigned at the study level.^31,32^

Figure S5.1-2: (1) Summary Judgements for included RCTs, (2) Supporting judgement assigned at the study level for included RCTs.


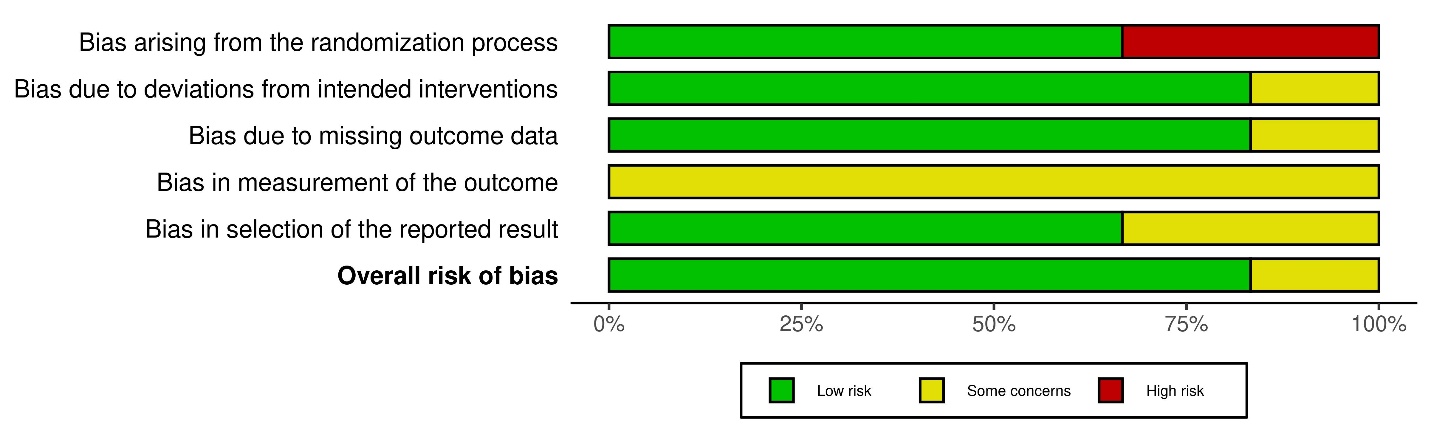


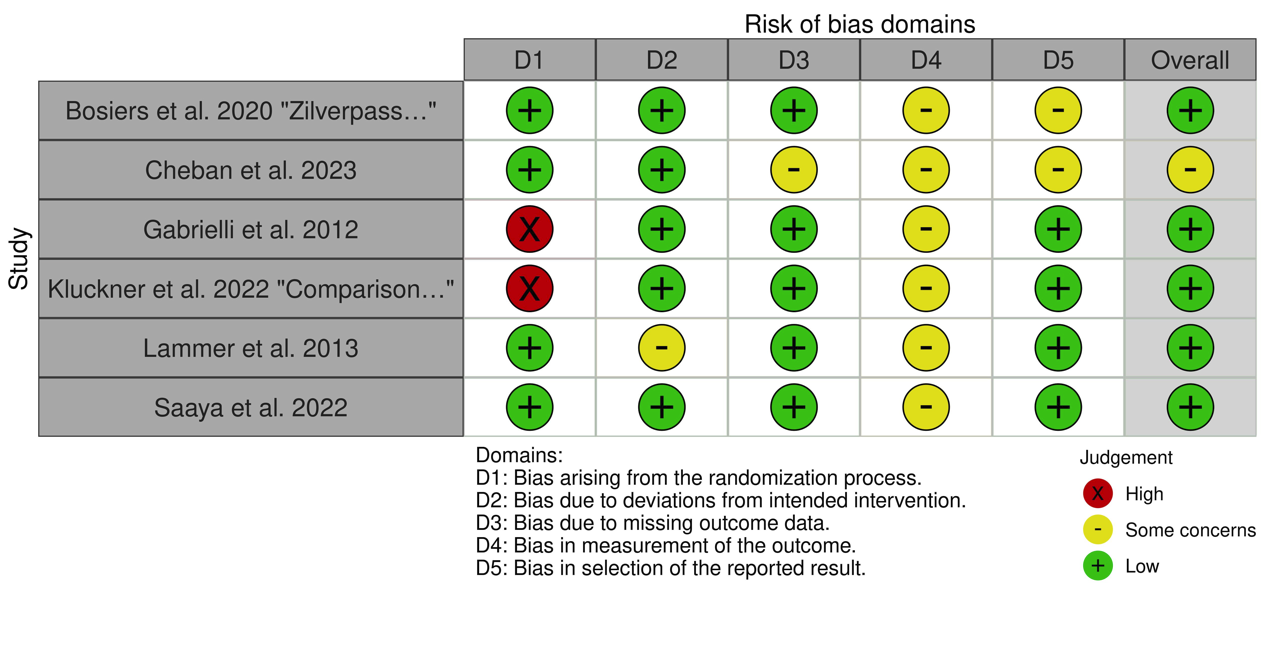


Figure S5.3-4: (3) Summary Judgements for included non-RCTs, (4) Supporting judgement assigned at the study level for included non-RCTs.


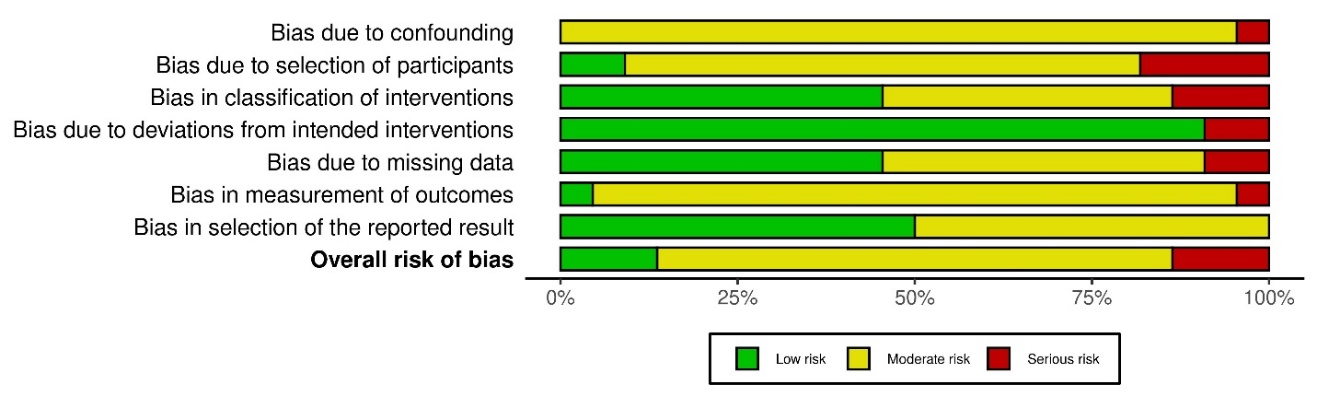


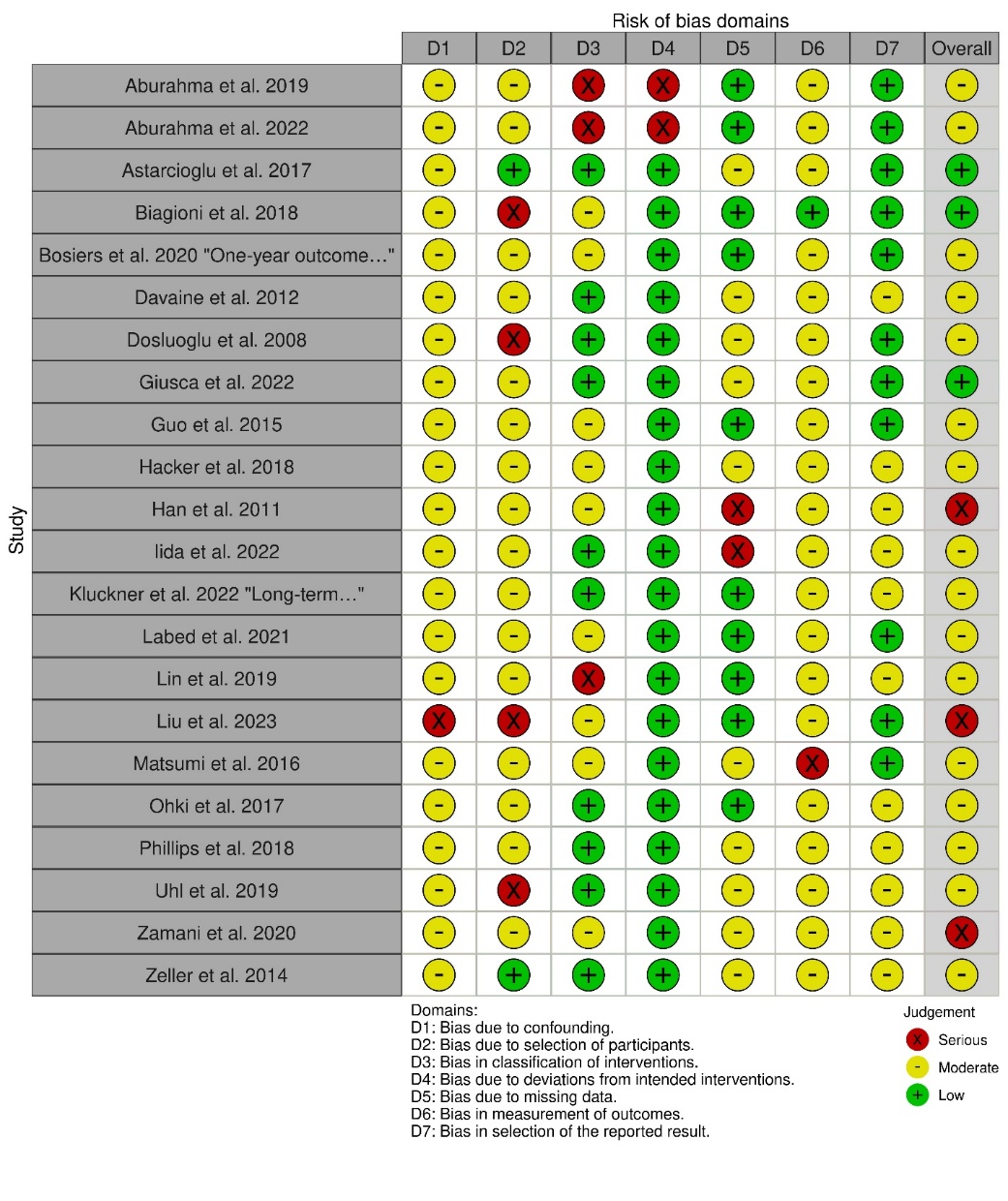


# **S6: Quality Appraisal: Publication Bias**

Visual inspection of funnel plots was conducted in order to evaluate sample size and publication bias. In all figures, the transformed event rate is plotted on the x-axis, with sample size on the y-axis. The overall pooled statistic is denoted with the solid vertical line, while the 95% confidence intervals are denoted with the dashed lines. In general, smaller sample sized studies were associated with greater variability in the observed event rate, with larger sample sizes demonstrating greater consistency or precision.

The Egger’s test results further suggest the presence of publication bias, with the resulting p-value<0.05 for all outcomes, except assisted primary patency.^33^

Figure S6.1: Funnel plot of 12-month primary patency


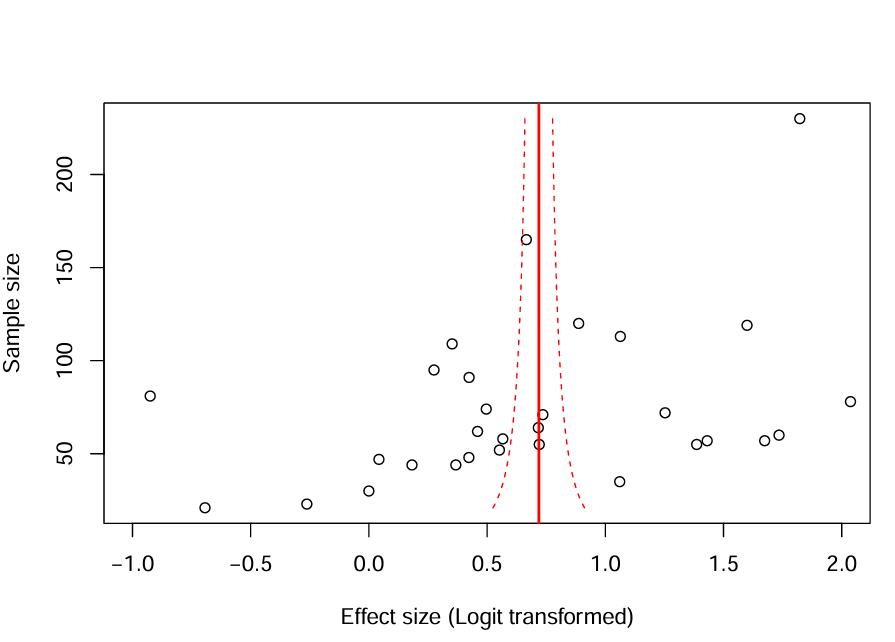


Figure S6.2: Funnel plot of 12-month secondary patency


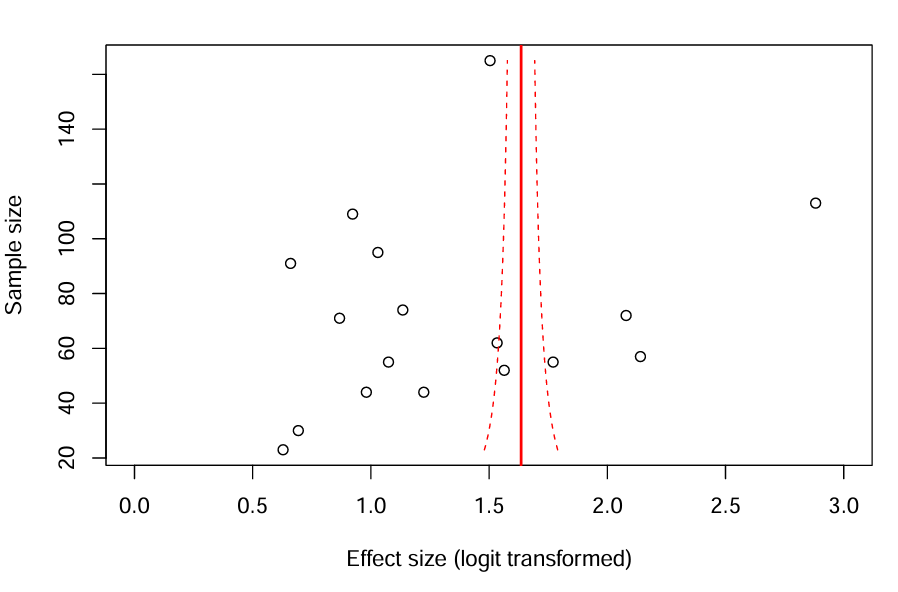


Figure S6.3: Funnel plot of 12-month ffTLR
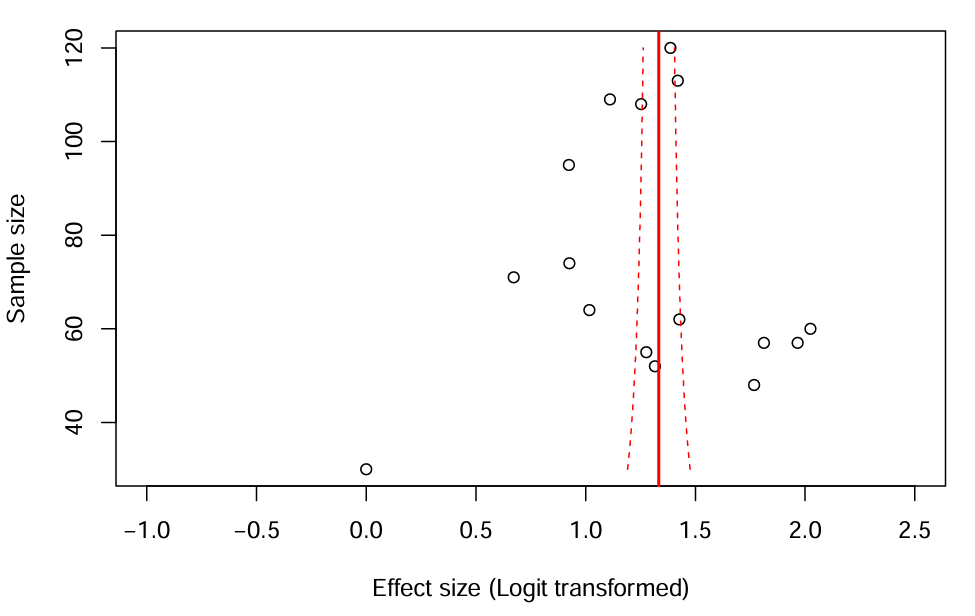


Figure S6.4: Funnel plot of 12-month assisted primary patency


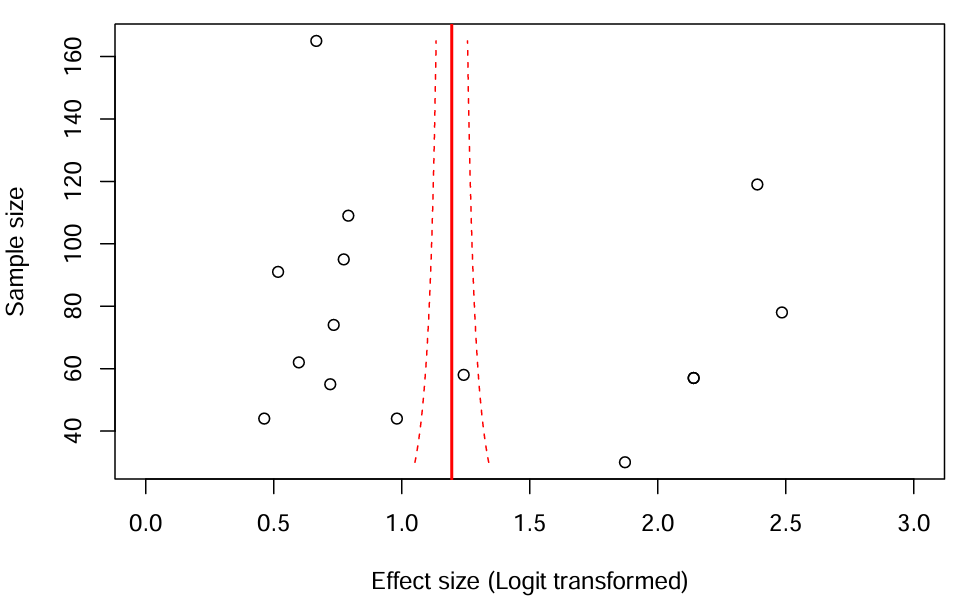


Figure S6.5: Funnel plot of 12-month mortality


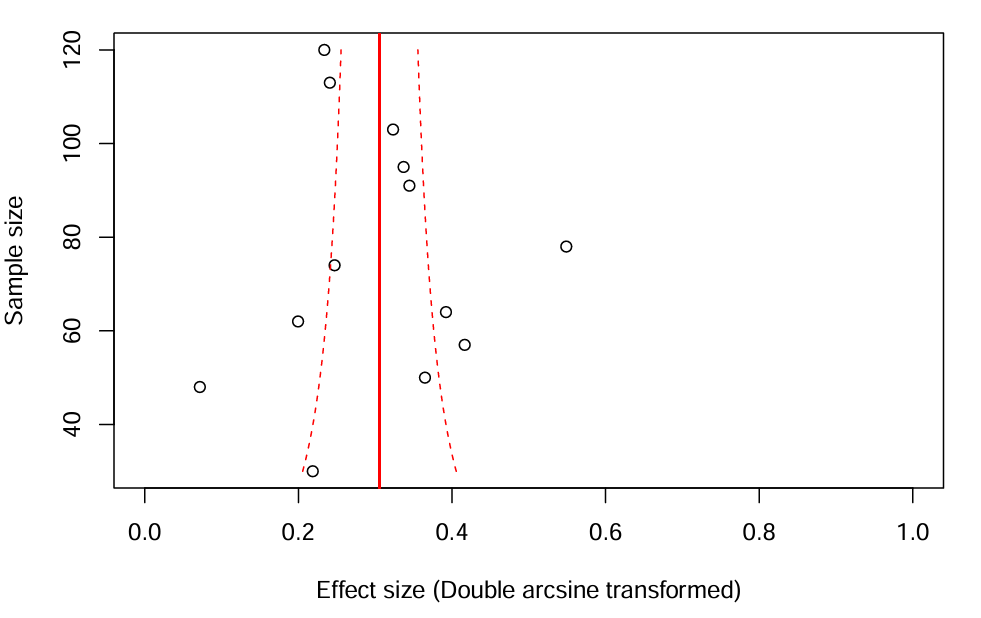


Figure S6.6: Funnel plot of 12-month limb salvage


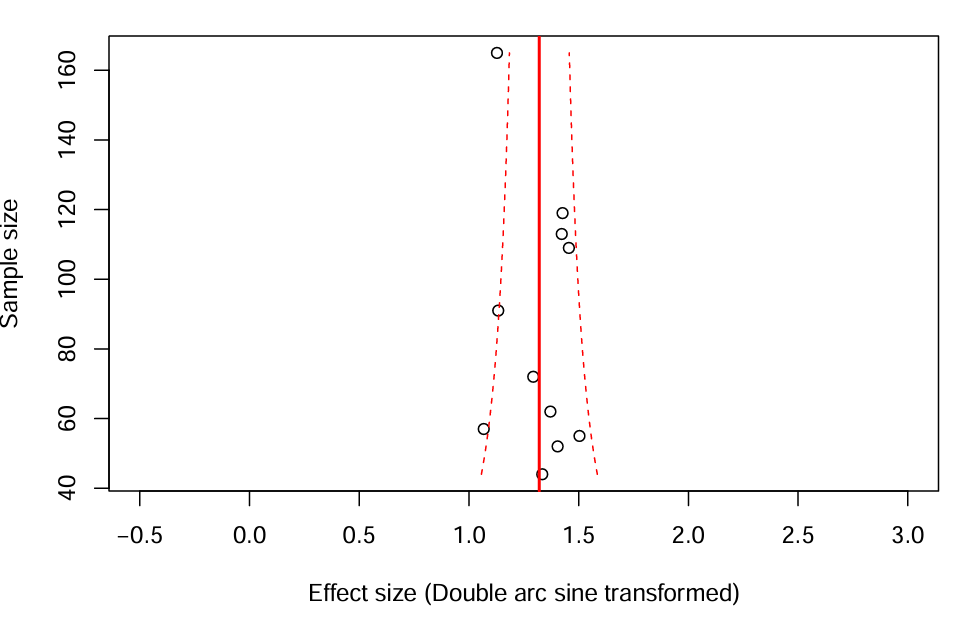


# **S7: Summary of Findings and Quality of Evidence**

The following tables provide a summary of outcomes for all scenarios explored, as well as the supporting quality of evidence and supporting judgement, based on GRADE criteria.^34^

S7.1: Summary of Primary Patency Outcomes and Quality of Evidence

|  | NUmber of observations | Total Sample | Pooled Estimate (95% CI) | Quality of evidence | Supporting Judgement |
| --- | --- | --- | --- | --- | --- |
| 6-Month | | | | | |
| Overall | 27 | 2,104 | 0.85 (0.81,0.89, I^2=77.8%, τ^2=0.47, **PI: 0.58, 0.96**) | ⊕⊕⊕⊝  Moderate | Downgraded due to considerable heterogeneity, moderate degree of bias; CIs are relatively tight – some precision concerns, high number of studies reporting. |
| Intervention |  |  |  |  |  |
| BMS | 12 | 790 | 0.84 (0.78,0.89; I^2=63.3%, τ^2=0.26) | ⊕⊕⊕⊝  Moderate | Downgraded due to moderate heterogeneity and bias; some precision concerns, fair number of studies reporting. |
| CS | 6 | 532 | 0.89 (0.79,0.95; I^2=77%; τ^2=0.39) | ⊕⊕⊕⊝  Moderate | Downgraded due to moderate heterogeneity and bias; some precision concerns, fair number of studies reporting. |
| DCB | 2 | 201 | 0.74 (0.01, 1.00; I^2=93.7%; τ^2=0.41) | ⊕⊕⊝⊝  Low | Downgraded due to considerable heterogeneity, CIs suggest some imprecision, few number of studies reporting. |
| DES | 4 | 247 | 0.87 (0.64, 0.96; I^2=80.8%; τ^2=0.52) | ⊕⊕⊝⊝  Low | Downgraded due to considerable heterogeneity, CIs suggest some imprecision, few number of studies reporting. |
| Other | 3 | 334 | 0.85 (0.37,0.98; I^2=83.3%, τ^2=0.71) | ⊕⊕⊝⊝  Low | Downgraded due to considerable heterogeneity, CIs suggest some imprecision, few number of studies reporting. |
| 12-MONTH | | | | | |
| Overall | 30 | 2,230 | 0.67 (0.61,0.73, I^2=83.7%; τ^2=0.39; **PI: 0.36,0.88**) | ⊕⊕⊕⊝  Moderate | Downgraded due to considerable heterogeneity, moderate degree of bias; CIs are relatively tight – some precision concerns, high number of studies reporting. |
|  |  |  |  |  |  |
| Lesion Length |  |  |  |  |  |
| NA | 8 | 714 | 0.69 (0.47,0.84, I^2=93.6%; τ^2=1.04) | ⊕⊕⊝⊝  Low | Downgraded due to moderate heterogeneity and bias; some precision concerns, fair number of studies reporting. |
| ≥20.0-24.9 | 5 | 417 | 0.76 (0.69,0.81, I^2=0.0%; τ^2=0) | ⊕⊕⊕⊕  High | No concerns related to heterogeneity or imprecision. Good number of studies reporting. |
| ≥25.0-29.9 | 13 | 918 | 0.64 (0.58,0.71, I^2=69.4%; τ^2=0.16) | ⊕⊕⊕⊝  Moderate | Downgraded due to considerable heterogeneity, moderate degree of bias; CIs are relatively tight – some precision concerns, high number of studies reporting. |
| ≥30.0+ | 4 | 181 | 0.60 (0.48,0.71, I^2=4.1%; τ^2=0) | ⊕⊕⊕⊝  Moderate | No concerns related to heterogeneity, however fewer studies reporting and some evidence of imprecision. |
| Definition |  |  |  |  |  |
| Group 1 | 20 | 1,185 | 0.65 (0.57,0.72, I^2=82.6%; τ^2=0.44) | ⊕⊕⊕⊝  Moderate | Downgraded due to considerable heterogeneity, moderate degree of bias; CIs are relatively tight – some precision concerns, high number of studies reporting. |
| Group 2 | 10 | 1,045 | 0.71 (0.62, 0.79, I^2=84.8%; τ^2=0.27) | ⊕⊕⊕⊝  Moderate | Downgraded due to considerable heterogeneity, moderate degree of bias; CIs are relatively tight – some precision concerns, high number of studies reporting. |
| Intervention |  |  |  |  |  |
| BMS | 13 | 813 | 0.65 (0.56, 0.72; I^2=75.8%; τ^2=0.27) | ⊕⊕⊕⊝  Moderate | Downgraded due to considerable heterogeneity, moderate degree of bias; CIs are relatively tight – some precision concerns, high number of studies reporting. |
| CS | 7 | 587 | 0.75 (0.64, 0.83; I^2=81.6%; τ^2=0.23) | ⊕⊕⊕⊝  Moderate | Downgraded due to considerable heterogeneity, moderate degree of bias; CIs are relatively tight – some precision concerns, fair number of studies reporting. |
| DCB | 2 | 201 | 0.50 (0.0, 1.0; I^2=96.9%; τ^2=0.79) | ⊕⊕⊝⊝  Low | Downgraded due to considerable heterogeneity, CIs suggest some imprecision, few number of studies reporting. |
| DES | 5 | 295 | 0.65 (0.48, 0.79; I^2=76.7%; τ^2=0.22) | ⊕⊕⊕⊝  Moderate | Downgraded due to considerable heterogeneity, moderate degree of bias; some precision concerns, fair number of studies reporting. |
| Other | 3 | 334 | 0.73 (0.33, 0.94; I^2=87.3%; τ^2=0.40) | ⊕⊕⊝⊝  Low | Downgraded due to considerable heterogeneity, CIs suggest some imprecision, few number of studies reporting. |
| Disease severity |  |  |  |  |  |
| mix | 27 | 2,052 | 0.67 (0.61, 0.73, I^2=84.2%; τ^2=0.39) | ⊕⊕⊕⊝  Moderate | Downgraded due to considerable heterogeneity, moderate degree of bias; CIs are relatively tight – some precision concerns, fair number of studies reporting. |
| CLTI | 3 | 178 | 0.67 (0.27, 0.92, I^2=83.5%; τ^2=0.37) | ⊕⊕⊝⊝  Low | Downgraded due to few studies reporting, imprecision, and considerable heterogeneity observed. |
| Study Design |  |  |  |  |  |
| RCT | 7 | 471 | 0.64 (0.49, 0.77, I^2=82.5%; τ^2=0.37) | ⊕⊕⊕⊝  Moderate | Downgraded due to considerable heterogeneity, moderate degree of bias; some precision concerns, fair number of studies reporting. |
| Non-RCT | 23 | 1759 | 0.68 (0.61, 0.74, I^2=84.7%; τ^2=0.40) | ⊕⊕⊕⊝  Moderate | Downgraded due to considerable heterogeneity, moderate degree of bias; some precision concerns, good number of studies reporting. |
| Publication year |  |  |  |  |  |
| 2018 or earlier | 13 | 771 | 0.67 (0.57, 0.75, I^2=74.4%; τ^2=0.34) | ⊕⊕⊕⊝  Moderate | Downgraded due to considerable heterogeneity, moderate degree of bias; some precision concerns, good number of studies reporting. |
| Post-2018 | 17 | 1,459 | 0.68 (0.59, 0.75, I^2=87.8%; τ^2=0.42) | ⊕⊕⊕⊝  Moderate | Downgraded due to considerable heterogeneity, moderate degree of bias; some precision concerns, good number of studies reporting. |
| 24-MONTH | | | | | |
| Overall | 20 | 1,475 | 0.48 (0.38,0.57, I^2=86.3%, τ^2=0.59, PI: 0.15, 0.83) | ⊕⊕⊕⊝  Moderate | Downgraded due to considerable heterogeneity and variation in effect between included studies. Some imprecision. |
| Intervention |  |  |  |  |  |
| BMS | 10 | 717 | 0.48 (0.34,0.61, I^2=85.2%, τ^2=0.54) | ⊕⊕⊕⊝  Moderate | Downgraded due to considerable heterogeneity and variation in effect between included studies. Some imprecision. |
| CS | 3 | 191 | 0.52 (0.34,0.70, I^2=54.6%, τ^2=0.03) | ⊕⊕⊕⊝  Moderate | Downgraded due to moderate heterogeneity and variation in effect between included studies. Some imprecision. |
| DCB | 1 | 81 | 0.14 (0.07,0.23) | NA | Data for descriptive purposes, only one study reporting. |
| DES | 3 | 152 | 0.42 (0.06,0.88, I^2=91.2%, τ^2=0.79) | ⊕⊕⊝⊝  Low | Downgraded due to few studies reporting, imprecision, and considerable heterogeneity observed. |
| Other | 3 | 334 | 0.61 (0.49,0.72, I^2=20.0%, τ^2=0.0) | ⊕⊕⊝⊝  Low | Downgraded due to few studies reporting, imprecision, and considerable heterogeneity observed. |
| 36-MONTH | | | | | |
| Overall | 14 | 1,024 | 0.42 (0.33,0.52, I^2=81.2%; τ^2=0.37, PI: 0.16, 0.74) | ⊕⊕⊕⊝  Moderate | Downgraded due to considerable heterogeneity and variation in effect between included studies. Some concerns of imprecision, good number of reporting studies. |
| Intervention |  |  |  |  |  |
| BMS | 9 | 662 | 0.38 (0.25,0.51, I^2=84.7%; τ^2=0.46) | ⊕⊕⊕⊝  Moderate | Downgraded due to considerable heterogeneity and variation in effect between included studies. Some concerns of imprecision, good number of reporting studies. |
| CS | 2 | 136 | 0.43 (0.08,0.87, I^2=0%; τ^2=0) | ⊕⊕⊝⊝  Low | Downgraded due imprecision and few number of studies reporting. |
| DES | 1 | 57 | 0.67 (0.53, 0.79) | NA | Data for descriptive purposes, only one study reporting. |
| Other | 2 | 169 | 0.51 (0.13, 0.88, I^2=0%; τ^2=0) | ⊕⊕⊝⊝  Low | Downgraded due imprecision and few number of studies reporting. |
| 48-MOnth | | | | | |
| Overall | 9 | 757 | 0.42 (0.35,0.49, I^2=68.4%, τ^2=0.09, PI: 0.25, 0.61) | ⊕⊕⊕⊝  Moderate | Downgraded due to considerable heterogeneity, low concern for imprecision. |
| 60-month | | | | | |
| Overall | 5 | 381 | 0.40 (0.31, 0.50, I^2=53.7%, τ^2=0.04, PI: 0.25, 0.57) | ⊕⊕⊕⊝  Moderate | Downgraded due to moderate heterogeneity, less robust reporting, some concerns of imprecision. |

S7.2: Summary of Secondary Patency Outcomes and Quality of Evidence

|  | NUmber of observations |  | Total Sample | Pooled Estimate (95% CI) | Quality of evidence | Supporting Judgement |
| --- | --- | --- | --- | --- | --- | --- |
| 6-Month | | | | | | |
| Overall | 15 |  | 1,106 | 0.90 (0.85,0.93, I^2=48.5%; τ^2=0.39, PI: 0.68, 0.97) | ⊕⊕⊕⊝  Moderate | Downgraded due to moderate heterogeneity, moderate degree of bias; CIs are relatively tight – high number of studies reporting. |
| Intervention |  |  |  |  |  |  |
| BMS | 9 |  | 586 | 0.92 (0.83,0.96, I^2=53.6%; τ^2=0.84) | ⊕⊕⊕⊝  Moderate | Downgraded due to moderate heterogeneity, moderate degree of bias; CIs are relatively tight – fair number of studies reporting. |
| CS | 3 |  | 133 | 0.90 (0.77,0.96, I^2=0%; τ^2=0) | ⊕⊕⊕⊝  Moderate | Downgraded due to few studies reporting, low concern for heterogeneity or imprecision. |
| DES | 1 |  | 57 | 0.93 (0.83,0.98) | NA | Data for descriptive purposes, only one study reporting. |
| Other | 2 |  | 256 | 0.83 (0.29,0.98, I^2=62.6%; τ^2=0.02) | ⊕⊕⊕⊝  Moderate | Downgraded due to moderate heterogeneity and evidence of imprecision. |
| 12-Month | | | | | | |
| Overall | 20 |  | 1,446 | 0.84 (0.77,0.89, I^2=75.8%, τ^2=0.69, PI: 0.46, 0.97) | ⊕⊕⊕⊝  Moderate | Downgraded due to considerable heterogeneity observed, some precision concerns, high number of studies reporting. |
|  |  |  |  |  |  |  |
| Lesion Length |  |  |  |  |  |  |
| NA | 2 |  | 209 | 0.81 (0.51, 0.99, I^2=0.0%; τ^2=0) | ⊕⊕⊝⊝  Low | Downgraded as CIs suggest imprecision, few studies reporting, however, no heterogeneity concerns. |
| ≥20.0-24.9 | 4 |  | 297 | 0.91 (0.83,0.96, I^2=34.3%; τ^2=0) | ⊕⊕⊕⊝  Moderate | Low to moderate heterogeneity, no concerns of imprecision, few studies reporting. |
| ≥25.0-29.9 | 11 |  | 807 | 0.79 (0.69,0.88, I^2=89.7%; τ^2=0.03) | ⊕⊕⊕⊝  Moderate | Downgraded due to considerable heterogeneity observed, some imprecision concerns, and fair number of studies reporting. |
| ≥30.0+ | 3 |  | 133 | 0.85 (0.34, 1.00, I^2=85.6%; τ^2=0.03) | ⊕⊕⊝⊝  Low | Downgraded due to considerable heterogeneity observed, CIs suggest imprecision, few studies reporting. |
| Intervention |  |  |  |  |  |  |
| BMS | 11 |  | 728 | 0.86 (0.74, 0.93, I^2=77%; τ^2=1.08) | ⊕⊕⊕⊝  Moderate | Downgraded due to considerable heterogeneity observed, some imprecision concerns, and fair number of studies reporting. |
| CS | 4 |  | 262 | 0.78 (0.67, 0.86, I^2=38.8%; τ^2=0.02) | ⊕⊕⊕⊝  Moderate | Low to moderate heterogeneity, no concerns of imprecision, few studies reporting. |
| DES | 3 |  | 200 | 0.88 (0.41, 0.99, I^2=87%; τ^2=0.70) | ⊕⊕⊝⊝  Low | Downgraded due to considerable heterogeneity observed, CIs suggest imprecision, few studies reporting. |
| Other | 2 |  | 256 | 0.75 (0.06, 0.99, I^2=87.4%; τ^2=0.13) | ⊕⊕⊝⊝  Low | Downgraded due to considerable heterogeneity observed, CIs suggest imprecision, few studies reporting. |
| Definition |  |  |  |  |  |  |
| Group 1 | 12 |  | 882 | 0.83 (0.73,0.90, I^2=76.9%; τ^2=0.73) | ⊕⊕⊕⊝  Moderate | Downgraded due to considerable heterogeneity observed, low concern for imprecision, fair number of studies reporting. |
| Group 2 | 4 |  | 219 | 0.87 (0.62,0.97, I^2=65.4%; τ^2=0.60) | ⊕⊕⊕⊝  Moderate | Downgraded due to moderate heterogeneity, some imprecision and few studies reporting. |
| Group 3 | 4 |  | 345 | 0.82 (0.56, 0.94, I^2=82.5%; τ^2=0.55) | ⊕⊕⊕⊝  Moderate | Downgraded due to considerable heterogeneity, imprecision, and few studies reporting. |
| Disease severity |  |  |  |  |  |  |
| mix | 17 |  | 1,268 | 0.84 (0.77, 0.89, I^2=73.3%; τ^2=0.54) | ⊕⊕⊕⊝  Moderate | Downgraded due to moderate heterogeneity observed, low concern for imprecision, and good number of studies reporting. |
| CLTI | 3 |  | 178 | 0.84 (0.11, 1.00, I^2=81.3%; τ^2=1.96) | ⊕⊕⊕⊝  Moderate | Downgraded due to considerable heterogeneity, imprecision, and few studies reporting. |
| Study design |  |  |  |  |  |  |
| RCT | 5 |  | 415 | 0.87 (0.59, 0.97, I^2=88.5%; τ^2=1.45) | ⊕⊕⊕⊝  Moderate | Downgraded due to considerable heterogeneity, imprecision, and few studies reporting. |
| Non-RCT | 15 |  | 1,031 | 0.82 (0.75, 0.87, I^2=67.7%; τ^2=0.40) | ⊕⊕⊕⊝  Moderate | Downgraded due to moderate heterogeneity observed, low concern for imprecision, and good number of studies reporting. |
| Publication year |  |  |  |  |  |  |
| 2018 or earlier | 8 |  | 529 | 0.81 (0.72, 0.88, I^2=64.6%; τ^2=0.29) | ⊕⊕⊕⊝  Moderate | Downgraded due to moderate heterogeneity observed, low concern for imprecision, and fair number of studies reporting. |
| Post-2018 | 12 |  | 917 | 0.85 (0.75, 0.92, I^2=81.1%; τ^2=0.95) | ⊕⊕⊕⊝  Moderate | Downgraded due to considerable heterogeneity observed, low concern for imprecision, good number of studies reporting. |
|  |  |  |  |  |  |  |
| 24-Month | | | | | | |
| Overall | 15 |  | 1,157 | 0.72 (0.64,0.78, I^2=77.7%; τ^2=0.32; PI: 0.42, 0.90) | ⊕⊕⊕⊝  Moderate | Downgraded due to considerable heterogeneity observed, low concern for imprecision, good number of studies reporting. |
| Intervention |  |  |  |  |  |  |
| BMS | 9 |  | 653 | 0.71 (0.59,0.81, I^2=80.4%; τ^2=0.42) | ⊕⊕⊕⊝  Moderate | Downgraded due to considerable heterogeneity observed, some concern for imprecision, fair number of studies reporting. |
| CS | 3 |  | 191 | 0.72 (0.38,0.91, I^2=79%; τ^2=0.23) | ⊕⊕⊝⊝  Low | Downgraded due to considerable heterogeneity observed, CIs suggest imprecision, few studies reporting. |
| DES | 1 |  | 57 | 0.81 (0.68,0.90) | NA | Data for descriptive purposes, only one study reporting. |
| Other | 2 |  | 256 | 0.71 (0.06,0.99, I^2=87.4%; τ^2=0.12) | ⊕⊕⊝⊝  Low | Downgraded due to considerable heterogeneity observed, CIs suggest imprecision, few studies reporting. |
|  |  |  |  |  |  |  |
| 36-Month | | | | | | |
| Overall | 12 |  | 882 | 0.63 (0.52,0.73, I^2=85.3%; τ^2=0.45; PI: 0.27, 0.89) | ⊕⊕⊕⊝  Moderate | Downgraded due to considerable and significant heterogeneity and variation in effect between included studies. Some imprecision. |
| Intervention |  |  |  |  |  |  |
| BMS | 8 |  | 598 | 0.63 (0.46,0.77, I^2=88.6%; τ^2=0.56 | ⊕⊕⊕⊝  Moderate | Downgraded due to considerable heterogeneity observed, some concern for imprecision, fair number of studies reporting. |
| CS | 2 |  | 136 | 0.57 (0.13,0.92, I^2=0%; τ^2=0) | ⊕⊕⊝⊝  Low | Downgraded for few studies reporting and imprecision, low concern for heterogeneity |
| DES | 1 |  | 57 | 0.82 (0.70,0.91) | NA | Data for descriptive purposes, only one study reporting |
| Other | 1 |  | 91 | 0.54 (0.43, 0.64) | NA | Data for descriptive purposes, only one study reporting |
| 48-month | | | | | | |
| Overall | 8 |  | 679 | 0.62 (0.49, 0.74, I^2=86.5%; τ^2=0.36; PI: 0.27, 0.88) | ⊕⊕⊕⊝  Moderate | Downgraded due to considerable heterogeneity and variation in effect between included studies. Concerns of imprecision and fewer studies reporting. |
| 60-Month | | | | | | |
| Overall | 5 |  | 381 | 0.59 (0.45,0.72, I^2=78.4% τ^2=0.17; PI: 0.29,0.84) | ⊕⊕⊕⊝  Moderate | Downgraded due to considerable heterogeneity, less robust sample and some concerns of imprecision. |

S7.3: Summary of Assisted Primary Patency Outcomes and Quality of Evidence

|  | NUmber of observations |  | Total Sample | Pooled Estimate (95% CI) | Quality of evidence | Supporting Judgement |
| --- | --- | --- | --- | --- | --- | --- |
| 6-Month | | | | | | |
| Overall | 12 |  | 880 | 0.85 (0.80,0.90, I^2=62%; τ^2=0.24; PI: 0.65,0.95) | ⊕⊕⊕⊝  Moderate | Downgraded due to moderate heterogeneity, no precision concerns, good number of studies reporting. |
|  |  |  |  |  |  |  |
| 12-Month | | | | | | |
| Overall | 15 |  | 1,138 | 0.77 (0.69, 0.83, I^2=78.4%; τ^2=0.40; PI: 0.45, 0.93) | ⊕⊕⊕⊝  Moderate | Downgraded due to considerable heterogeneity, low precision concerns, good number of studies reporting. |
|  |  |  |  |  |  |  |
| Lesion Length |  |  |  |  |  |  |
| NA | 3 |  | 287 | 0.79 (0.35,0.96, I^2=87.5%; τ^2=0.51) | ⊕⊕⊝⊝  Low | Downgraded due to considerable heterogeneity, CIs suggest imprecision, few studies reporting. |
| ≥20.0-24.9 | 1 |  | 57 | 0.89 (0.78,0.96) | NA | Only one study reporting. |
| ≥25.0-29.9 | 10 |  | 736 | 0.74 (0.64, 0.82, I^2=77.3%; τ^2=0.35) | ⊕⊕⊕⊝  Moderate | Downgraded due to moderate heterogeneity observed, some imprecision concerns, fair number of studies reporting. |
| ≥30.0+ | 1 |  | 58 | 0.78 (0.65,0.87) | NA | Only one study reporting. |
| Definition |  |  |  |  |  |  |
| group 1 | 9 |  | 630 | 0.77 (0.67, 0.85, I^2=78.6%; τ^2=0.34) | ⊕⊕⊕⊝  Moderate | Downgraded due to moderate heterogeneity, slight concern of imprecision, but fair number of studies reporting. |
| group 2 | 2 |  | 135 | 0.91 (0.18, 1.00, I^2=0%; τ^2=0) | ⊕⊕⊝⊝  Low | Few studies reporting, no concerns of heterogeneity, but evidence of imprecision. |
| group 3 | 4 |  | 373 | 0.66 (0.58, 0.74, I^2=0%; τ^2=0) | ⊕⊕⊕⊝  Moderate | Fair number of studies reporting, no concerns of heterogeneity, and little concern of imprecision. |
| Disease severity |  |  |  |  |  |  |
| mix | 12 |  | 960 | 0.76 (0.67, 0.83, I^2=78.1%; τ^2=0.37) | ⊕⊕⊕⊝  Moderate | Downgraded due to moderate heterogeneity observed, some imprecision, and fair number of studies reporting. |
| CLTI | 3 |  | 178 | 0.81 (0.37, 0.97, I^2=86.2%; τ^2=0.48) | ⊕⊕⊕⊝  Moderate | Downgraded due to few studies reporting, imprecision, and considerable heterogeneity. |
| Intervention |  |  |  |  |  |  |
| BMS | 8 |  | 581 | 0.76 (0.65, 0.85, I^2=77.6%; τ^2=0.32) | ⊕⊕⊕⊝  Moderate | Downgraded due to moderate heterogeneity, slight concern of imprecision, but fair number of studies reporting. |
| CS | 2 |  | 136 | 0.66 (0.16, 0.95, I^2=0%; τ^2=0) | ⊕⊕⊝⊝  Low | Few studies reporting, imprecision, no concerns of heterogeneity. |
| des | 2 |  | 87 | 0.89 (0.10, 1.00, I^2=0%; τ^2=0) | ⊕⊕⊝⊝  Low | Few studies reporting, imprecision, no concerns of heterogeneity. |
| other | 3 |  | 334 | 0.76 (0.27, 0.97, I^2=88.9%; τ^2=0.66) | ⊕⊕⊝⊝  Low | Few studies reporting, imprecision, considerable heterogeneity. |
| study design |  |  |  |  |  |  |
| RCT | 4 |  | 302 | 0.82 (0.59, 0.93, I^2=84%; τ^2=0.37) | ⊕⊕⊝⊝  Low | Few studies reporting, imprecision, considerable heterogeneity. |
| non-RCT | 11 |  | 836 | 0.75 (0.65, 0.82, I^2=75.5%; τ^2=0.36) | ⊕⊕⊕⊝  Moderate | Downgraded due to moderate heterogeneity, slight concern of imprecision, but fair number of studies reporting. |
| publication year |  |  |  |  |  |  |
| 2018 or earlier | 5 |  | 389 | 0.76 (0.57, 0.88, I^2=79.1%; τ^2=0.36) | ⊕⊕⊕⊝  Moderate | Downgraded due to moderate heterogeneity, slight concern of imprecision, few studies reporting. |
| post-2018 | 10 |  | 749 | 0.77 (0.67, 0.85, I^2=80.2%; τ^2=0.41) | ⊕⊕⊕⊝  Moderate | Downgraded due to moderate heterogeneity, slight concern of imprecision, but fair number of studies reporting. |
| 24-Month | | | | | | |
| Overall | 14 |  | 1,108 | 0.61 (0.51, 0.71, I^2=84.9%; τ^2=0.43; PI: 0.27,0.88) | ⊕⊕⊕⊝  Moderate | Downgraded due to considerable heterogeneity and variation in effect between included studies. Some imprecision. |
|  |  |  |  |  |  |  |
| 36-Month | | | | | | |
| Overall | 11 |  | 779 | 0.57 (0.42, 0.71, I^2=90.5%; τ^2=0.75; PI: 0.15, 0.91) | ⊕⊕⊕⊝  Moderate | Downgraded due to considerable heterogeneity and variation in effect between included studies. Some imprecision. |
| 48-month | | | | | | |
| Overall | 8 |  | 685 | 0.59 (0.44,0.72, I^2=89.0%; τ^2=0.44; PI: 0.21, 0.88) | ⊕⊕⊝⊝  Low | Downgraded due to considerable heterogeneity and variation in effect between included studies. Some imprecision. Fair number of studies reporting. |
| 60-Month | | | | | | |
| Overall | 4 |  | 309 | 0.53 (0.34, 0.71, I^2=81.5%; τ^2=0.18; PI: 0.19, 0.84) | ⊕⊕⊝⊝  Low | Downgraded due to considerable heterogeneity and variation in effect between included studies. Some imprecision. Fewer studies reporting. |

S7.4: Summary of ffTLR Outcomes and Quality of Evidence

|  | NUmber of observations |  | Total Sample | Pooled Estimate (95% CI) | Quality of evidence | Supporting Judgement |
| --- | --- | --- | --- | --- | --- | --- |
| 6-Month | | | | | | |
| Overall | 14 |  | 1,030 | 0.93 (0.89,0.96, I^2=27%; τ^2=0.43; PI: 0.76, 0.98) | ⊕⊕⊕⊝  Moderate | Downgraded due some evidence of heterogeneity, low precision concerns, good number of studies reporting. |
| Intervention |  |  |  |  |  |  |
| bms | 6 |  | 437 | 0.91 (0.85,0.95, I^2=44.1%; τ^2=0.14) | ⊕⊕⊕⊝  Moderate | Downgraded due to moderate heterogeneity, slight concern of imprecision, but few studies reporting. |
| cs | 4 |  | 260 | 0.93 (0.78,0.98, I^2=56.7%; τ^2=0.36) | ⊕⊕⊕⊝  Moderate | Downgraded due to moderate heterogeneity, slight concern of imprecision, but few studies reporting. |
| DCB | 1 |  | 120 | 0.90 (0.83,0.95) | NA | Data provided for descriptive purposes, only one study reporting. |
| des | 2 |  | 105 | 0.93 (0.09,1.00, I^2=0%; τ^2=0) | ⊕⊕⊝⊝  Low | Few studies reporting, imprecision, no concerns of heterogeneity. |
| other | 1 |  | 108 | 1.0 (0.97,1.00) | NA | Data provided for descriptive purposes, only one study reporting. |
| 12-Month | | | | | | |
| Overall | 17 |  | 1,230 | 0.79 (0.74,0.83, I^2=61.2%; τ^2=0.19; PI: 0.59, 0.91) | ⊕⊕⊕⊝  Moderate | Downgraded due to moderate heterogeneity, no precision concerns, good number of reporting studies. |
| Intervention |  |  |  |  |  |  |
| bms | 7 |  | 494 | 0.77 (0.72,0.81, I^2=0%; τ^2=0) | ⊕⊕⊕⊕  High | No concerns related to heterogeneity or imprecision. Good number of studies reporting. |
| cs | 4 |  | 260 | 0.84 (0.52,0.96, I^2=83.1%; τ^2=0.79) | ⊕⊕⊕⊝  Moderate | Downgraded due to considerable heterogeneity and variation in effect between included studies. Some imprecision. |
| DCB | 1 |  | 120 | 0.80 (0.72,0.87) | NA | Only one study reporting |
| des | 4 |  | 248 | 0.79 (0.53,0.93, I^2=82.3%; τ^2=0.45) | ⊕⊕⊕⊝  Moderate | Downgraded due to considerable heterogeneity and variation in effect between included studies. Some imprecision. |
| other | 1 |  | 108 | 0.78 (0.69,0.85) |  | Only one study reporting |
| Lesion Length |  |  |  |  |  |  |
| NA | 1 |  | 60 | 0.88 (0.77,0.95) | NA | Only one study reporting. |
| ≥20.0-24.9 | 6 |  | 515 | 0.83 (0.75,0.89, I^2=43.7%; τ^2=0.09) | ⊕⊕⊕⊝  Moderate | Downgraded due to moderate heterogeneity observed, slight imprecision, fair number of studies reporting. |
| ≥25.0-29.9 | 8 |  | 555 | 0.73 (0.66,0.78, I^2=52.4%; τ^2=0.06) | ⊕⊕⊕⊝  Moderate | Downgraded due to moderate heterogeneity, low imprecision concerns, fair number of studies reporting. |
| ≥30.0+ | 2 |  | 100 | 0.82 (0.14,0.99, I^2=0.0%; τ^2=0) | ⊕⊕⊝⊝  Low | Only two studies reporting, no concerns of heterogeneity, but high degree of imprecision. |
| Definition |  |  |  |  |  |  |
| ffTLR | 8 |  | 528 | 0.81 (0.71,0.87, I^2=61.5%; τ^2=0.24) | ⊕⊕⊕⊝  Moderate | Downgraded due to moderate heterogeneity, minimal concern of imprecision, fair number of studies reporting. |
| CD-FfTLR | 9 |  | 702 | 0.78 (0.71,0.84, I^2=65.3%; τ^2=0.15) | ⊕⊕⊕⊝  Moderate | Downgraded due to moderate heterogeneity, no concern of imprecision, fair number of studies reporting. |
| Disease severity |  |  |  |  |  |  |
| mix | 15 |  | 1,143 | 0.80 (0.75, 0.83, I^2=49.3%; τ^2=0.11) | ⊕⊕⊕⊝  Moderate | Downgraded due to moderate heterogeneity, no concern of imprecision, fair number of studies reporting. |
| CLTI | 2 |  | 87 | 0.72 (0.0, 1.00, I^2=91.5%; τ^2=0.72) | ⊕⊕⊝⊝  Low | Only two studies reporting, substantial heterogeneity and high degree of imprecision. |
| Study design |  |  |  |  |  |  |
| rct | 3 |  | 252 | 0.72 (0.38, 0.91, I^2=81.4%; τ^2=0.24) | ⊕⊕⊝⊝  Low | Few studies reporting, considerable heterogeneity and imprecision. |
| non-rct | 14 |  | 978 | 0.80 (0.75, 0.85, I^2=54.9%; τ^2=0.15) | ⊕⊕⊕⊝  Moderate | Downgraded due to moderate heterogeneity, no concern of imprecision, good number of studies reporting. |
| publication year |  |  |  |  |  |  |
| 2018 and earlier | 5 |  | 293 | 0.80 (0.69, 0.88, I^2=63.4%; τ^2=0.13) | ⊕⊕⊕⊝  Moderate | Downgraded due to moderate heterogeneity, slight concern of imprecision, few studies reporting. |
| post-2018 | 12 |  | 937 | 0.79 (0.72, 0.84, I^2=63.5%; τ^2=0.22) | ⊕⊕⊕⊝  Moderate | Downgraded due to moderate heterogeneity, slight concern of imprecision, good number of studies reporting. |
| 24-Month | | | | | | |
| Overall | 10 |  | 722 | 0.68 (0.62,0.73, I^2=52.2%; τ^2=0.06; PI: 0.53, 0.79) | ⊕⊕⊕⊝  Moderate | Downgraded due to moderate heterogeneity, slight variation in effect between included studies. |
| intervention |  |  |  |  |  |  |
| bms | 5 |  | 380 | 0.70 (0.63,0.76, I^2=0%; τ^2=0) | ⊕⊕⊕⊕  High | No concerns related to heterogeneity or imprecision. Fair number of studies reporting. |
| cs | 2 |  | 129 | 0.66 (0.08,0.98, I^2=68.2%; τ^2=0.04) | ⊕⊕⊕⊝  Moderate | Downgraded due to some heterogeneity and variation in effect between included studies. Some imprecision. |
| des | 2 |  | 105 | 0.71 (0.14, 0.98; I^2=50.1%; τ^2=0.0) | ⊕⊕⊕⊝  Moderate | Downgraded due to some heterogeneity and variation in effect between included studies. Some imprecision. |
| other | 1 |  | 108 | 0.54 (0.44, 0.63) | NA | Only one study reporting. |
| 36-Month | | | | | | |
| Overall | 6 |  | 456 | 0.63 (0.57,0.69, I^2=24.3%; τ^2=0; PI: 0.57, 0.69) | ⊕⊕⊕⊝  Moderate | Low/moderate heterogeneity suggests studies are consistent, rather precise CI, fair sample reporting. |
| intervention |  |  |  |  |  |  |
| bms | 4 |  | 325 | 0.62 (0.54,0.71, I^2=0%; τ^2=0; | ⊕⊕⊕⊕  High | No concerns related to heterogeneity or imprecision. Fair number of studies reporting. |
| cs | 1 |  | 74 | 0.55 (0.43,0.67) | NA | Only one study reporting. |
| des | 1 |  | 57 | 0.77 (0.64, 0.87) | NA | Only one study reporting. |
| 48-month | | | | | | |
| Overall | 4 |  | 335 | 0.59 (0.50, 0.67, I^2=0.0%; τ^2=0; PI: 0.50, 0.67) | ⊕⊕⊕⊕  High | Fewer studies reporting, but low heterogeneity suggests studies are consistent and precise sample. |
| 60-Month | | | | | | |
| Overall | 2 |  | 169 | 0.53 (0.05, 0.96, I^2=77.7%; τ^2=0.06; PI: 0.01, 0.99) | ⊕⊕⊝⊝  Low | Downgraded due to considerable heterogeneity, high imprecision. |

S7.5: Summary of Mortality Outcomes and Quality of Evidence

|  | NUmber of observations |  | Total Sample | Pooled Estimate (95% CI) | Quality of evidence | Supporting Judgement |
| --- | --- | --- | --- | --- | --- | --- |
| 6-Month | | | | | | |
| Overall | 8 |  | 528 | 0.04 (0.0,0.10, I^2=80.0%; τ^2=0.01; PI: 0.0, 0.25) | ⊕⊕⊕⊝  Moderate | Downgraded due to moderate heterogeneity, low precision concerns, fair number of studies reporting. |
|  |  |  |  |  |  |  |
| 12-Month | | | | | | |
| Overall | 13 |  | 985 | 0.08 (0.05, 0.13, I^2=73.2%; τ^2=0.01; PI: 0.0, 0.25) | ⊕⊕⊕⊝  Moderate | Downgraded due to moderate heterogeneity, low precision concerns, fair number of studies reporting. |
|  |  |  |  |  |  |  |
| Lesion Length |  |  |  |  |  |  |
| NA | 1 |  | 78 | 0.27 (0.17,0.38) | NA | Only one study reporting. |
| ≥20.0-24.9 | 3 |  | 290 | 0.08 (0.00,0.26, I^2=66.4%; τ^2=0.01) | ⊕⊕⊕⊝  Moderate | Moderate heterogeneity, some imprecision and few studies reporting. |
| ≥25.0-29.9 | 8 |  | 569 | 0.09 (0.06,0.12, I^2=16.4%; τ^2=0.0) | ⊕⊕⊕⊕  High | Low heterogeneity, fair number of reporting studies and no imprecision concerns. |
| ≥30.0+ | 1 |  | 48 | 0.0 (0.0, 0.07) | NA | Only one study reporting. |
| Disease severity |  |  |  |  |  |  |
| mix | 11 |  | 864 | 0.09 (0.04, 0.14, I^2=76.9%; τ^2=0.01) | ⊕⊕⊕⊝  Moderate | Downgraded due to moderate heterogeneity observed, some imprecision, and fair number of studies reporting. |
| CLTI | 2 |  | 121 | 0.08 (0.00, 0.76, I^2=31.0%; τ^2=0.0) | ⊕⊕⊕⊝  Moderate | Downgraded due to few studies reporting and imprecision, low concern for heterogeneity. |
| intervention |  |  |  |  |  |  |
| bms | 5 |  | 360 | 0.08 (0.01, 0.19, I^2=70.8%; τ^2=0.01) | ⊕⊕⊕⊝  Moderate | Downgraded due to moderate heterogeneity observed, some imprecision, and fair number of studies reporting. |
| cs | 2 |  | 136 | 0.04 (0.00, 0.25, I^2=0%; τ^2=0) | ⊕⊕⊕⊝  Moderate | Downgraded due to few studies reporting and imprecision, low concern for heterogeneity. |
| dcb | 1 |  | 120 | 0.05 (0.02, 0.10) | NA | Only one study reporting. |
| des | 3 |  | 200 | 0.08 (0.00, 0.28, I^2=62.9%; τ^2=0.0) | ⊕⊕⊕⊝  Moderate | Downgraded due to few studies reporting and imprecision, some concern for heterogeneity. |
| other | 2 |  | 169 | 0.18 (0.00, 1.0, I^2=85.8%; τ^2=0.02) | ⊕⊕⊕⊝  Moderate | Downgraded due to few studies reporting and imprecision, moderate concern for heterogeneity. |
| study design |  |  |  |  |  |  |
| rct | 3 |  | 246 | 0.07 (0.01, 0.15, I^2=0%; τ^2=0.0) | ⊕⊕⊕⊝  Moderate | Downgraded due to few studies reporting and imprecision, low concern for heterogeneity. |
| non-rct | 10 |  | 739 | 0.09 (0.04, 0.15, I^2=78.3%; τ^2=0.01) | ⊕⊕⊕⊝  Moderate | Downgraded imprecision, moderate concern for heterogeneity |
| publication year |  |  |  |  |  |  |
| 2018 or earlier | 2 |  | 126 | 0.09 (0.0, 1.00, I^2=96.3%; τ^2=0.11) | ⊕⊕⊕⊝  Moderate | Downgraded due to few studies reporting and imprecision, substantial concern for heterogeneity. |
| post-2018 | 11 |  | 859 | 0.08 (0.06, 0.11, I^2=35.4%; τ^2=0.0) | ⊕⊕⊕⊕  High | Low heterogeneity, fair number of reporting studies and no imprecision concerns. |
| 24-Month | | | | | | |
| Overall | 12 |  | 821 | 0.18 (0.13,0.23, I^2=68.9%; τ^2=0.0; PI: 0.05,0.36) | ⊕⊕⊕⊝  Moderate | Downgraded due to moderate heterogeneity, slight variation in effect between included studies, no concerns of imprecision. |
|  |  |  |  |  |  |  |
| 36-Month | | | | | | |
| Overall | 7 |  | 521 | 0.23 (0.12,0.36, I^2=86.2%; τ^2=0.02; PI: 0.01,0.60) | ⊕⊕⊕⊝  Moderate | Downgraded due to considerable heterogeneity, some imprecision and less robust sample. |
| 48-month | | | | | | |
| Overall | 8 |  | 679 | 0.26 (0.16,0.39, I^2=87.7%; τ^2=0.02; PI: 0.03,0.62) | ⊕⊕⊕⊝  Moderate | Downgraded due to considerable heterogeneity, some concern for imprecision and fair sample reporting. |
| 60-Month | | | | | | |
| Overall | 4 |  | 309 | 0.39 (0.20, 0.61, I^2=86.7%; τ^2=0.27; PI: 0.09,0.81) | ⊕⊕⊕⊝  Moderate | Downgraded due to considerable heterogeneity, imprecision and less robust sample. |

S7.6: Summary of Limb Salvage Outcomes and Quality of Evidence

|  | NUmber of observations |  | Total Sample | Pooled Estimate (95% CI) | Quality of evidence | Supporting Judgement |
| --- | --- | --- | --- | --- | --- | --- |
| 6-Month | | | | | | |
| Overall | 7 |  | 578 | 0.96 (0.90,1.00, I^2=84.4%; τ^2=0.01; PI: 0.76,1.00) | ⊕⊕⊕⊝  Moderate | Downgraded due to considerable heterogeneity observed, little concern of imprecision, fair number of reporting studies. |
|  |  |  |  |  |  |  |
| 12-Month | | | | | | |
| Overall | 11 |  | 939 | 0.94 (0.89,0.98, I^2=87.5%; τ^2=0.01; PI: 0.71,1.00) | ⊕⊕⊕⊝  Moderate | Downgraded due to considerable heterogeneity observed, little concern of imprecision, fair number of reporting studies. |
|  |  |  |  |  |  |  |
| Lesion Length |  |  |  |  |  |  |
| NA | 2 |  | 209 | 0.89 (0.0, 1.00, I^2=83.2% τ^2=0.01) | ⊕⊕⊝⊝  Low | Downgraded due to considerable heterogeneity, concern of imprecision, few reporting studies. |
| ≥20.0-24.9 | 2 |  | 185 | 0.96 (0.26,1.00, I^2=66.5%; τ^2=0.0) | ⊕⊕⊝⊝  Low | Downgraded due to moderate heterogeneity, concern of imprecision, few reporting studies. |
| ≥25.0-29.9 | 6 |  | 493 | 0.95 (0.83, 1.00, I^2=89.7%; τ^2=0.03) | ⊕⊕⊕⊝  Moderate | Downgraded due to considerable heterogeneity observed, some concern of imprecision, fair number of reporting studies. |
| ≥30.0+ | 1 |  | 52 | 0.98 (0.90, 1.00) | NA | Only one study reporting. |
| Disease severity |  |  |  |  |  |  |
| mix | 9 |  | 791 | 0.97 (0.92, 0.99, I^2=83.9%; τ^2=0.01) | ⊕⊕⊕⊝  Moderate | Downgraded due to considerable heterogeneity observed, little concern of imprecision, fair number of reporting studies. |
| CLTI | 2 |  | 148 | 0.80 (0.41, 1.00, I^2=0.0%; τ^2=0.0) | ⊕⊕⊕⊝  Moderate | Downgraded due to few studies reporting and imprecision. No concerns of heterogeneity. |
| intervention |  |  |  |  |  |  |
| bms | 7 |  | 508 | 0.96 (0.89, 1.00, I^2=81.2%; τ^2=0.02) | ⊕⊕⊕⊝  Moderate | Downgraded due to considerable heterogeneity observed, little concern of imprecision, fair number of reporting studies. |
| cs | 1 |  | 62 | 0.97 (0.89,1.00) | NA | Only one study reporting |
| des | 1 |  | 113 | 0.98 (0.94, 1.00) | NA | Only one study reporting |
| other | 2 |  | 256 | 0.82 (0.79, 0.85, I^2=0.0%; τ^2=0.0) | ⊕⊕⊕⊝  Moderate | Downgraded due to few studies reporting. No concerns of heterogeneity. |
| study design |  |  |  |  |  |  |
| rct | 4 |  | 385 | 0.98 (0.96, 1.00, I^2=0.0%; τ^2=0.0) | ⊕⊕⊕⊕  High | Low heterogeneity, few reporting studies and no imprecision concerns. |
| non-rct | 7 |  | 554 | 0.92 (0.81, 0.98, I^2=86.8%; τ^2=0.02) | ⊕⊕⊕⊝  Moderate | Downgraded due to considerable heterogeneity observed, little concern of imprecision, fair number of reporting studies. |
| publication year |  |  |  |  |  |  |
| 2018 or earlier | 4 |  | 333 | 0.92 (0.79, 1.00, I^2=81.6%; τ^2=0.01) | ⊕⊕⊕⊝  Moderate | Downgraded due to considerable heterogeneity observed, little concern of imprecision, few reporting studies. |
| post-2018 | 7 |  | 606 | 0.95 (0.86, 1.00, I^2=88.2%; τ^2=0.02) | ⊕⊕⊕⊝  Moderate | Downgraded due to considerable heterogeneity observed, little concern of imprecision, fair number of reporting studies. |
| 24-Month | | | | | | |
| Overall | 12 |  | 941 | 0.90 (0.82,0.96, I^2=89.7%; τ^2=0.03; PI: 0.58,1.00) | ⊕⊕⊕⊝  Moderate | Downgraded due to considerable heterogeneity observed, little concern of imprecision, fair number of reporting studies. |
| Disease severity |  |  |  |  |  |  |
| mix | 10 |  | 793 | 0.92 (0.84, 0.97, I^2=89.9%; τ^2=0.02) | ⊕⊕⊕⊝  Moderate | Downgraded due to considerable heterogeneity observed, low imprecision, and fair number of studies reporting. |
| CLTI | 2 |  | 148 | 0.76 (0.53, 0.93, I^2=0.0%; τ^2=0.0) | ⊕⊕⊕⊝  Moderate | Downgraded due to few studies reporting and imprecision. No concerns of heterogeneity. |
|  |  |  |  |  |  |  |
| 36-Month | | | | | | |
| Overall | 8 |  | 618 | 0.84 (0.72,0.93, I^2=88.2%; τ^2=0.03; PI: 0.47,1.00) | ⊕⊕⊕⊝  Moderate | Downgraded due to considerable heterogeneity observed, little concern of imprecision, and fair number of studies reporting. |
| Disease severity |  |  |  |  |  |  |
| mix | 6 |  | 470 | 0.88 (0.74, 0.97, I^2=87.1%; τ^2=0.02) | ⊕⊕⊕⊝  Moderate | Downgraded due to considerable heterogeneity, slight imprecision, and fair number of studies reporting. |
| CLTI | 2 |  | 148 | 0.71 (0.62, 0.79, I^2=0.0%; τ^2=0.0) | ⊕⊕⊕⊝  Moderate | Downgraded due to few studies reporting and slight imprecision. No concerns of heterogeneity. |
|  |  |  |  |  |  |  |
| 48-month | | | | | | |
| Overall | 4 |  | 381 | 0.86 (0.62, 0.96, I^2=90.2%; τ^2=0.55) | ⊕⊕⊕⊝  Moderate | Downgraded due to substantial heterogeneity observed, imprecision, and fair number of studies reporting. |
| 60-Month | | | | | | |
| Overall | 2 |  | 134 | 0.76 (0.0, 1.0, I^2=96.4%; τ^2=2.26; PI: 0.00,1.00) | ⊕⊕⊝⊝  Low | Downgraded due to substantial heterogeneity observed, high degree of imprecision and only two studies reporting. |

# **S8: Additional Results**

Additional results, including overall forest plots for the remaining outcomes evaluated (assisted primary patency, mortality, and limb salvage) and for definition subgroup analyses are provided below. In all forest plots, the study-observed cumulative event rates are denoted by the point estimates (grey), with corresponding confidence intervals represented with horizontal blue lines. The overall pooled estimate is denoted at the bottom (diamond blue point), with dashed vertical reference line. In addition, analyses exploring the relationship between 12-month outcomes and MLL and stratified by intervention are also detailed and include studies reporting a MLL specific to the cohort analyzed. The figures depict the study-observed 12-month cumulative event rates, plotted by MLL on the x-axis, and stratified by intervention, including DCB (green), DES (blue), Other (magenta), BMS (red), and CS (gold).

Figure S8.1: Relationship between 12-month Primary Patency and lesion length, by intervention


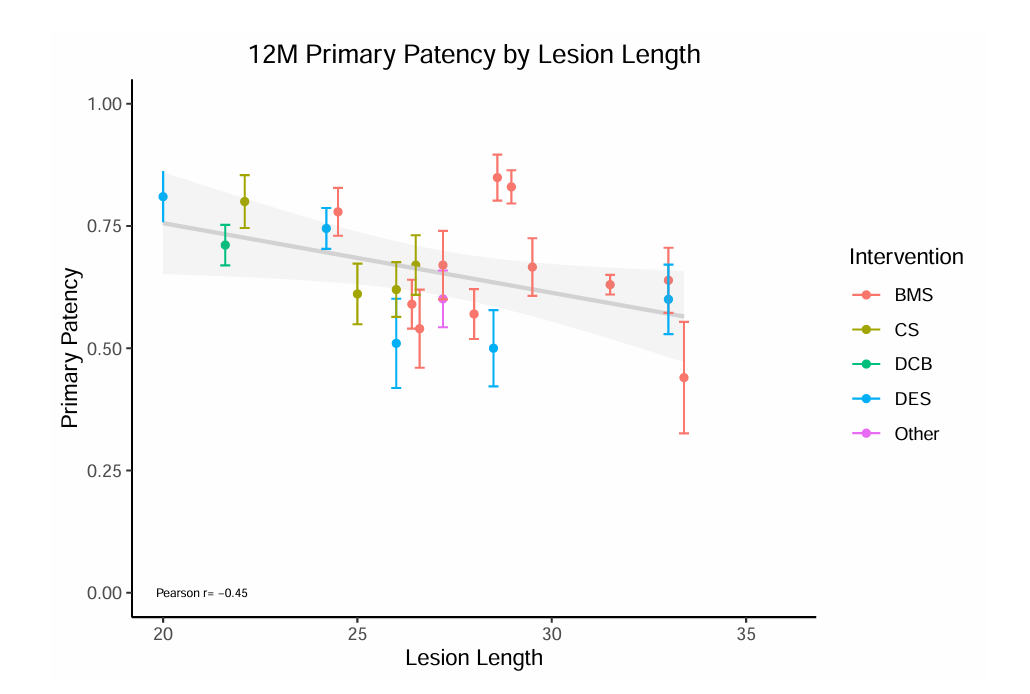


BMS: Bare metal stents; CS: Covered stents; DCB: drug coated balloon; DES: drug eluting stent.

Figure S8.2: Relationship between 12-month ffTLR and lesion length, by intervention


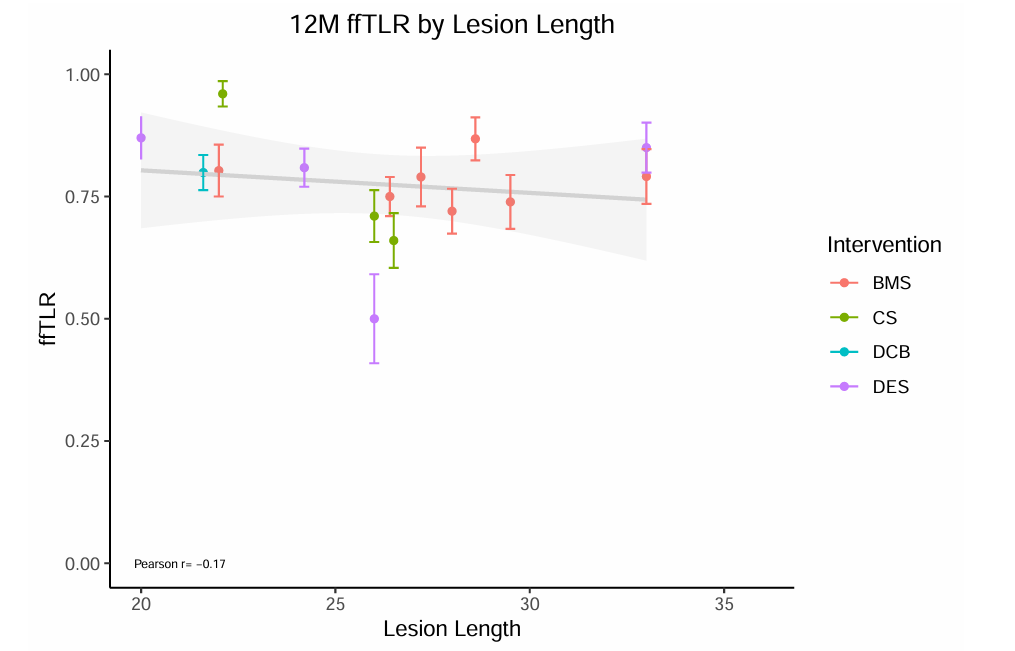


BMS: Bare metal stents; CS: Covered stents; DCB: drug coated balloon; DES: drug eluting stent; ffTLR: Freedom from target lesion revascularization.

Figure S8.3: Relationship between 12-month secondary patency and lesion length, by intervention


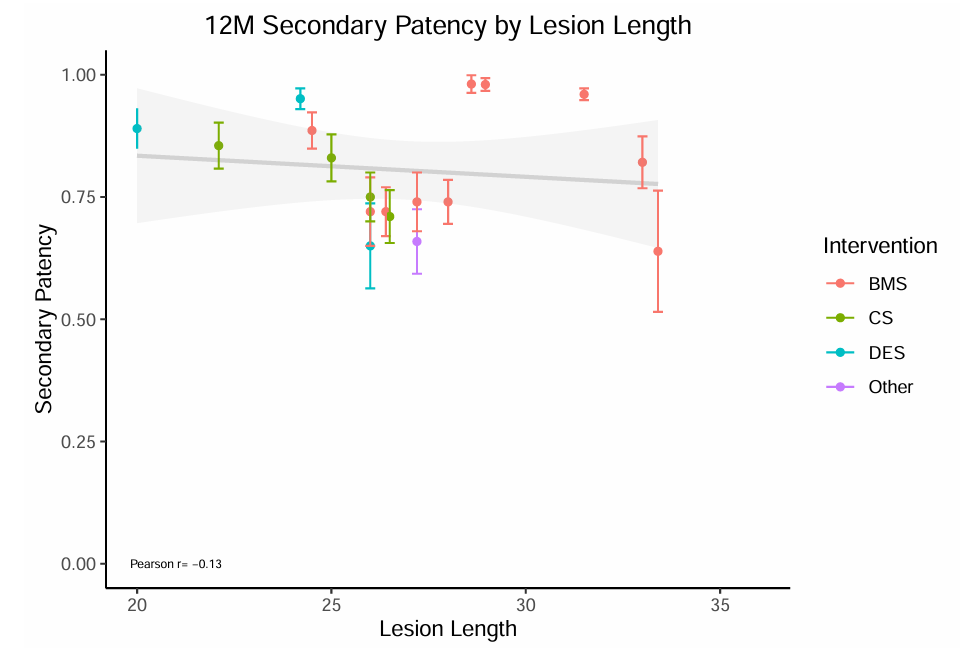


BMS: Bare metal stents; CS: Covered stents; DCB: drug coated balloon; DES: drug eluting stent.

Figure S8.4: Relationship between 12-month limb salvage and lesion length, by intervention


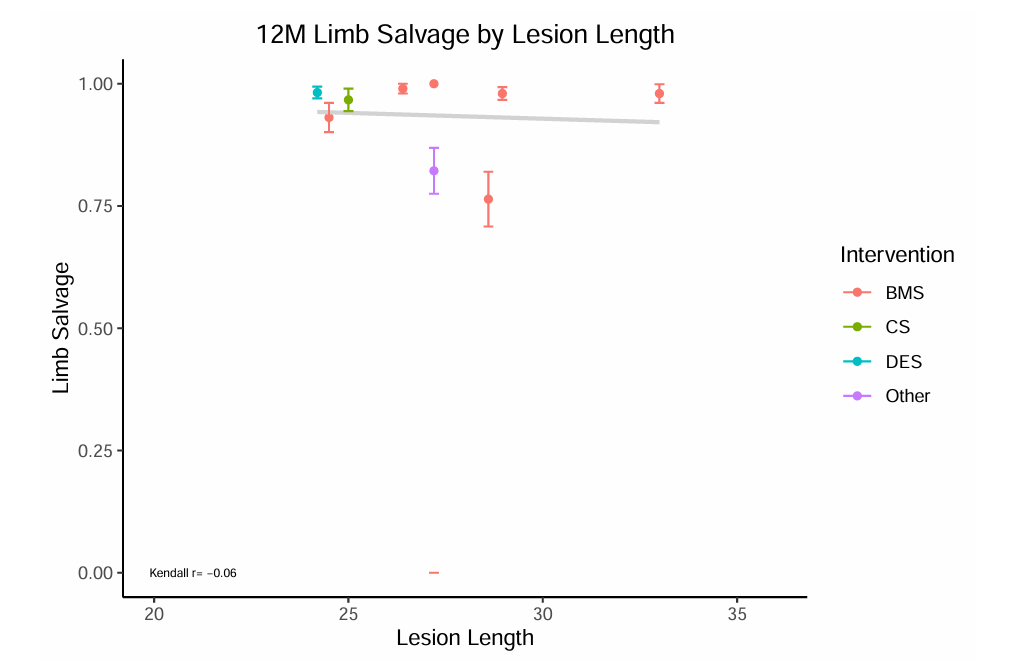


BMS: Bare metal stents; CS: Covered stents; DCB: drug coated balloon; DES: drug eluting stent.

Figure S8.5: Relationship between 12-month assisted primary patency and lesion length, by intervention


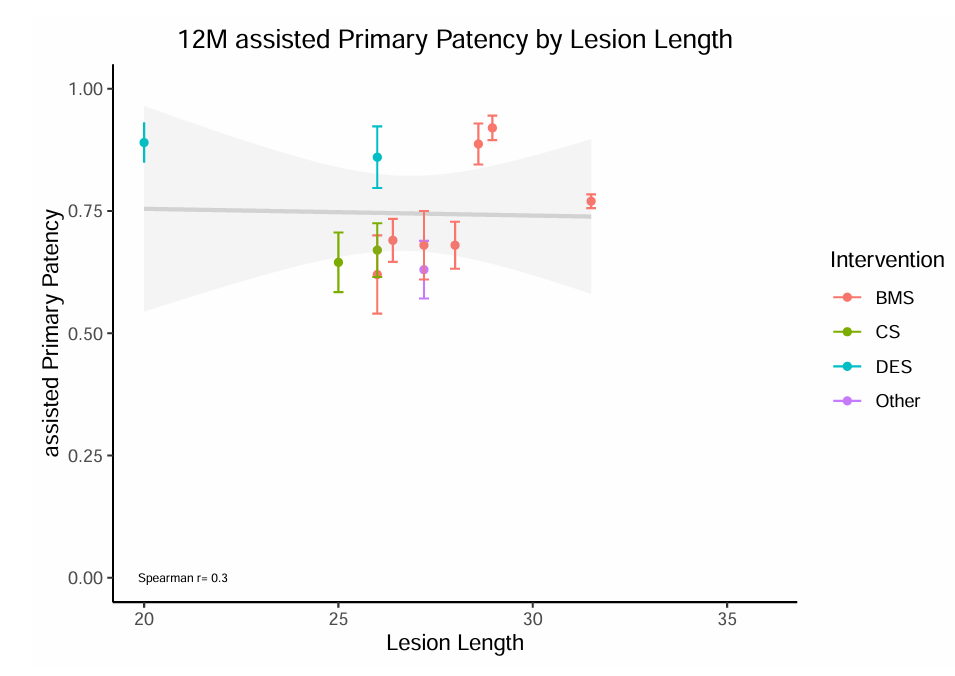


BMS: Bare metal stents; CS: Covered stents; DCB: drug coated balloon; DES: drug eluting stent; VIA: Viabahn endoprosthesis

Figure S8.6: Relationship between 12-month mortality and lesion length, by intervention


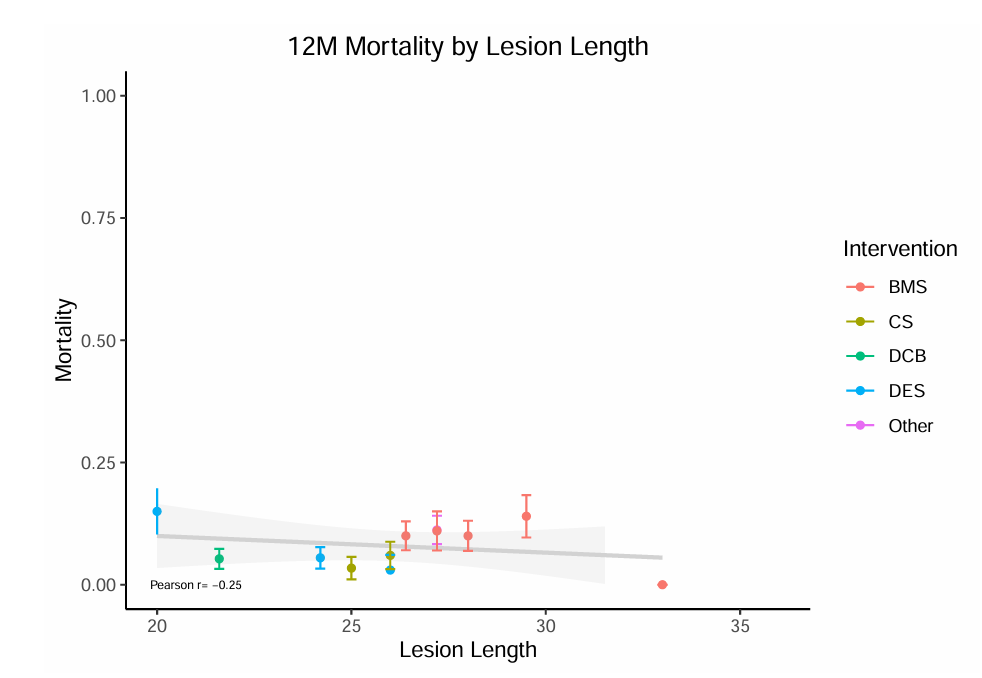


BMS: Bare metal stents; CS: Covered stents; DCB: drug coated balloon; DES: drug eluting stent.

*Figure* S8*.7: Six-Month Primary Patency Overall*


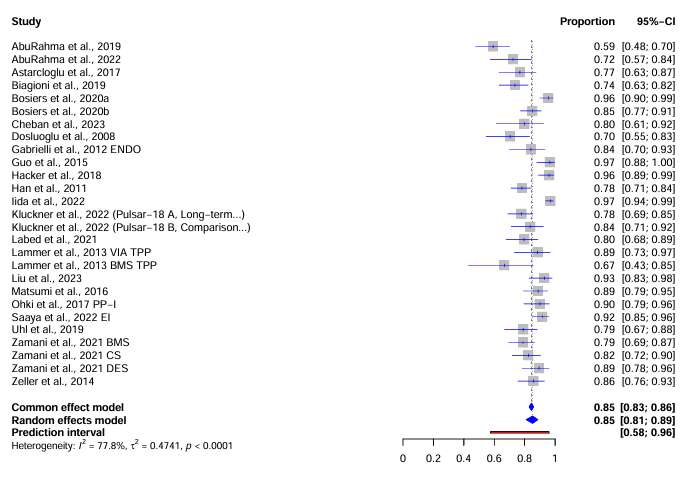


CI: Confidence Interval.

*Figure* S8*.8: Six-Month Primary Patency, by Intervention*


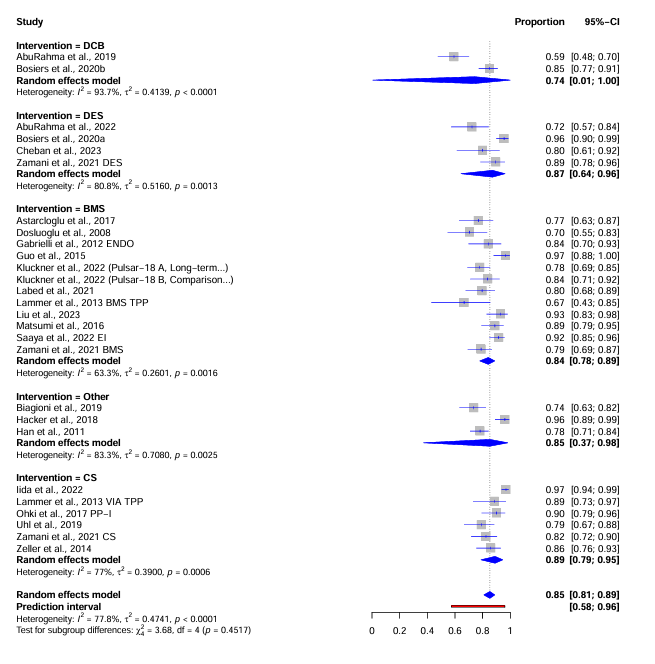


CI: Confidence Interval.

*Figure* S8*.9: 12-Month Primary Patency Overall*


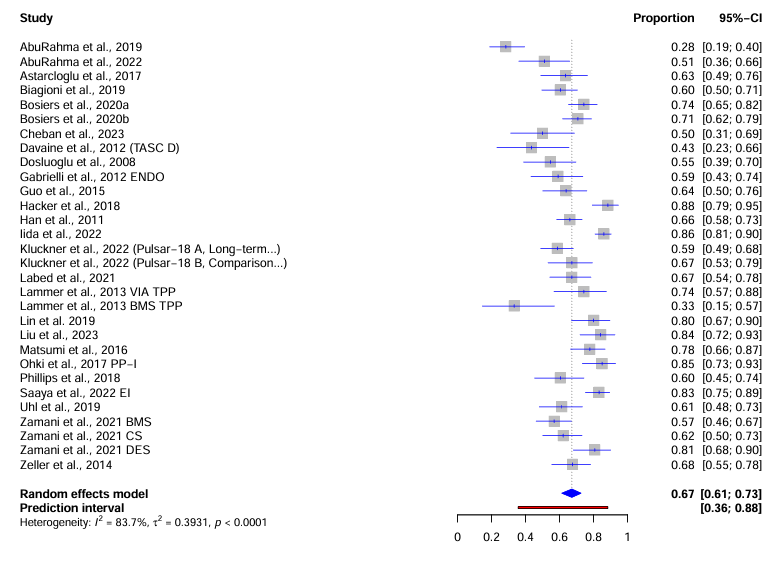


CI: Confidence Interval.

*Figure* S8*.10: 12-Month Primary Patency, by Lesion Length*


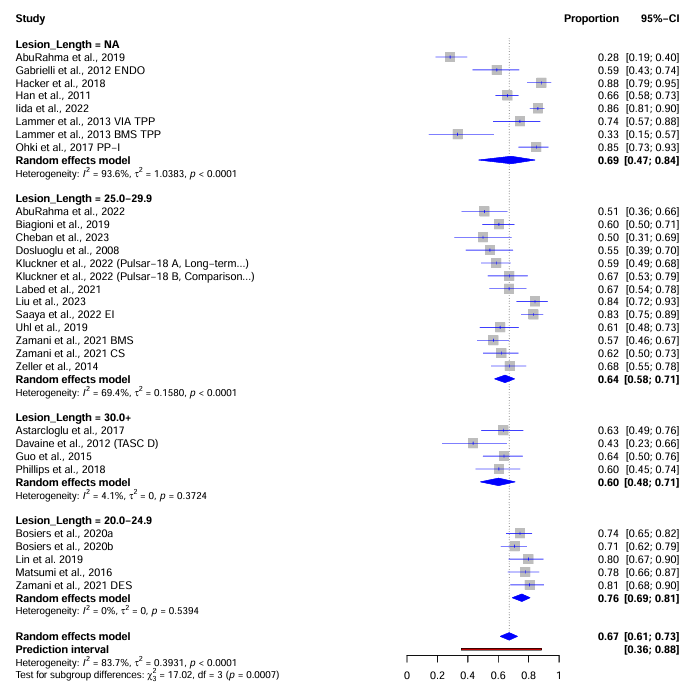


CI: Confidence Interval.

*Figure* S8*.11: 12-Month Primary Patency, by Disease Severity*


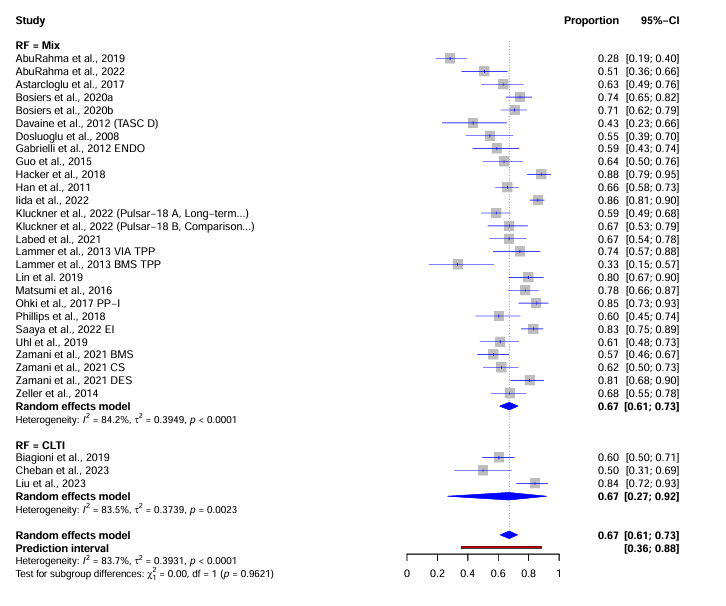


CI: Confidence Interval.

*Figure* S8*.12: 12-Month Primary Patency, by Outcome Definition*


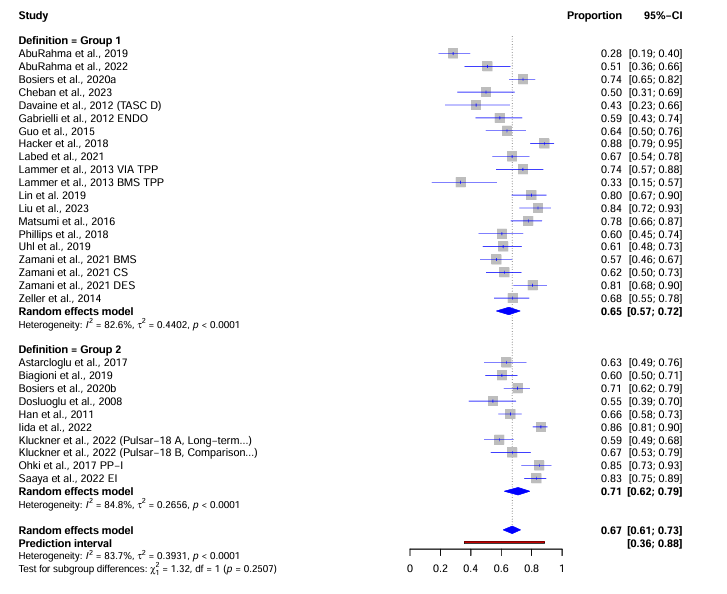


CI: Confidence Interval.

*Figure* S8*.13: 12-Month Primary Patency, by Intervention*


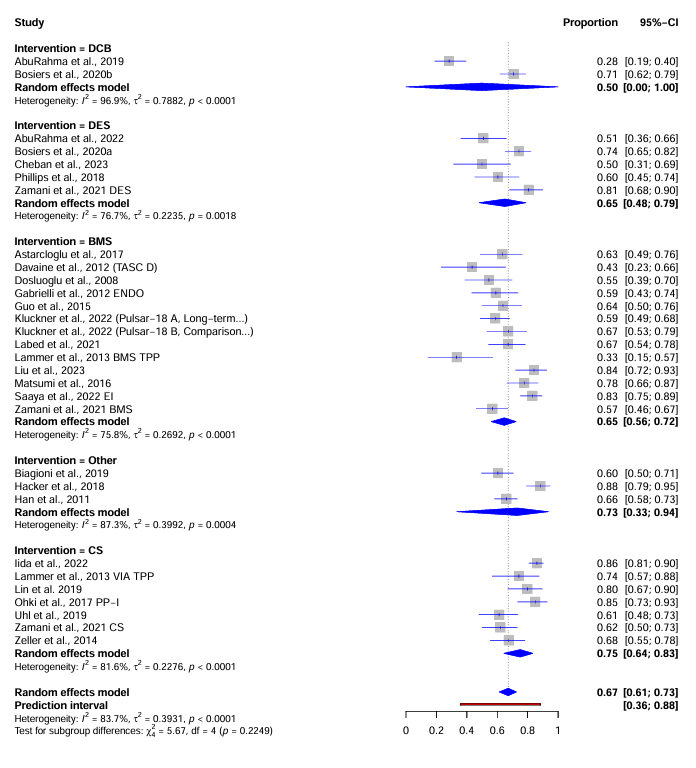


CI: Confidence Interval.

*Figure* S8*.14: 12-Month Primary Patency, by study design*


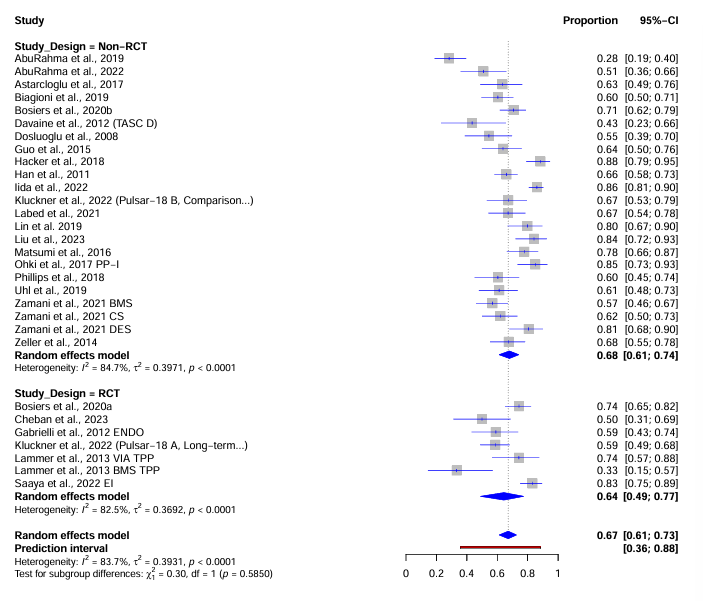


CI: Confidence Interval.

*Figure* S8*.15: 12-Month Primary Patency, by publication year*


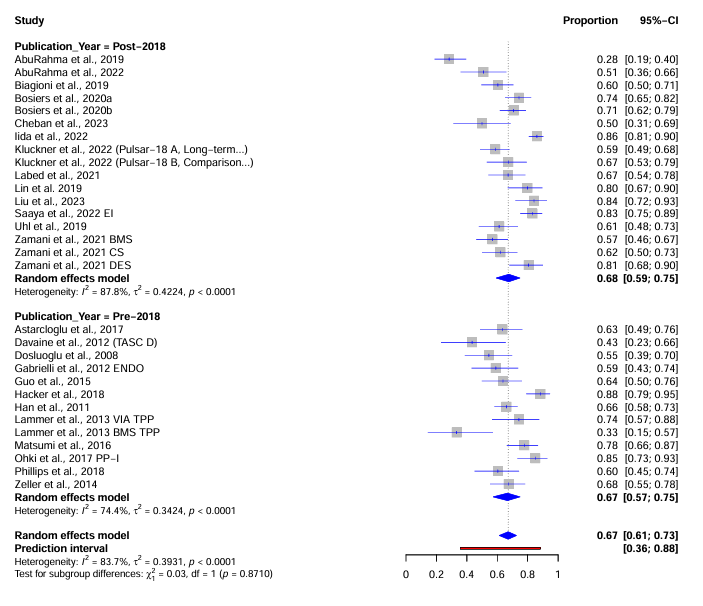


CI: Confidence Interval.

*Figure* S8*.16: 24-Month overall Primary Patency*


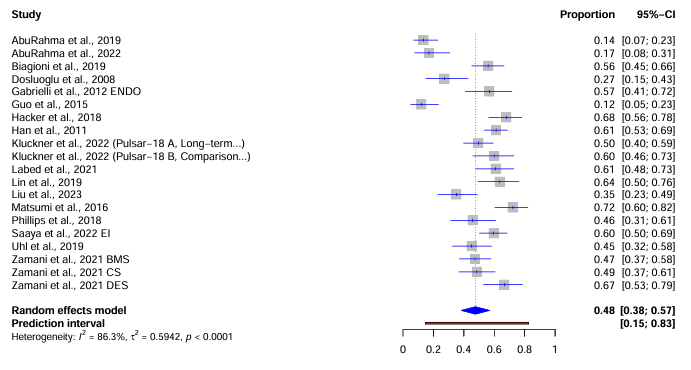


CI: Confidence Interval.

*Figure* S8*.17: 24-Month Primary Patency, by intervention*


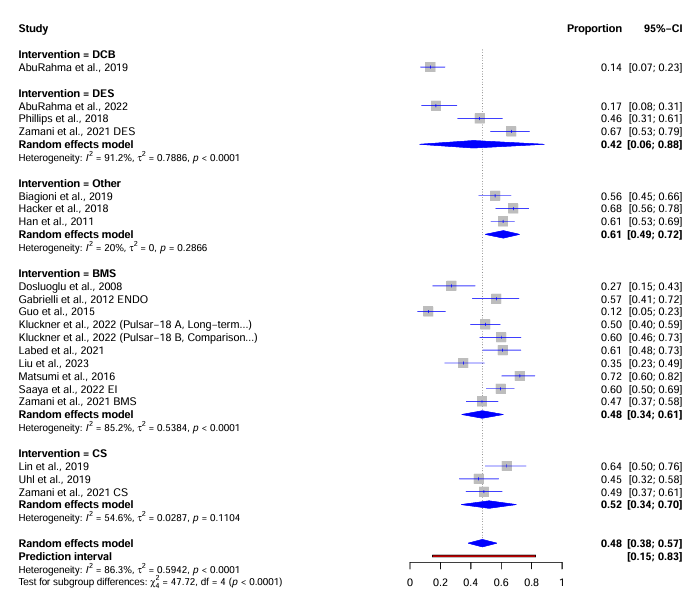


CI: Confidence Interval.

*Figure* S8*.18: 36-Month Overall primary Patency*


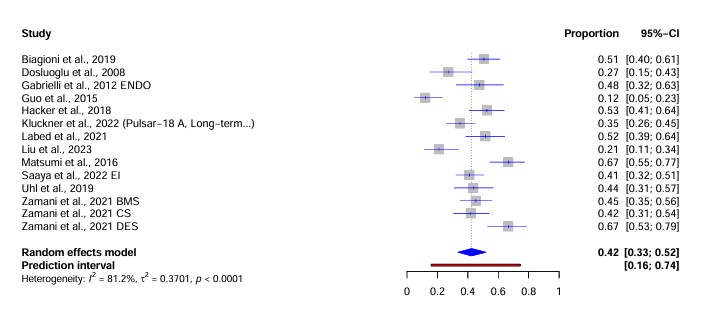


CI: Confidence Interval.

Figure S8.19: 36-Month Primary Patency, by Intervention


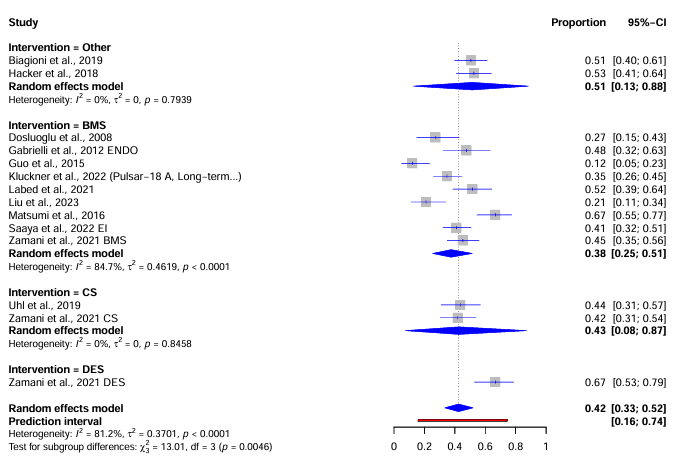


CI: Confidence Interval.

*Figure* S8*.20: 48-Month overall Primary Patency*


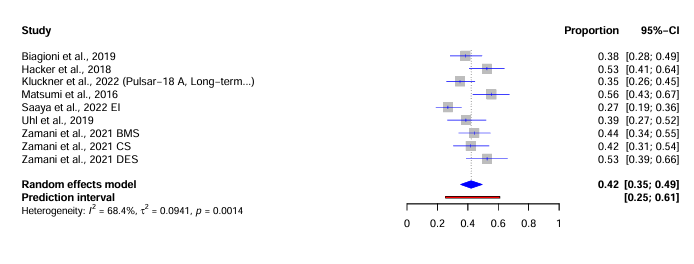


CI: Confidence Interval.

*Figure* S8*.21: 60-Month Overall Primary Patency*


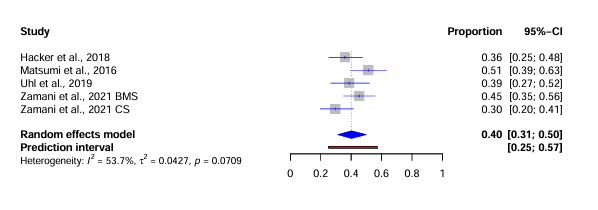


CI: Confidence Interval.

*Figure* S8*.22: Six-Month overall secondary patency*


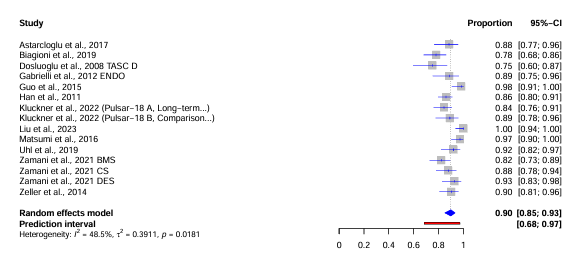


CI: Confidence Interval.

*Figure* S8*.23: Six-Month Secondary patency, by intervention*


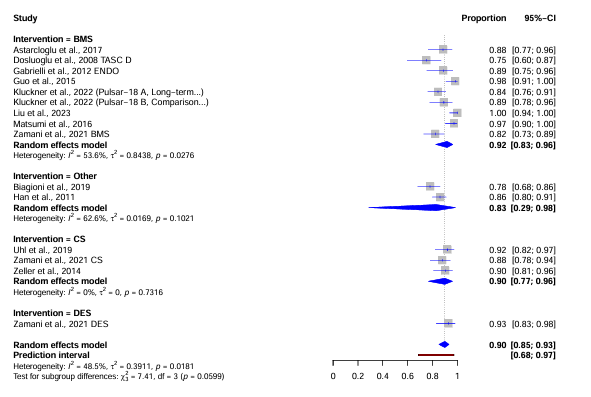


CI: Confidence Interval.

*Figure* S8*.24: 12-Month secondary patency overall*


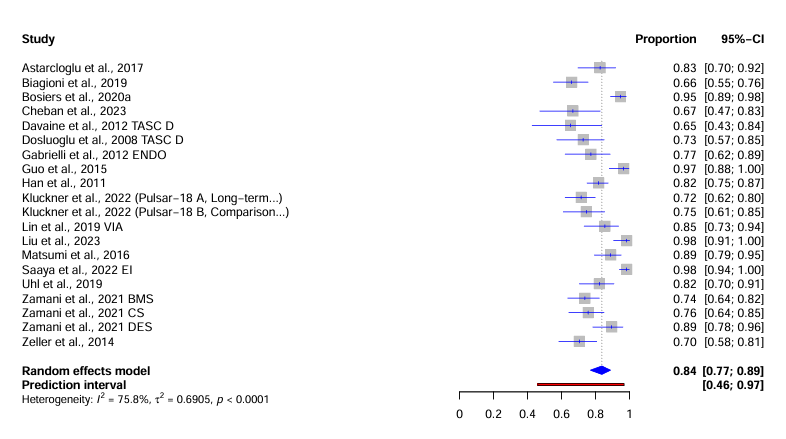


CI: Confidence Interval.

*Figure* S8*.25: 12-Month secondary patency, by lesion length*


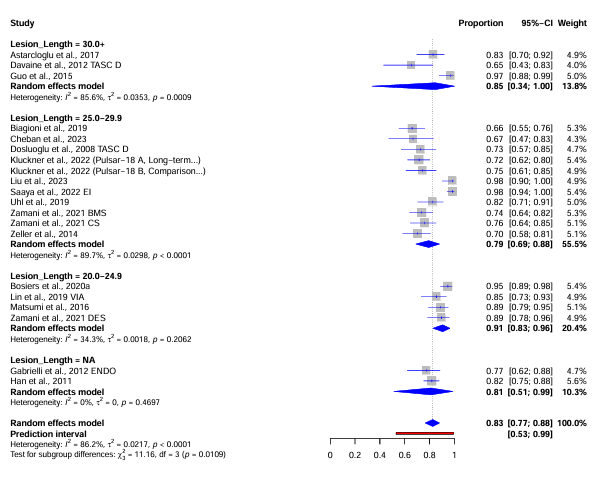


CI: Confidence Interval.

*Figure* S8*.26: 12-month secondary patency, by disease severity*


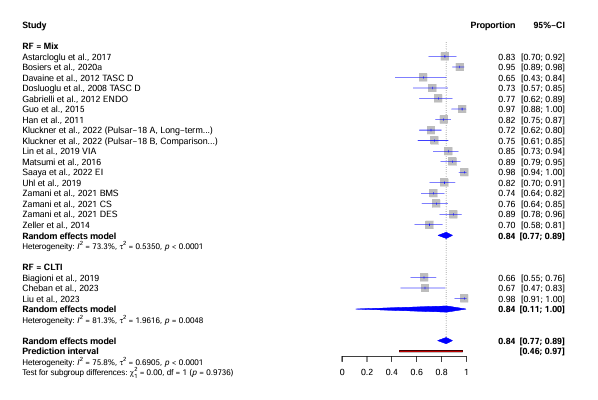


CI: Confidence Interval.

*Figure* S8*.27: 12-month secondary patency, by outcome definition*


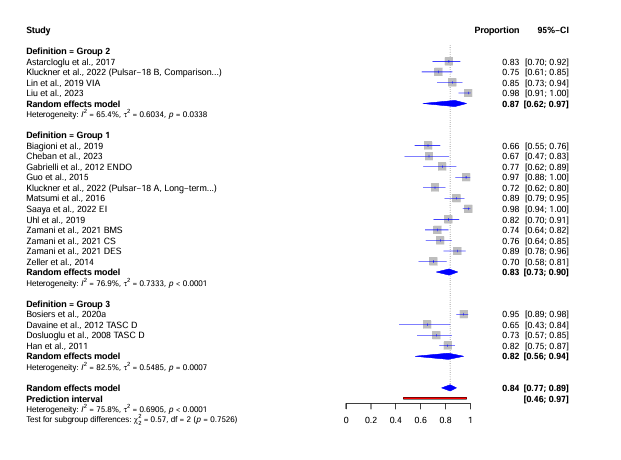


CI: Confidence Interval.

*Figure* S8*.28: 12-month secondary patency, by intervention*


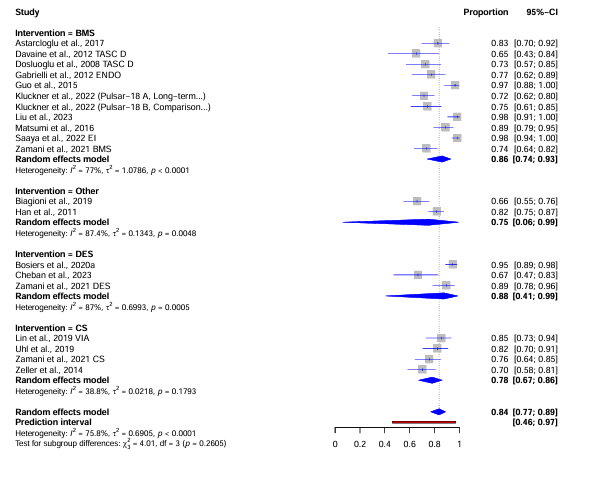


CI: Confidence Interval.

*Figure* S8*.29: 12-month secondary patency, by study design*


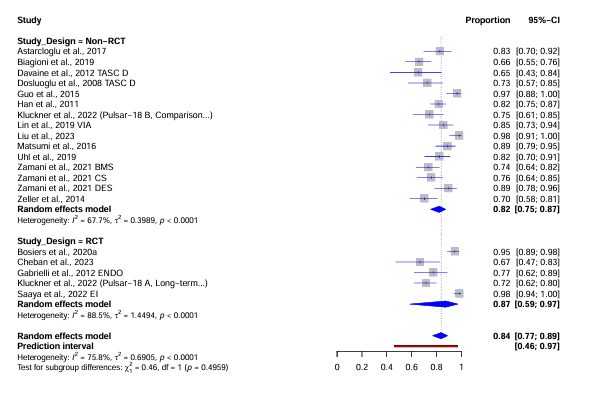


CI: Confidence Interval.

*Figure* S8*.30: 12-month secondary patency, by publication year*


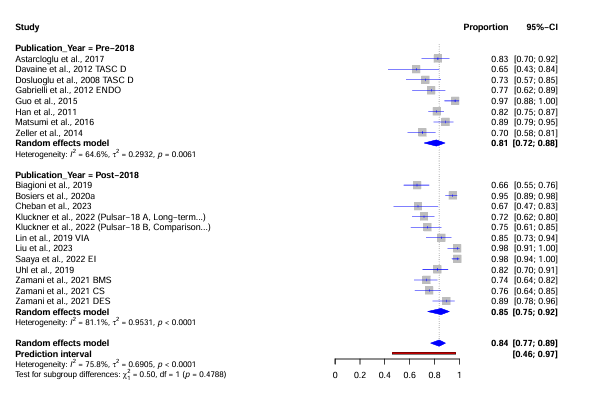


CI: Confidence Interval.

*Figure* S8*.31: 24-month secondary patency overall*


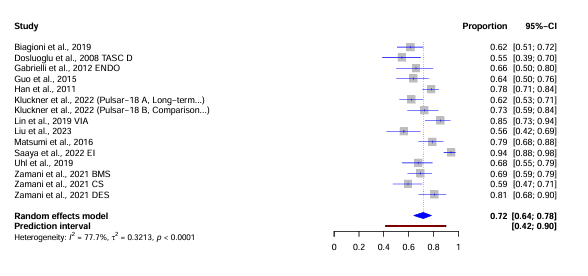


CI: Confidence Interval.

*Figure* S8*.32: 24-month secondary patency, by intervention*


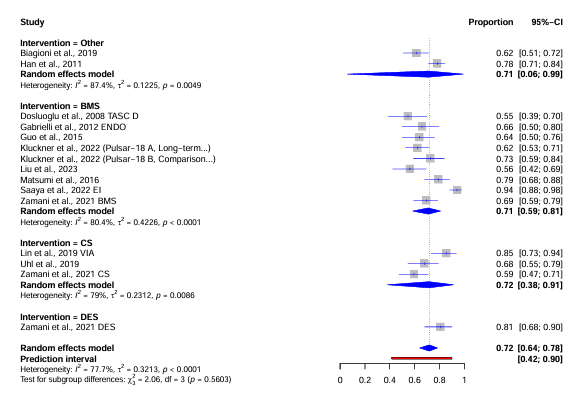


CI: Confidence Interval.

*Figure* S8*.33: 36-month overall secondary patency*


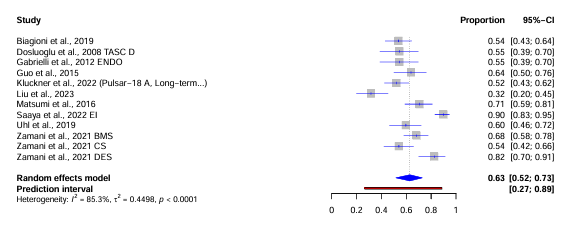


CI: Confidence Interval.

*Figure* S8*.34: 36-month secondary patency, by intervention*


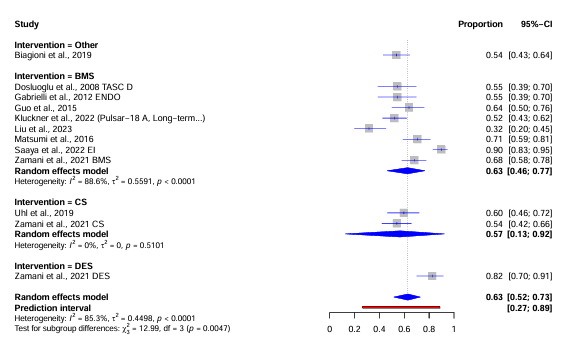


CI: Confidence Interval.

*Figure* S8*.35: 48-month secondary patency overall*


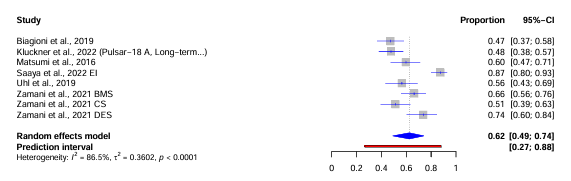


CI: Confidence Interval.

*Figure* S8*.36: 60-month secondary patency overall*


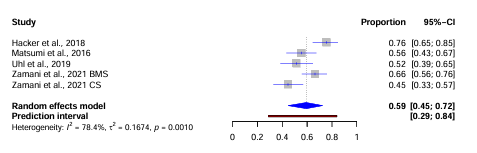


CI: Confidence Interval.

*Figure* S8*.37: 6-month overall assisted primary patency*


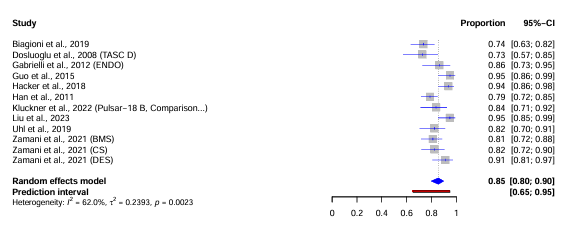


CI: Confidence Interval.

*Figure* S8*.38: 12-month overall assisted primary patency*


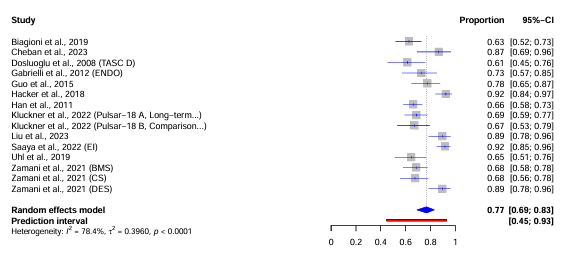


CI: Confidence Interval.

*Figure* S8*.39: 12-Month assisted primary patency, by lesion length*


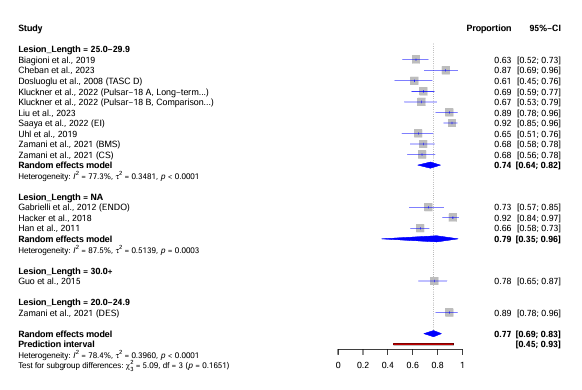


CI: Confidence Interval.

*Figure* S8*.40: 12-Month assisted primary patency, by disease severity*


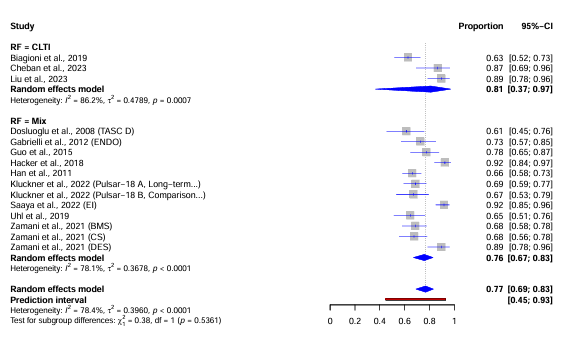


CI: Confidence Interval.

*Figure* S8*.41: 12-month assisted primary patency, by outcome definition*


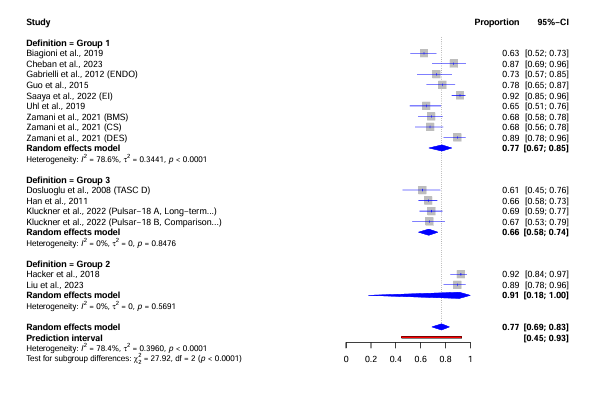


CI: Confidence Interval.

*Figure* S8*.42: 12-month assisted primary patency, by intervention*


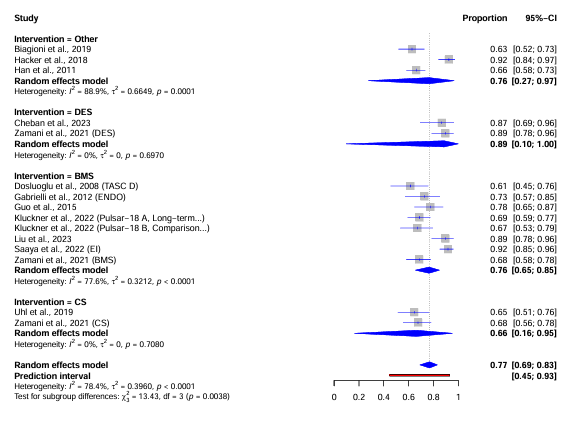


CI: Confidence Interval.

*Figure* S8*.43: 12-month assisted primary patency, by study design*


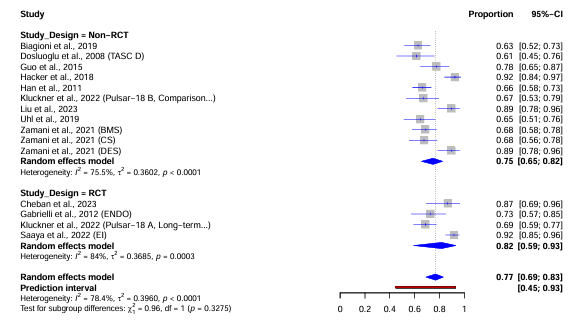


CI: Confidence Interval.

*Figure* S8*.44: 12-month assisted primary patency, by publication year*


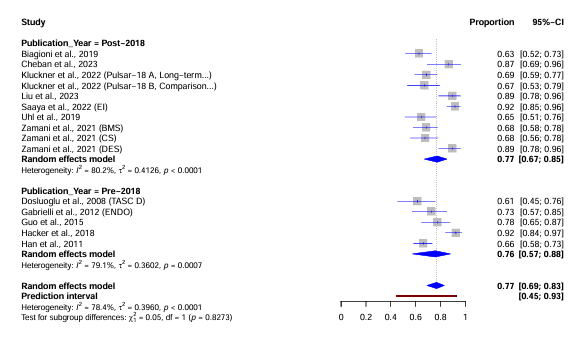


CI: Confidence Interval.

*Figure* S8*.45: 24-month assisted primary patency overall*


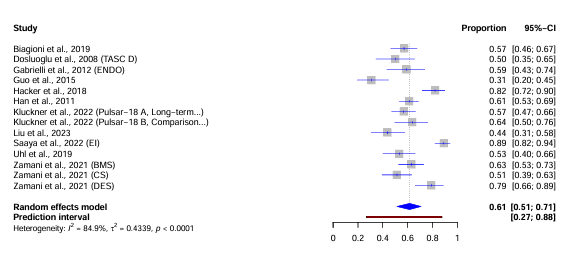


CI: Confidence Interval.

Figure S8.46: 36-month assisted primary patency overall


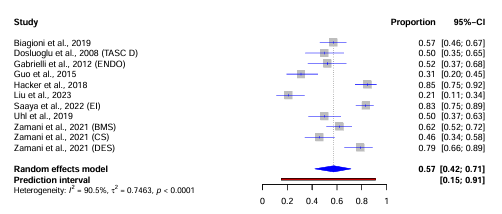


CI: Confidence Interval.

Figure S8.47: 48-month assisted primary patency overall


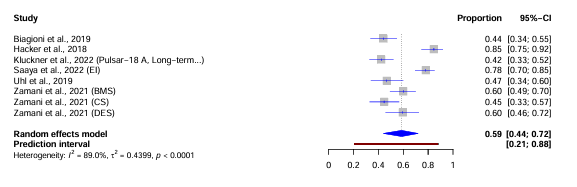


CI: Confidence Interval.

*Figure* S8*.48: 60-month assisted primary patency overall*


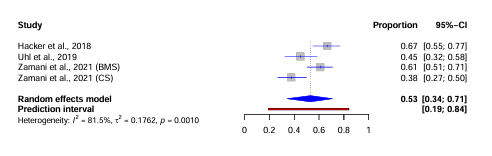


CI: Confidence Interval.

*Figure* S8*.49: 6-month ffTLR overall*


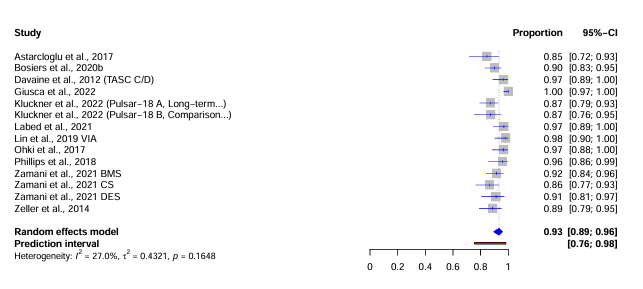


CI: Confidence Interval.

*Figure* S8*.50: 6-month ffTLR, by intervention*


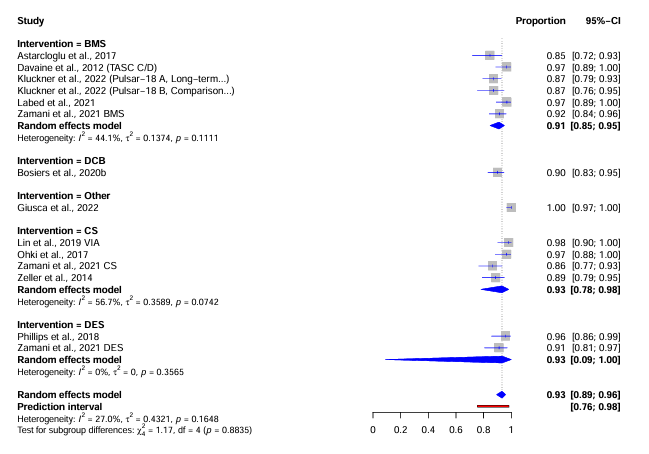


*Figure* S8*.51: 12-month ffTLR, overall*


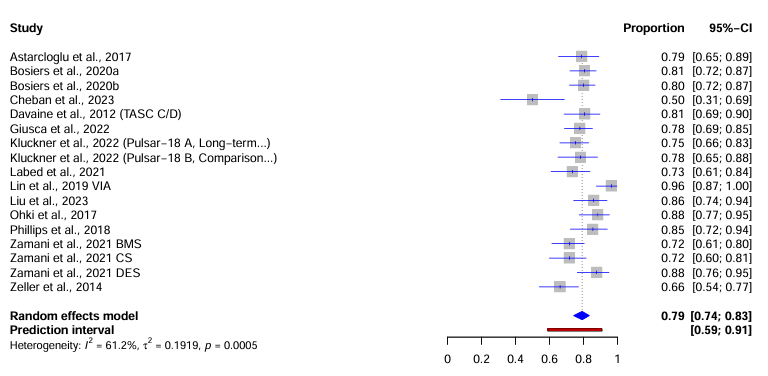


CI: Confidence Interval.

*Figure* S8*.52: 12-month ffTLR, by lesion length*


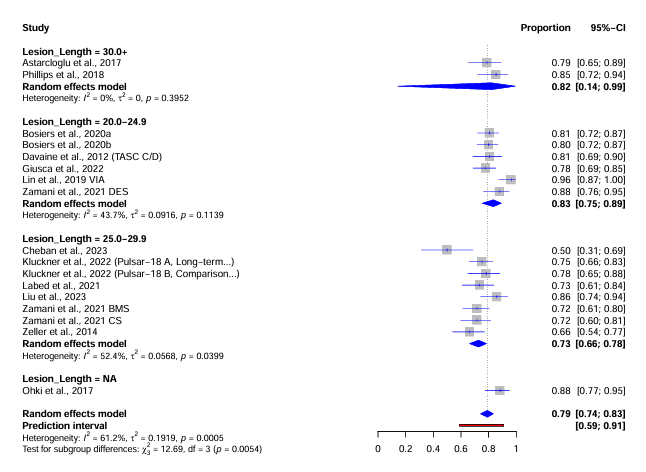


CI: Confidence Interval.

*Figure* S8*.53: 12-month ffTLR, by disease severity*


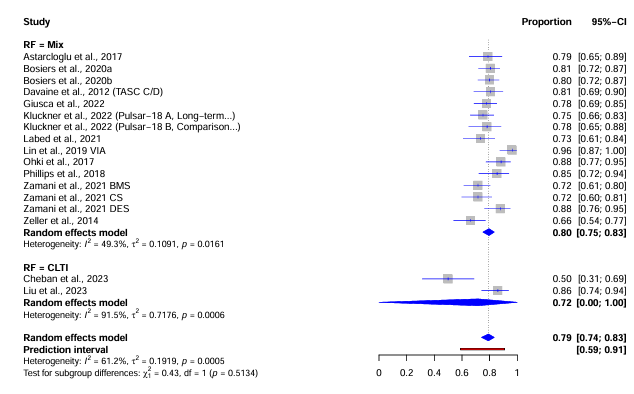


CI: Confidence Interval.

*Figure* S8*.54: 12-month ffTLR, by outcome definition*


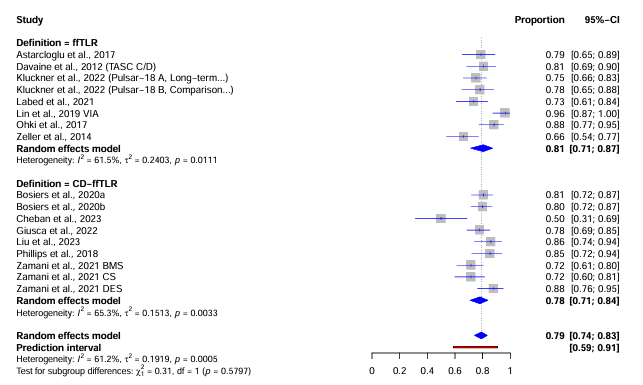


CI: Confidence Interval.

*Figure* S8*.55: 12-month ffTLR, by intervention*


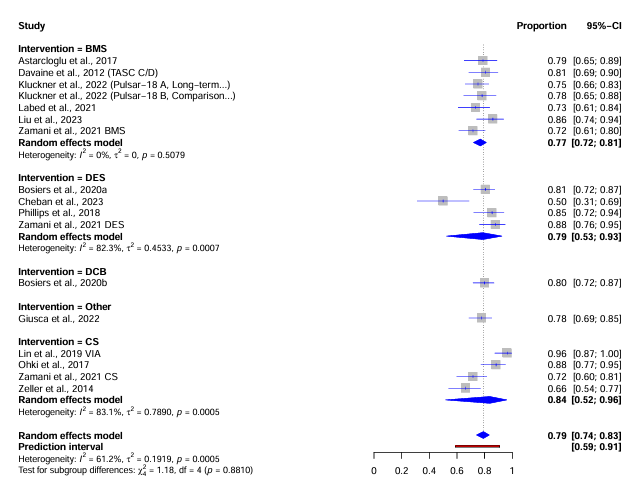


CI: Confidence Interval.

*Figure* S8*.56: 12-month ffTLR, by study design*


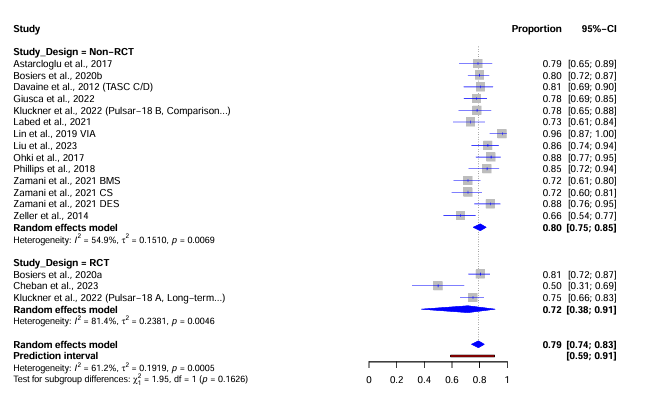


CI: Confidence Interval.

*Figure* S8*.57: 12-month ffTLR, by publication year*


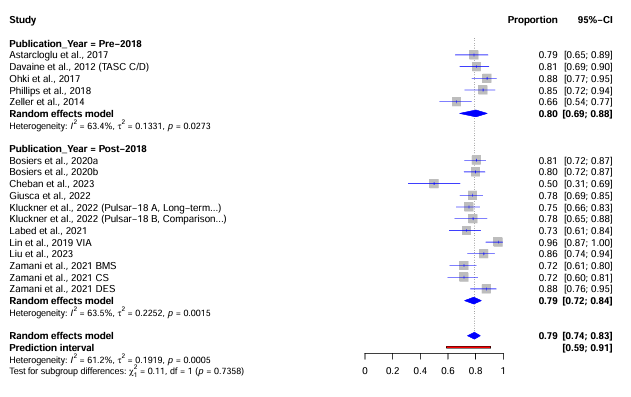


CI: Confidence Interval.

*Figure* S8*.58: 24-month ffTLR, overall*


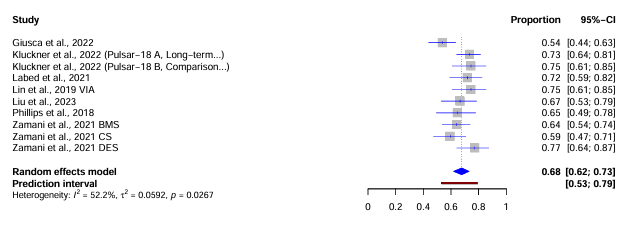


CI: Confidence Interval.

*Figure* S8*.59: 24-month ffTLR, by intervention*


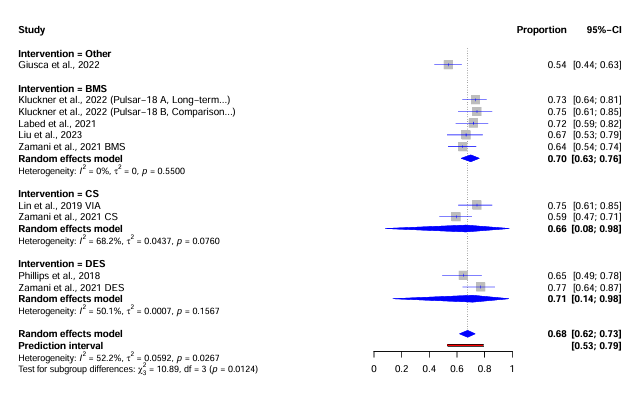


CI: Confidence Interval.

*Figure* S8*.60: 36-month ffTLR, overall*


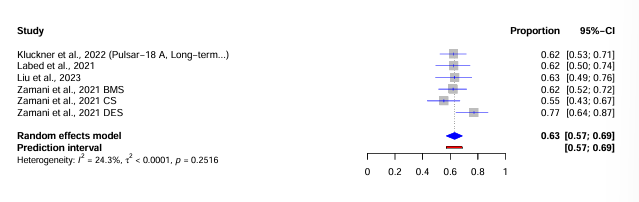


CI: Confidence Interval.

*Figure* S8*.61: 36-month ffTLR, by intervention*


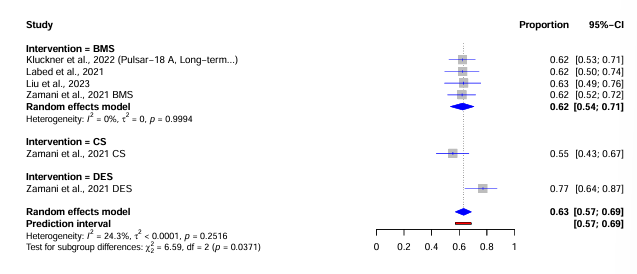


CI: Confidence Interval.

*Figure* S8*.62: 48-month ffTLR overall*


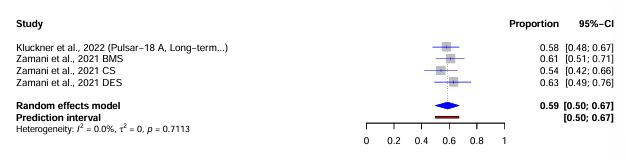


CI: Confidence Interval.

*Figure* S8*.63: 60-month ffTLR, overall*


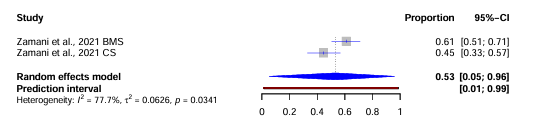


CI: Confidence Interval.

*Figure* S8*.64: 6-month Mortality, overall*


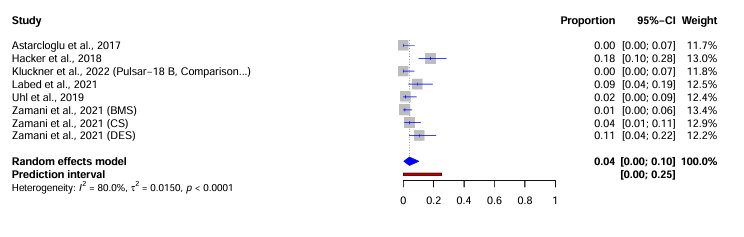


CI: Confidence Interval.

*Figure* S8*.65: 12-month overall Mortality*


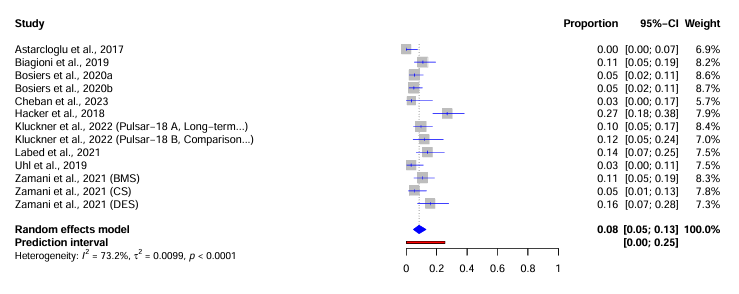


CI: Confidence Interval.

Figure S8.66: 12-month mortality, by lesion length


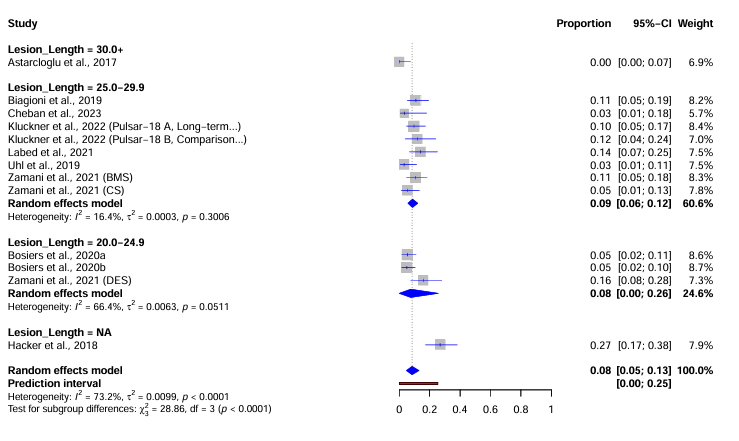


CI: Confidence Interval.

*Figure* S8*.67: 12-month mortality, by disease severity*


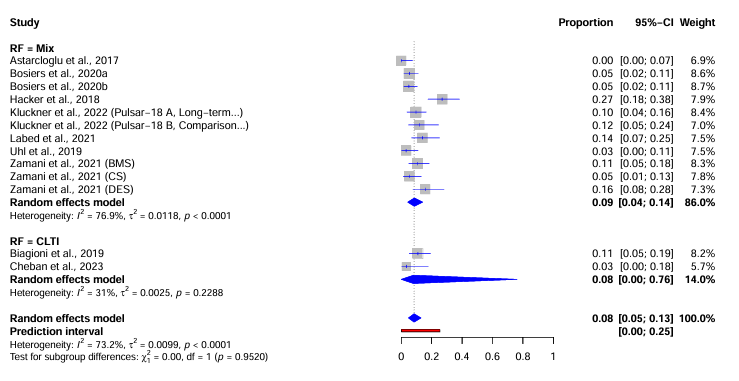


CI: Confidence Interval.

*Figure* S8*.68: 12-month mortality, by intervention*


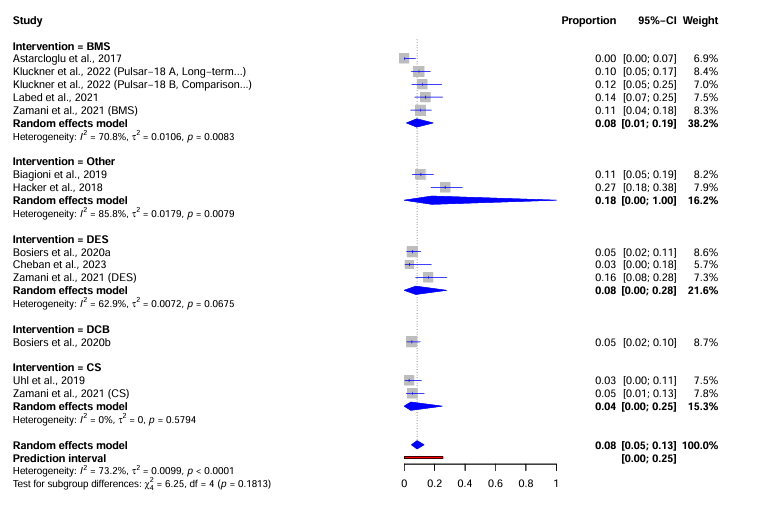


CI: Confidence Interval.

*Figure* S8*.69: 12-month mortality, by study design*


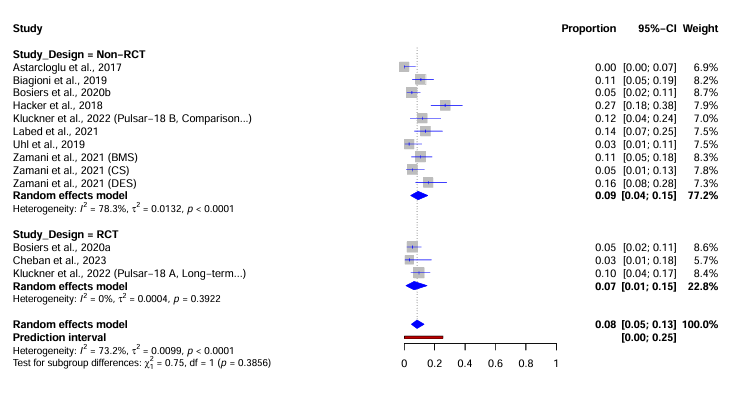


CI: Confidence Interval.

*Figure* S8*.70: 12-month mortality, by publication year*


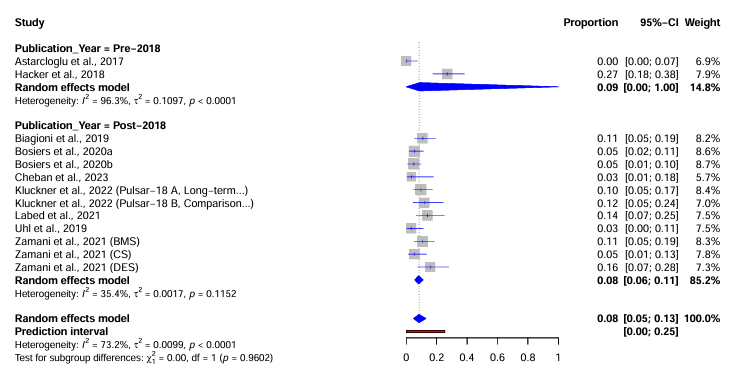


CI: Confidence Interval.

*Figure* S8*.71: 24-month mortality overall*


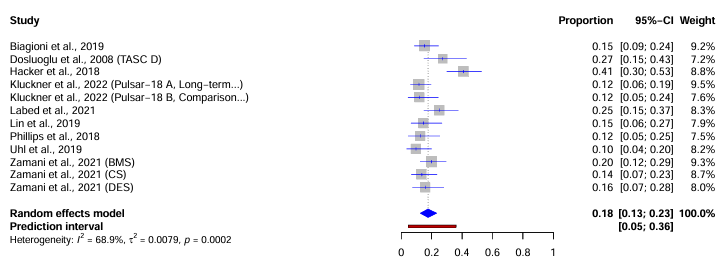


CI: Confidence Interval.

*Figure*  S8*.72: 36-month mortality overall*


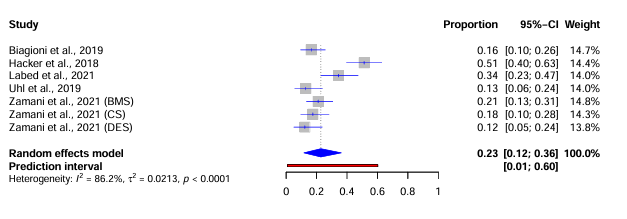


CI: Confidence Interval.

*Figure* S8*.73: 48-month mortality, overall*


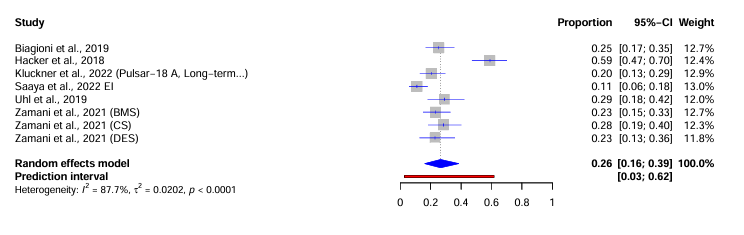


CI: Confidence Interval.

*Figure* S8*.74: 60-month mortality, overall*


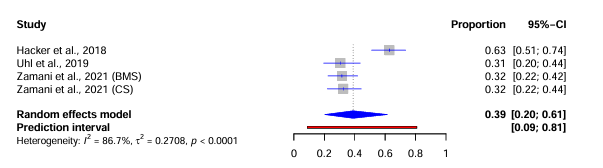


CI: Confidence Interval.

*Figure* S8*.75: 6-month limb salvage overall*


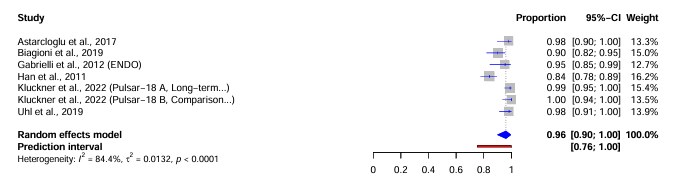


CI: Confidence Interval.

*Figure* S8*.76: 12-month overall limb salvage*


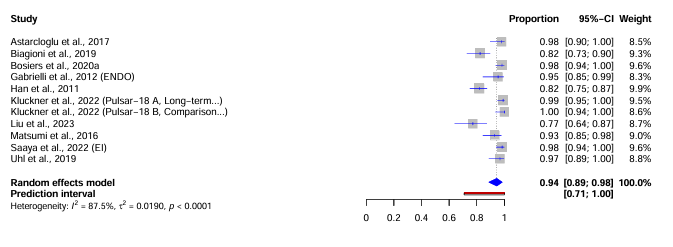


CI: Confidence Interval.

*Figure* S8*.77: 12-month limb salvage, by lesion length*


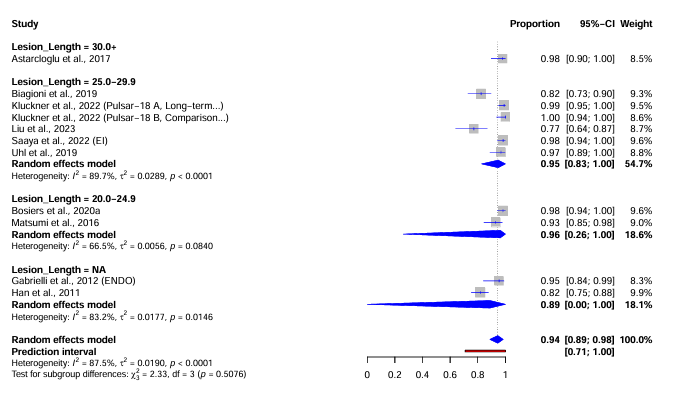


CI: Confidence Interval.

*Figure* S8*.78: 12-month limb salvage, by disease severity*


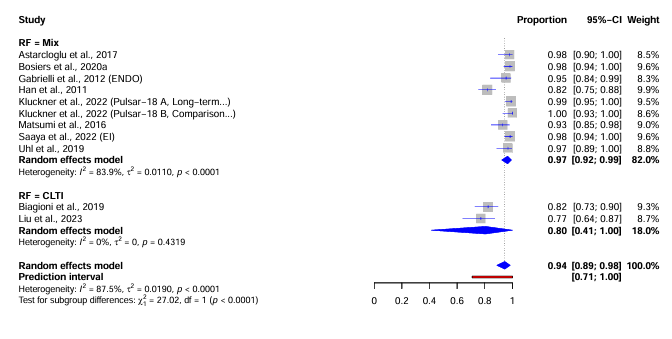


CI: Confidence Interval.

*Figure* S8*.79: 12-month limb salvage, by intervention*


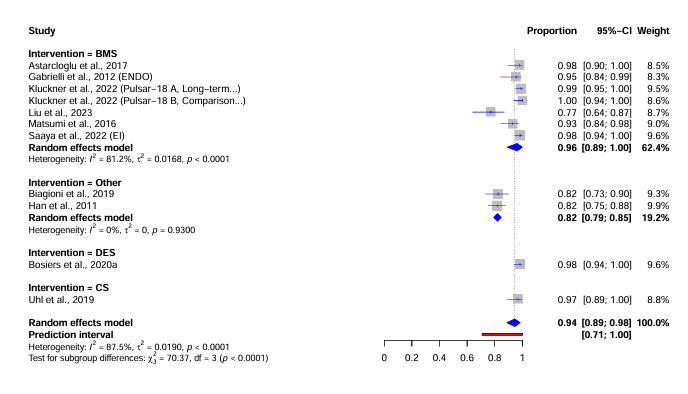


CI: Confidence Interval.

*Figure* S8*.80: 12-month limb salvage, by study design*


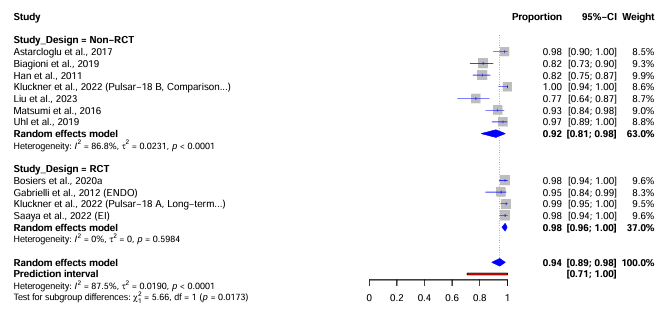


CI: Confidence Interval.

*Figure* S8*.81: 12-month limb salvage, by publication year*


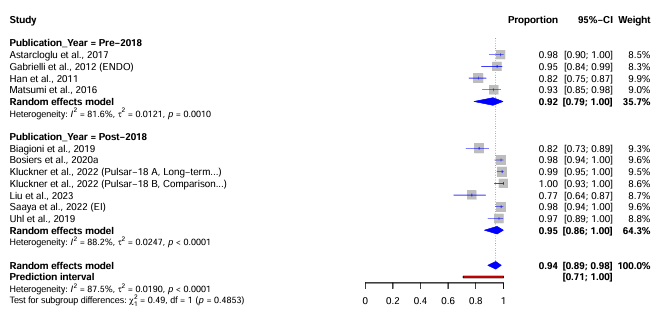


CI: Confidence Interval.

*Figure* S8*.82: 24-month overall limb salvage*


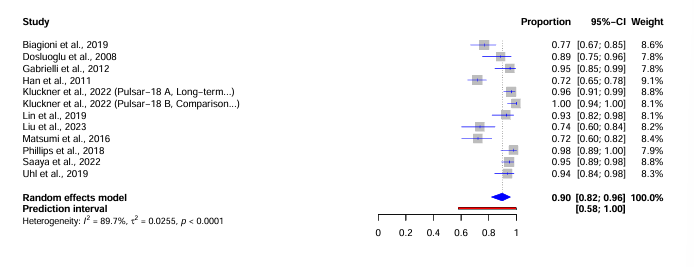


CI: Confidence Interval.

Figure S8.83: 24-month limb salvage, by disease severity


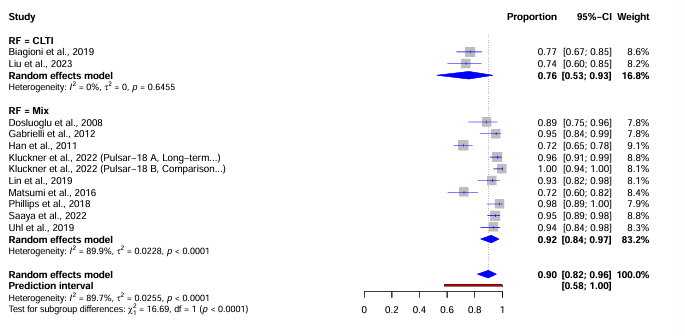


CI: Confidence Interval.

Figure S8.84: 36-month overall limb salvage


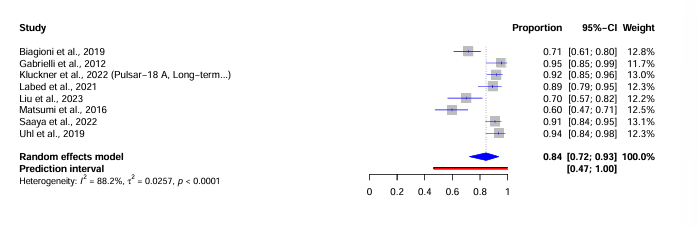


CI: Confidence Interval.

Figure S8.85: 36-month limb salvage, by disease severity


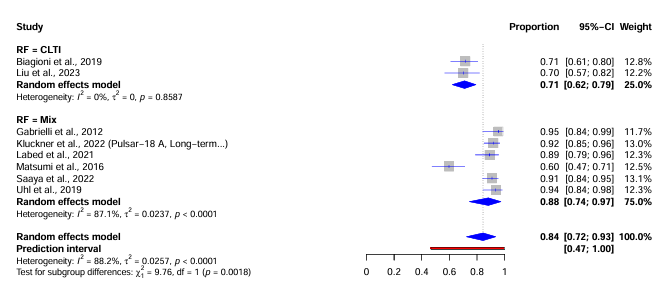


CI: Confidence Interval.

*Figure* S8*.86: 48-month overall limb salvage*


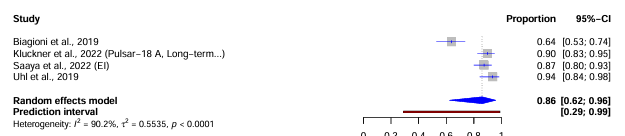


CI: Confidence Interval.

*Figure* S8*.87: 60-month overall limb salvage*


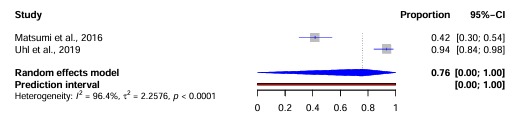


CI: Confidence Interval.

## **References**

1. Page MJ, McKenzie JE, Bossuyt PM, et al. The PRISMA 2020 statement: an updated guideline for reporting systematic reviews. Int J Surg. 2021;88:105906.
2. Giannopoulos S, Lyden SP, Bisdas T, et al. Endovascular intervention for the treatment of trans-atlantic inter-society consensus (TASC) D femoropopliteal lesions: a systematic review and meta-analysis. Cardiovasc Revasc Med. 2021;22:52-65.
3. AbuRahma AF, AbuRahma ZT, Scott G, et al. Clinical outcome of drug-coated balloon angioplasty in patients with femoropopliteal disease: a real-world single-center experience. J Vasc Surg. 2019;70(6):1950-1959.
4. AbuRahma AF, Beasley M, AbuRahma ZT, et al. Clinical outcome of drug-eluted stenting (zilver PTX) in patients with femoropopliteal occlusive disease a single center experience. J Endovasc Ther. 2022;29(3):350-360.
5. Astarcıoglu MA, Kılıt C, Sen T, et al. One-year results of primary stenting for TASC II D lesions of the superficial femoral and popliteal arteries. Acta Cardiol. 2017;72(1):36-40.
6. Biagioni RB, Brandao GD, Biagioni LC, Nasser F, Burihan MC, Ingrund JC. Endovascular treatment of TransAtlantic Inter-Society Consensus II D femoropopliteal lesions in patients with critical limb ischemia. J Vasc Surg. 2019;69(5):1510-1518.
7. Bosiers M, Setacci C, De Donato G, et al. ZILVERPASS study: ZILVER PTX stent vs bypass surgery in femoropopliteal lesions. J Endovasc Ther. 2020;27(2):287-295.
8. Bosiers M, Deloose K, Torsello G, et al. One-year outcome of the paclitaxel-eluting Legflow balloon catheter in the treatment of long and complex femoropopliteal lesions. J Card Surg. 2020;61(4):471-477.
9. Cheban AV, Osipova OS, Ignatenko PV, et al. One-year results of long femoropopliteal lesions stenting with fasciotomy lamina vastoadductoria. Ann Vasc Surg. 2023;88:100-107.
10. Davaine J-M, Azéma L, Guyomarch B, et al. One-year clinical outcome after primary stenting for Trans-Atlantic Inter-Society Consensus (TASC) C and D femoropopliteal lesions (the STELLA “STEnting Long de L'Artère fémorale superficielle” cohort). Eur J Vasc Endovasc Surg. 2012;44(4):432-441.
11. Dosluoglu HH, Cherr GS, Lall P, Harris LM, Dryjski ML. Stenting vs above knee polytetrafluoroethylene bypass for TransAtlantic Inter-Society Consensus-II C and D superficial femoral artery disease. J Vasc Surg. 2008;48(5):1166-1174.
12. Gabrielli R, Rosati MS, Vitale S, et al. Randomized controlled trial of remote endarterectomy versus endovascular intervention for TransAtlantic Inter-Society Consensus II D femoropopliteal lesions. J Vasc Surg. 2012;56(6):1598-1605.
13. Guo X, Xue G, Huang X, et al. Outcomes of endovascular treatment for patients with TASC II D femoropopliteal occlusive disease: a single center study. BMC Cardiovasc Disord. 2015;15:1-6.
14. Hacker R, Marone L. Long-term results of endovascular femoropopliteal interventions. Int J Angiol. 2018;27(03):151-157.
15. Han DK, Shah TR, Ellozy SH, Vouyouka AG, Marin ML, Faries PL. The success of endovascular therapy for all TransAtlantic Society Consensus graded femoropopliteal lesions. Ann Vasc Surg. 2011;25(1):15-24.
16. Iida O, Ohki T, Soga Y, et al. Twelve-month outcomes from the Japanese post-market surveillance study of the Viabahn Endoprosthesis as treatment for symptomatic peripheral arterial disease in the superficial femoral arteries. J Endovasc Ther. 2022;29(6):855-865.
17. Kluckner M, Nierlich P, Hitzl W, et al. Long-term results of endovascular treatment with nitinol stents for femoropopliteal TASC II C and D lesions. Medicina. 2022;58(9):1225.
18. Labed P, Gonzalez F, Jayet J, Javerliat I, Coggia M, Coscas R. Endovascular treatment of long femoropopliteal lesions with contiguous bare metal stents. Ann Vasc Surg. 2021;76:276-284.
19. Lammer J, Zeller T, Hausegger KA, et al. Heparin-bonded covered stents versus bare-metal stents for complex femoropopliteal artery lesions: the randomized VIASTAR trial (Viabahn endoprosthesis with PROPATEN bioactive surface [VIA] versus bare nitinol stent in the treatment of long lesions in superficial femoral artery occlusive disease). J Am Coll Cardiol. 2013;62(15):1320-1327.
20. Lin T-C, Chen P-L, Lee C-Y, Shih C-C, Chen I-M. Covered stent versus bare-metal stents for chronic total occluded long complicated femoropopliteal lesions: a 2-year single center review. J Chin Med Assoc. 2019;82(1):44-49.
21. Liu P, Zheng L-h, He X-q, et al. Midterm outcomes of endovascular therapy for TASC II D femoropopliteal lesions with critical limb ischemia: a retrospective analysis. Ann Vasc Surg. 2023;88:182-190.
22. Matsumi J, Ochiai T, Tobita K, et al. Long-Term Outcomes of Self-Expandable Nitinol Stent Implantation With Intraluminal Angioplasty to Treat Chronic Total Occlusion in the Superficial Femoral Artery (TransAtlantic Inter-Society Consensus Type D Lesions). J Invasive Cardiol. 2016;28(2):58-64.
23. Ohki T, Kichikawa K, Yokoi H, et al. Outcomes of the Japanese multicenter Viabahn trial of endovascular stent grafting for superficial femoral artery lesions. J Vasc Surg. 2017;66(1):130-142. e1.
24. Phillips JA, Falls A, Kolluri R, et al. Full drug-eluting stent jacket: two-year results of a single-center experience with zilver PTX stenting for long lesions in the femoropopliteal arteries. J Endovasc Ther. 2018;25(3):295-301.
25. Saaya S, Osipova O, Gostev A, et al. A prospective randomized trial on endovascular recanalization with stenting versus remote endarterectomy for the superficial femoral artery total occlusive lesions. J Vasc Surg. 2022;76(1):158-164.
26. Uhl C, Dadras A, Reichmann F, et al. Long-term results of the heparin-bonded Viabahn stent graft in femoropopliteal TASC C and D lesions with a covered stent length of minimum 25 cm. Vascular. 2019;27(5):553-559.
27. Zamani N, Sharath SE, Browder RC, et al. Outcomes after Endovascular Stent Placement for Long-Segment Superficial Femoral Artery Lesions. Ann Vasc Surg. 2021/02/01/ 2021;71:298-307. doi:https://doi.org/10.1016/j.avsg.2020.08.124
28. Zeller T, Peeters P, Bosiers M, et al. Heparin-bonded stent-graft for the treatment of TASC II C and D femoropopliteal lesions: the Viabahn-25 cm trial. J Endovasc Ther. 2014;21(6):765-774.
29. Giusca S, Hagstotz S, Lichtenberg M, et al. Phoenix atherectomy for patients with peripheral artery disease. EuroIntervention. 2022;18(5):e432.
30. Kluckner M, Gratl A, Wipper SH, et al. Comparison of prosthetic and vein bypass with nitinol stents in long femoropopliteal lesions. Ann Vasc Surg. 2022;78:272-280.
31. McGuinness LA, Higgins JPT. Risk-of-bias VISualization (robvis): An R package and Shiny web app for visualizing risk-of-bias assessments. Res Synth Methods. 2020/04/26 2020;n/a(n/a)doi:10.1002/jrsm.1411
32. Sterne JA, Hernán MA, Reeves BC, et al. ROBINS-I: a tool for assessing risk of bias in non-randomised studies of interventions. BMJ. 2016;355
33. Egger M, Smith GD, Schneider M, Minder C. Bias in meta-analysis detected by a simple, graphical test. BMJ. 1997;315(7109):629-634.
34. Quality of Evidence In: Neumann I, Schünemann H, eds. The GRADE Book version 1.0 (updated September 2024). The GRADE Working Group. Accessed: 12 Februrary 2025. Available from: <https://book.gradepro.org>.
